# Immune–chondrocyte ligand–receptor axes in osteoarthritis: microenvironmental control of inflammation, matrix remodeling, and chondrocyte fate

**DOI:** 10.3389/fimmu.2026.1957574

**Published:** 2026-10-23

**Authors:** Ming Liu, Chenguang Zhong, Lingyue Yuan, Yuqi Tu, Kunhao Wang, Wenbo Wang

**Affiliations:** 1 Department of Orthopedics, The First Affiliated Hospital of Harbin Medical University, Harbin, China; 2 Department of Pathophysiology, Shenzhen University Medical School, Shenzhen University, Shenzhen, Guangdong, China

**Keywords:** chondrocyte, ligand–receptor axis, matrix remodeling, osteoarthritis, single-cell analysis, synovial inflammation

## Abstract

Osteoarthritis (OA) is increasingly recognized as a whole-joint disease shaped by chronic low-grade inflammation, matrix damage, mechanical stress, and failed tissue repair. Beyond the traditional view of cartilage wear, emerging evidence indicates that immune–chondrocyte ligand–receptor (LR) communication plays a central role in translating microenvironmental cues into disease-relevant chondrocyte phenotypes. In this narrative review, we summarize major immune-derived or inflammation-amplified LR axes that regulate OA progression, including cytokine pathways such as IL-1β–IL-1R, TNF-α–TNFR, IL-6–IL-6R/gp130, and IL-17–IL-17R; chemokine axes such as CXCL12–CXCR4 and CCL2–CCR2; DAMP–PRR pathways involving HMGB1, S100A8/A9, TLRs, and RAGE; and ECM-associated signaling through CD44 and integrins. These axes collectively drive chondrocyte catabolic activation, hypertrophic differentiation, senescence, inflammatory cell death, autophagy dysfunction, and impaired matrix repair. We further discuss how single-cell sequencing, spatial transcriptomics, multiplex imaging, and computational LR inference are redefining OA as a spatially organized disorder of immune–chondrocyte and multicellular joint communication. Finally, we highlight therapeutic opportunities, including receptor-level modulation, macrophage reprogramming, local intra-articular delivery, and LR-axis-based patient stratification. Understanding immune–chondrocyte LR networks may enable precision strategies that suppress pathological inflammation while preserving repair-oriented signaling in OA.

## Highlights

- Osteoarthritis is driven by immune–chondrocyte communication networks.
- Cytokine and chemokine axes reshape chondrocyte inflammatory states.
- DAMP–PRR signaling links matrix damage to innate immune activation.
- ECM-associated receptors connect mechanical stress with cartilage remodeling.
- Spatial omics may guide LR-axis–based precision therapy in osteoarthritis.

## 1 Introduction

Osteoarthritis (OA) is a multifactorial whole-joint disorder involving coordinated pathological changes in articular cartilage, synovium, subchondral bone, meniscus, infrapatellar fat pad, and other periarticular tissues (1, 2). Although cartilage degeneration remains a defining structural feature, its progression is influenced by abnormal mechanical loading, inflammatory signaling, extracellular matrix (ECM) damage, metabolic stress, and altered osteochondral and synovial crosstalk (3–6). Synovial inflammation is frequently detected in human OA and is associated with symptoms and structural progression, while macrophages, T cells, mast cells, and other immune populations contribute inflammatory mediators within the diseased joint (7–9). At the same time, damaged cartilage generates matrix fragments and danger-associated molecular patterns (DAMPs) that can further amplify innate inflammatory signaling (5, 10, 11). These observations support a bidirectional relationship between inflammatory joint microenvironments and cartilage dysfunction rather than a cartilage-centered model of OA.

Chondrocytes are the principal resident cells of articular cartilage and are responsible for maintaining ECM homeostasis. In OA, they become highly responsive to cytokines, chemokines, DAMPs, matrix-derived signals, and altered mechanical cues. Through receptors including IL-1R, TNFR, IL-6R/gp130, IL-17R, chemokine receptors, Toll-like receptors, RAGE, CD44, and integrins, chondrocytes translate microenvironmental signals into changes in matrix metabolism, inflammatory activity, senescence, hypertrophic remodeling, and cell survival (10, 12–16). However, these signaling pathways are often discussed separately, which can obscure the cellular source of the ligand, the receptor-bearing target, and the experimental evidence supporting communication between them.

The central question of this review is therefore: which ligand–receptor interactions link inflammatory or immune-associated joint signals to chondrocyte responses in OA, how strong is the evidence supporting these interactions, and how do they converge on disease-relevant cartilage phenotypes? We place particular emphasis on cytokine and chemokine axes with experimentally supported effects on chondrocytes, including IL-1β–IL-1R, TNF-α–TNFR, IL-6–IL-6R/gp130, IL-17–IL-17R, CXCL12–CXCR4, and CCL2–CCR2 signaling (15, 17–21). DAMP–pattern-recognition receptor pathways and selected ECM-associated receptor systems are also considered when they participate in inflammation-amplified feedback between tissue damage and chondrocyte dysfunction (22–24).

To maintain conceptual clarity, we distinguish four forms of communication. Direct immune cell–chondrocyte signaling refers to immune-cell–derived ligands acting on receptors expressed by chondrocytes; human OA tissue and single-cell studies support synovial immune populations as important sources of cytokines acting on cartilage (25). Stromal relay signaling refers to immune activation of synovial fibroblasts, adipose-associated cells, vascular cells, or osteochondral populations that subsequently generate secondary signals affecting chondrocytes (25, 26). Chondrocyte autocrine amplification describes ligands produced by activated chondrocytes that reinforce their own inflammatory state, as demonstrated for cytokine- and chemokine-driven autocrine/paracrine circuits in OA cartilage (27–29). Matrix-derived damage and mechanotransduction signaling include ECM fragments or altered matrix properties acting through PRRs, CD44, integrins, or mechanosensitive pathways (22, 24, 30). The latter three categories are not considered equivalent to direct immune cell–chondrocyte communication; rather, they are included when they amplify, modify, or provide context for immune-associated signaling within the OA joint.

Because evidence for individual ligand—receptor axes varies substantially, this review applies an evidence-weighted approach. We prioritize studies using human OA tissues, spatial localization, protein-level validation, direct co-culture or conditioned-medium systems, receptor- or ligand-specific functional perturbation, and genetic models. Findings based predominantly on cytokine-stimulated chondrocytes, broad pharmacological inhibition, animal models, or computational ligand—receptor inference are interpreted more cautiously. Single-cell and spatial studies are included primarily when they identify candidate sender—receiver relationships or anatomically localize communication modules, but computationally inferred interactions are not considered causal without functional validation.

Accordingly, this narrative review synthesizes the biological evidence for immune–chondrocyte ligand–receptor communication, with particular emphasis on the major chondrocyte outcomes on which these pathways converge and the experimental approaches used to validate these interactions. We also briefly consider translational implications while distinguishing clinically evaluated interventions from preclinical and hypothesis-generating strategies. This framework aims to position immune–chondrocyte communication as an important component of OA pathogenesis without reducing OA to a single inflammatory or cellular mechanism. As a narrative review, this article does not follow a formal systematic review or meta-analysis framework; rather, it provides a critical, evidence-weighted synthesis of representative mechanistic, translational, single-cell, and spatial studies relevant to immune-chondrocyte communication in OA.

## 2 Cellular landscape of immune–chondrocyte communication in OA

Immune–chondrocyte communication in OA occurs within a multicellular joint microenvironment involving articular chondrocytes, synovial immune and stromal cells, subchondral bone-associated cells, vascular cells, and the infrapatellar fat pad (25, 31, 32). Within this network, chondrocytes function as signal-receiving and signal-amplifying cartilage cells, immune populations provide inflammatory or regulatory cues, and stromal, vascular, and osteochondral populations serve as relay compartments that transmit or reshape these signals. This section therefore focuses on the cellular composition and functional organization of the communication network, whereas the specific ligand–receptor pathways and downstream signaling mechanisms are discussed in subsequent sections.

### 2.1 Chondrocytes as signal-receiving and signal-amplifying cartilage cells

Chondrocytes are the principal resident cells of articular cartilage and are responsible for maintaining ECM homeostasis. In OA, however, they become highly responsive to inflammatory, damage-associated, metabolic, and mechanical cues originating from the synovium, subchondral bone, infrapatellar fat pad, damaged ECM, and infiltrating immune cells (25, 33). Single-cell analysis of matched human OA synovium and cartilage has identified multiple disease-associated chondrocyte states and predicted extensive cytokine and growth-factor communication between synovial cells and distinct chondrocyte populations (25). Importantly, chondrocytes are not only recipients of external signals. Activated or stressed chondrocytes can release cytokines and chemokines that influence synovial immune and stromal cells, thereby reinforcing inter-tissue communication; reciprocal signaling between human OA chondrocytes and synovial fibroblasts has, for example, been shown to amplify IL-6 production (34). Thus, chondrocytes can be viewed as both signal receivers and local amplifiers within the OA joint. The specific receptors, intracellular pathways, and resulting catabolic, senescent, hypertrophic, and death-associated phenotypes are discussed in Sections 3–7.

### 2.2 Synovial macrophages as major immune communication hubs

Synovial macrophages are among the most prominent immune populations in OA synovium. Studies of human OA synovial tissue demonstrate macrophage infiltration and activation, while macrophage abundance and phenotype vary substantially among patients (9, 35). Human OA synovium–cartilage single-cell analysis further identified inflammatory macrophage populations as important sources of cytokines capable of communicating with chondrocytes (25). Within the communication network, macrophages therefore function as major immune signaling hubs that influence chondrocytes, synovial fibroblasts, endothelial cells, and other immune populations. Importantly, OA-associated macrophages are heterogeneous rather than uniformly pro-inflammatory. Human synovial studies have identified distinct macrophage phenotypes associated with inflammatory versus cartilage-remodeling features, and single-cell analyses further demonstrate substantial heterogeneity within the OA synovial immune compartment (31, 35). These observations suggest that macrophage functions may range from inflammatory amplification to phagocytic and tissue-remodeling activities depending on disease context. Detailed macrophage-derived ligand–receptor axes and defects in inflammation resolution are discussed in later sections.

### 2.3 Other immune populations contributing to the communication network

T cells provide an adaptive immune component to OA-associated synovial inflammation. Human OA synovial tissue contains T-cell infiltrates, although their abundance and functional relevance vary among patients (9). Experimental studies provide additional mechanistic evidence: CD4+ T cells promoted disease progression in a murine ACLT model (36), whereas altered Th17 and Th17-like regulatory T-cell populations were associated with disease severity in equine post-traumatic OA (37). These findings should therefore be interpreted according to species and experimental context rather than directly extrapolated to human OA. Mast cells are also present in human OA synovium. Histological studies of knee and hip OA have demonstrated increased synovial mast-cell numbers and associations with synovitis, disease severity, and clinical symptoms (38, 39). Their principal role within the communication network may be to amplify inflammatory, vascular, and pain-associated responses through the release of histamine, proteases, cytokines, lipid mediators, and neuroactive factors. Neutrophils are less prominent than macrophages in chronic OA but are detectable in human knee OA synovial fluid (40, 41). Synovial-fluid biomarker studies further support the presence of neutrophil-associated inflammatory activity in OA and its relationship with disease severity (42). Thus, neutrophils may contribute to selected inflammatory OA states through oxidative, proteolytic, and alarmin-associated mechanisms, although their role appears more context-dependent than that of synovial macrophages.

### 2.4 Stromal, osteochondral, vascular, and adipose compartments as relay nodes

Immune–chondrocyte communication is frequently indirect. Synovial fibroblasts, subchondral bone-associated cells, endothelial cells, and cells within the infrapatellar fat pad can receive inflammatory or mechanical signals and convert them into secondary cues that subsequently influence chondrocytes. These populations therefore function as relay compartments linking synovial inflammation, tissue remodeling, vascular changes, metabolic signals, and cartilage responses. Synovial fibroblasts are positioned at the interface between synovial inflammation and cartilage degeneration. Human OA studies demonstrate reciprocal chondrocyte–synovial fibroblast signaling, including amplification of IL-6 production (34), while single-cell analyses have identified distinct fibroblast subsets associated with inflammatory, fibrotic, and pain-related tissue states (31, 43). These observations support a role for synovial fibroblasts as heterogeneous stromal relay cells rather than passive structural components.

Subchondral bone-associated cells contribute to the network through osteochondral biochemical and mechanical crosstalk. Abnormal communication among osteoblasts, osteoclasts, osteocytes, vascular cells, and chondrocytes has been implicated in OA-associated subchondral remodeling and cartilage degeneration (44–46). Endothelial cells constitute an additional relay population. Abnormal angiogenesis occurs in both the synovium and the osteochondral junction in human knee OA (47). At the osteochondral junction, vascular channels are associated with increased nerve growth factor (NGF) expression and sensory nerve ingrowth, and sensory nerves within these channels have been linked to OA-related knee pain (48, 49). The infrapatellar fat pad represents another important inflammatory and metabolic compartment. It contains adipose, stromal, vascular, and immune populations capable of producing cytokines, adipokines, and other mediators relevant to OA (50). Its close anatomical and functional relationship with the synovial membrane has led to the proposal that these tissues form a morpho-functional unit (51). More recently, single-cell analysis of human infrapatellar fat pad and synovium has demonstrated shared mesenchymal lineage relationships and coordinated OA-associated inflammatory and fibrotic changes (32). Together, these cell populations form an interconnected communication network rather than a series of isolated cell–cell interactions. The major cellular sources, relay compartments, ligand classes, and chondrocyte outputs involved in this communication network are summarized in **Figure 1**.

**Figure 1:**
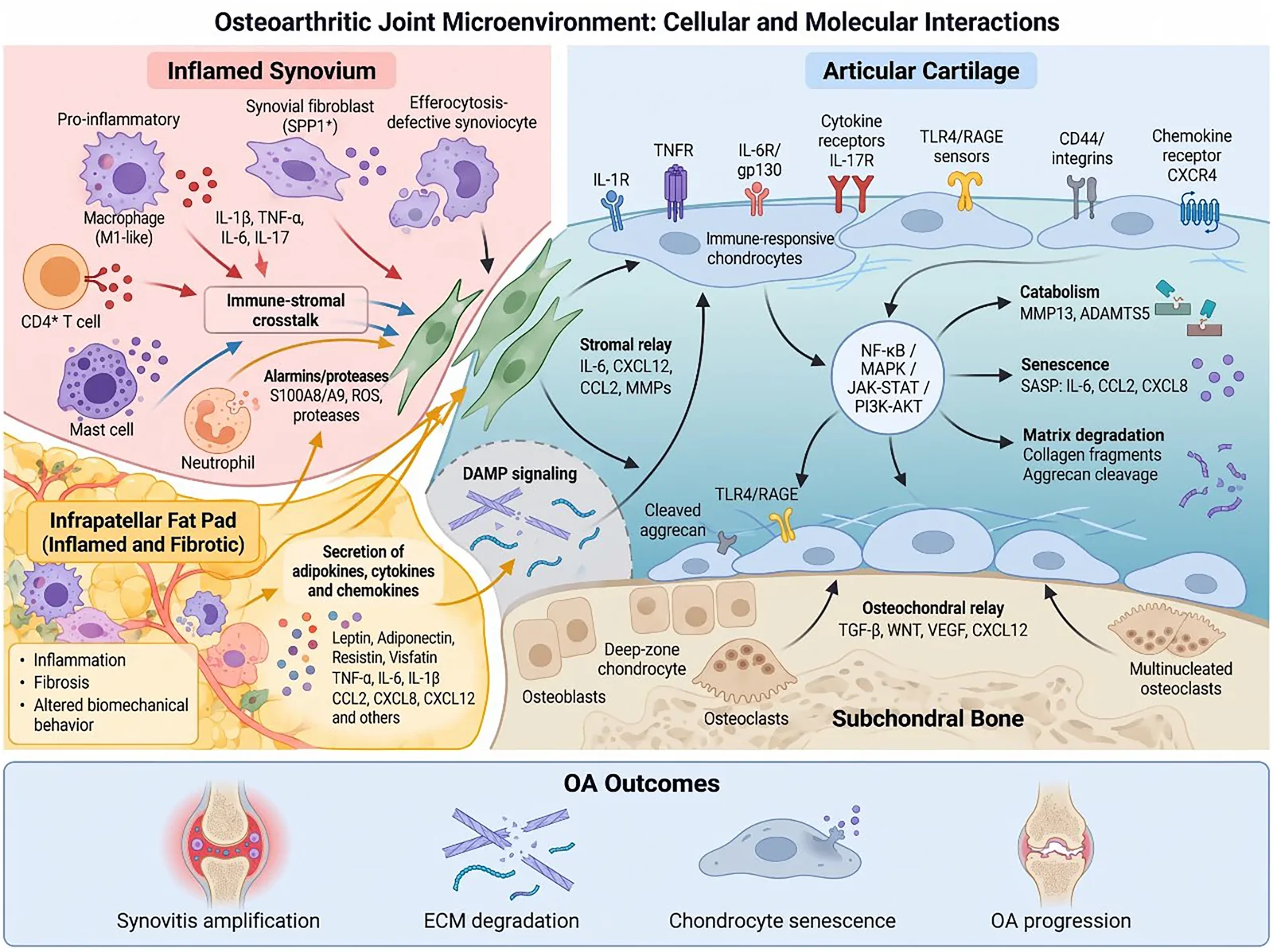
Cellular and tissue landscape of immune–chondrocyte communication in osteoarthritis. Immune cells, synovial fibroblasts, infrapatellar fat pad-associated cells, subchondral bone cells, vascular cells, and damaged extracellular matrix contribute soluble or matrix-derived signals that influence chondrocyte behavior. The figure distinguishes direct immune cell–chondrocyte signaling from stromal relay and matrix-derived communication and summarizes their convergence on inflammation, matrix catabolism, senescence, and impaired cartilage homeostasis.

## 3 Cytokine–receptor axes driving inflammatory and catabolic chondrocyte states

Cytokine–receptor signaling is a major component of immune–chondrocyte communication in OA. This section focuses on four representative axes—IL-1β–IL-1R, TNF-α–TNFR, IL-6–IL-6R/gp130, and IL-17–IL-17R—and examines how they contribute to chondrocyte dysfunction and cartilage degeneration. Representative ligand–receptor axes discussed throughout this review are summarized in **Supplementary Table S1**.

### 3.1 IL-1β–IL-1R axis: the classical inflammatory switch

The IL-1β–IL-1R axis is one of the most extensively studied inflammatory pathways in OA. In human OA synovium, CD14+ macrophage-enriched populations express substantially higher levels of IL-1β than CD14− fibroblast-enriched populations, and macrophage depletion from human OA synovial-cell cultures markedly reduces IL-1 production, supporting synovial macrophages as a major local source (52, 53). Human OA cartilage also contains IL-1β within chondrocytes, particularly in regions showing early degenerative changes, indicating that chondrocytes can provide an additional intra-cartilage source (27). Moreover, isolated human OA chondrocytes can express IL-1β under inflammatory stimulation *in vitro* (15). Thus, current evidence supports synovial macrophages and chondrocytes as relevant sources of IL-1β in OA, although their relative contributions are likely to vary according to joint compartment and inflammatory state.

### 3.2 TNF-α–TNFR axis: inflammation, apoptosis, and receptor modification

TNF-α is another major inflammatory cytokine implicated in OA pathogenesis. Human OA synovial studies identify macrophages as an important local source: depletion of CD14+ macrophages markedly reduces TNF-α production in OA synovial-cell cultures, and freshly isolated CD14+ macrophage-enriched fractions from human OA synovium express higher TNF-α levels than fibroblast-enriched fractions (52, 53). Chondrocytes may also contribute under specific conditions; cultured human OA chondrocytes exhibit low basal TNF-α expression that is markedly increased following exposure to cartilage debris, although this evidence is derived from an *in-vitro* model and should not be interpreted as demonstrating that chondrocytes are a dominant TNF-α source *in vivo* (54). These findings support synovial macrophages as a principal TNF-α–producing population in OA while indicating that damaged cartilage may provide additional context-dependent TNF-α signals.

### 3.3 IL-6–IL-6R/gp130 axis: inflammation, metabolism, pain, and senescence

IL-6 in OA originates from several joint-resident cell populations, with particularly strong evidence for chondrocytes and synovial fibroblasts. In samples obtained from patients with OA, reciprocal chondrocyte–synovial fibroblast communication promotes IL-6 secretion, and synovial fibroblasts from obese OA patients produce increased amounts of IL-6 that correspond to elevated synovial-fluid concentrations (34). Human articular chondrocytes isolated from normal and OA cartilage also produce IL-6 *in vitro*, with production markedly enhanced by inflammatory stimulation such as IL-1 (55). Human OA synovial membrane itself is an additional IL-6–producing compartment (56). The infrapatellar fat pad (IFP) represents another relevant local source of IL-6. Human OA IFP explants produce inflammatory mediators, including IL-6, and increased IL-6 expression and protein abundance have been detected in OA-associated IFP tissue (57, 58). Synovial macrophages may further amplify this circuit indirectly, because macrophage depletion from OA synovial-cell cultures reduces fibroblast-derived IL-6 production (53). Thus, IL-6 signaling in OA reflects reciprocal communication among cartilage, synovial fibroblasts, the IFP, and immune cells rather than production by a single dominant cellular source.

### 3.4 IL-17–IL-17R axis: T cell–associated inflammation and immunological senescence

The cellular-source evidence for IL-17 in OA should be interpreted more cautiously than that for IL-1β, TNF-α, or IL-6. In human knee and hip OA, IL-17A protein is detectable in synovial fluid, and higher concentrations have been associated with more inflammatory or severe OA phenotypes (59–61). Human knee OA synovial membrane and synovial fluid also contain Th17-polarized CD4+ T-cell populations (62, 63), supporting Th17 cells as a plausible local source of IL-17A. However, these observational studies do not establish the complete cellular origin of synovial IL-17A. Therefore, rather than broadly attributing OA-associated IL-17 to Tc17 cells, γδ T cells, and other innate-like lymphocytes without OA-specific evidence, we focus here on Th17-associated signaling while acknowledging that additional cellular sources require further direct validation.

### 3.5 Combinatorial cytokine modules rather than single mediators

Although individual cytokine axes are useful for mechanistic analysis, OA pathogenesis is not driven by a single cytokine acting alone. In the diseased joint, chondrocytes are exposed to mixtures of IL-1β, TNF-α, IL-6, IL-17, IL-18, chemokines, DAMPs, prostaglandins, and growth factors. These signals interact at multiple levels, including receptor expression, intracellular pathway convergence, transcription factor cooperation, and feedback mediator production. Therefore, cytokine ligand–receptor axes in OA should be understood as combinatorial modules rather than isolated linear pathways.

However, this network-based view is not fully captured by many commonly used *in-vitro* OA models, which rely on stimulation of isolated chondrocytes with a single inflammatory mediator, most frequently IL-1β or TNF-α. Although such reductionist models are valuable for dissecting individual signaling pathways, they cannot reproduce the simultaneous and dynamically changing combinations of cytokines, chemokines, DAMPs, matrix-derived signals, and cell–cell interactions present in the OA joint. More complex experimental systems have begun to address this limitation. For example, macrophage-conditioned medium produces chondrocyte responses that differ from those induced by defined cytokine stimulation in three-dimensional human cartilage models (64), and conditioned media from distinct macrophage phenotypes can differentially regulate human articular chondrocyte phenotypes (65). More recently, activated macrophage-conditioned medium has been used to establish inflammatory two- and three-dimensional models using primary human OA chondrocytes (66). Future studies should therefore incorporate macrophage–chondrocyte co-culture, conditioned-medium approaches, combinations of inflammatory and damage-associated stimuli, cartilage or osteochondral explants, and mechanically active three-dimensional models to more faithfully investigate combinatorial ligand–receptor signaling in OA.

Several forms of cytokine cooperation are particularly relevant. IL-1β and IL-17 can jointly enhance inflammatory mediator production and cartilage destruction (67). TNF-α and IL-17 can synergize to amplify NF-κB/MAPK-dependent gene expression. IL-1β and TNF-α can converge on similar catabolic pathways and reinforce MMP and ADAMTS induction. IL-6 may function downstream of IL-1β, TNF-α, and IL-17, while also feeding back through IL-6R/gp130 signaling to influence chondrocyte metabolism, senescence, and chemokine production. Cytokine-induced chemokines such as CCL2 and CXCL8 can recruit macrophages, T cells, or neutrophils, which then provide additional cytokines and DAMP-amplifying mediators. This network structure helps explain why targeting a single cytokine has often produced limited therapeutic success in OA. Blocking one mediator may not fully suppress the inflammatory program because parallel axes can compensate or maintain downstream pathway activation. For example, even if IL-1β signaling is reduced, TNF-α, IL-17, DAMP–TLR signaling, or IL-6 trans-signaling may continue to activate NF-κB, MAPK, and JAK/STAT pathways. Similarly, suppressing inflammation too broadly may interfere with repair-associated processes, because some cytokine or gp130-related pathways may contribute to regenerative or homeostatic responses under specific conditions. A ligand–receptor module perspective therefore encourages more precise therapeutic thinking. Instead of asking whether one cytokine is “the” driver of OA, it may be more useful to identify disease-stage-specific and patient-specific cytokine modules. Some patients may have IL-6/senescence-high OA, others may show IL-17/Th17-high inflammation, DAMP/TLR-high innate activation, or TNF/TNFR-associated apoptotic signaling. These inflammatory endotypes may require different interventions, biomarkers, and treatment windows. The key concept is that cytokine ligand–receptor axes in OA should be understood as combinatorial modules rather than isolated linear pathways. This perspective positions cytokine signaling as a dynamic immune–chondrocyte communication network that integrates synovial inflammation, cartilage damage, immune cell recruitment, chondrocyte fate transitions, and failed tissue repair. Cytokine–receptor axes provide a direct mechanistic link between synovial immune activation and chondrocyte catabolic reprogramming. As summarized in **Figure 2**, IL-1β, TNF-α, IL-6, and IL-17 activate distinct receptor modules but converge on overlapping inflammatory pathways that drive matrix degradation, anabolic suppression, senescence, and failed repair.

**Figure 2:**
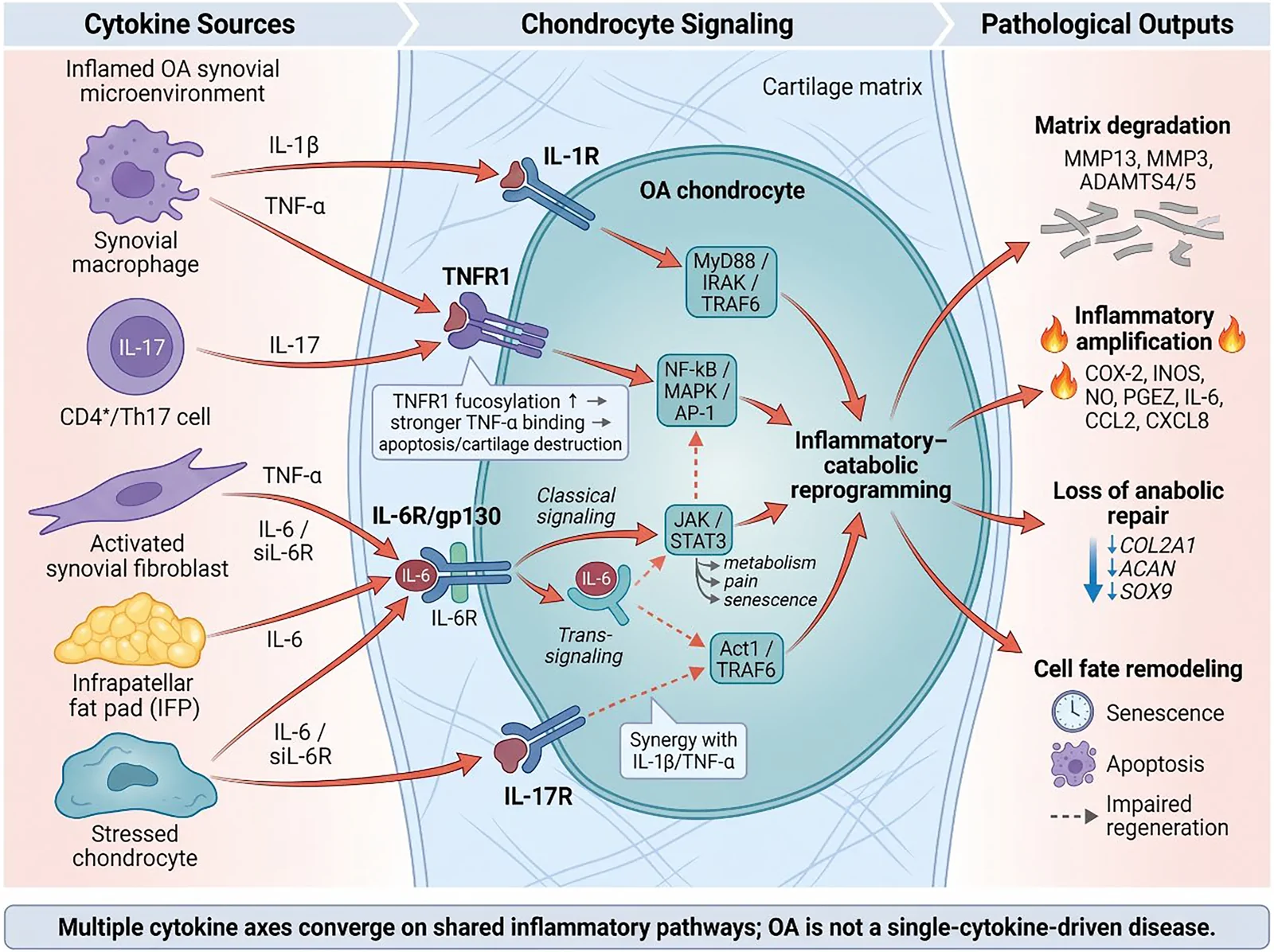
Major cytokine–receptor pathways influencing chondrocyte inflammatory and catabolic states in osteoarthritis. IL-1β, TNF-α, IL-6, and IL-17 derived from immune, stromal, and other joint-resident compartments engage their corresponding receptors on chondrocytes and activate NF-κB, MAPK/AP-1, JAK/STAT, and Act1/TRAF6 signaling. The infrapatellar fat pad (IFP) is included as an additional local source of IL-6. These pathways converge on matrix degradation, inflammatory amplification, impaired anabolic repair, senescence, apoptosis, and defective regeneration.

## 4 Chemokine–receptor axes linking synovitis to chondrocyte dysfunction

Chemokines are central mediators linking synovial inflammation to cartilage degeneration in OA (68, 69). Traditionally, chemokines are viewed mainly as leukocyte-recruiting molecules that guide monocytes, macrophages, T cells, neutrophils, and other immune cells into inflamed tissues (68, 70). In OA, however, chemokine signaling has broader significance. Chemokines not only organize immune-cell trafficking within the synovium but can also act directly on receptor-expressing chondrocytes and other joint-resident cells (28, 71). Human OA chondrocytes express multiple CC and CXC chemokine receptors, and receptor activation can directly induce matrix-degrading enzymes and other disease-associated phenotypic responses (28, 71). Chemokine signaling also contributes to interactions involving synovial stromal cells, subchondral bone-associated cells, and nociceptive pathways, thereby linking inflammatory recruitment with cartilage remodeling and OA-related pain (72, 73). For example, CCL2–CCR2 signaling promotes monocyte/macrophage recruitment, synovial inflammation, and cartilage damage in experimental OA, whereas CXCL12–CXCR4 signaling can directly influence chondrocyte function and cartilage degradation (20, 21). Unlike classical cytokines such as IL-1β and TNF-α, chemokines are particularly dependent on spatially organized concentration gradients that regulate cellular recruitment and positioning. Thus, chemokine–receptor networks provide a spatially organized communication system that can help sustain inflammatory niches and cartilage–synovium crosstalk within the OA joint (68). In OA joints, chemokines are produced by multiple cellular and tissue compartments, including chondrocytes, synovial fibroblasts, immune cells, endothelial cells, subchondral bone-associated cells, and the infrapatellar fat pad (IFP). Human OA studies demonstrate that the IFP is an active inflammatory tissue that expresses and releases chemotactic mediators, including CCL2/MCP-1 and CXCL8/IL-8 (57, 58, 74). Chemokine production can be further modulated by mechanical stress, inflammatory cytokines, DAMPs, ECM fragments, oxidative stress, and senescence-associated secretory programs. These chemokines contribute to immune-cell recruitment and synovial inflammatory amplification and may also influence receptor-expressing chondrocytes and other joint-resident cells.

### 4.1 CXCL12–CXCR4/CXCR7 axis

The CXCL12–CXCR4 axis is one of the most relevant chemokine pathways in OA and can be considered a key module connecting synovial inflammation, subchondral bone remodeling, chondrocyte dysfunction, and pain-related tissue changes. CXCL12, also known as stromal cell-derived factor-1, can be produced by synovial fibroblasts, subchondral bone cells, inflammatory cells, endothelial cells, and other stromal populations within the joint microenvironment (75–77). Its receptors include CXCR4 and CXCR7, also known as ACKR3, both of which can shape cellular responses in a context-dependent manner.

In OA, CXCR4 expression has been detected in chondrocytes and may be increased in damaged or senescent-like chondrocyte populations. This makes chondrocytes direct targets of CXCL12-rich inflammatory and stromal niches. Engagement of CXCL12 with CXCR4 can activate downstream pathways such as PI3K–AKT, MAPK, NF-κB-related signaling, and other stress-response programs (20, 78, 79). These pathways may promote inflammatory mediator production, matrix-degrading enzyme expression, hypertrophic differentiation, senescence-associated dysfunction, and altered survival responses. Through these effects, CXCL12–CXCR4 signaling can contribute to the shift of chondrocytes from a homeostatic matrix-producing state toward an inflammatory and catabolic phenotype.

The CXCL12–CXCR4 axis also provides an important link between cartilage and subchondral bone. Subchondral bone remodeling can alter CXCL12 production and create chemokine gradients across the osteochondral unit. These gradients may affect chondrocyte hypertrophy, osteochondral cross talk, angiogenesis-associated remodeling, and pain-related neurovascular changes. In this way, CXCL12–CXCR4 signaling is not restricted to synovitis; it connects multiple OA compartments, including synovium, cartilage, subchondral bone, blood vessels, and sensory nerve-associated structures.

CXCR7/ACKR3 adds another layer of complexity. Rather than functioning only as a classical chemokine receptor, CXCR7 can act as a scavenger, signaling-biased receptor, or modulator of CXCL12 availability. Depending on cellular context, CXCR7 may buffer CXCL12 gradients, alter CXCR4 signaling intensity, or activate distinct downstream pathways. The balance between CXCR4 and CXCR7 expression may therefore influence whether CXCL12 signaling promotes inflammation, tissue remodeling, cell survival, or repair-like responses. CXCL12 signaling through CXCR4 and CXCR7 can promote inflammatory and senescence-associated dysfunction in chondrocytes, thereby contributing to hypertrophic differentiation, ECM remodeling, and pain-associated tissue responses in OA. Therapeutically, CXCR4 antagonism has attracted attention as a potential chondroprotective approach. By interrupting CXCL12–CXCR4 signaling, it may be possible to reduce inflammatory chondrocyte activation, preserve cartilage matrix homeostasis, and limit synovitis–cartilage crosstalk (80). However, because CXCL12 also participates in tissue repair, progenitor cell trafficking, and vascular biology, complete or poorly timed blockade may have unintended effects. Future studies should clarify whether CXCL12–CXCR4/CXCR7 signaling is primarily pathogenic, compensatory, or stage-dependent in different OA endotypes.

### 4.2 CCL2–CCR2 axis

The CCL2–CCR2 axis is a major chemokine pathway involved in monocyte and macrophage recruitment. In OA, CCL2 is produced by synovial fibroblasts, macrophages, chondrocytes, endothelial cells, and adipose-associated stromal cells in response to inflammatory cytokines, DAMPs, mechanical stress, and ECM degradation products (81, 82). Its receptor CCR2 is highly relevant to circulating monocytes and inflammatory macrophage recruitment. Therefore, CCL2–CCR2 signaling provides a key mechanism by which local joint damage can recruit systemic immune cells and amplify synovitis (21, 83). Once recruited, CCR2-positive monocytes can differentiate into inflammatory macrophage-like cells in the synovium. These cells produce IL-1β, TNF-α, IL-6, chemokines, ROS, and proteases, thereby reinforcing inflammatory loops that affect chondrocytes. This process establishes a feed-forward circuit: damaged cartilage and activated synovium produce CCL2; CCL2 recruits CCR2-positive monocytes/macrophages; these macrophages release cytokines and catabolic mediators; chondrocytes respond by producing additional chemokines, matrix-degrading enzymes, and DAMPs. This loop sustains chronic low-grade synovitis and contributes to cartilage matrix breakdown.

Although the CCL2–CCR2 axis is most commonly discussed in relation to immune-cell recruitment, accumulating evidence indicates that it can also directly affect joint-resident cells. Human articular chondrocytes produce CCL2/MCP-1, and its expression is strongly enhanced by pro-inflammatory cytokines such as IL-1β and TNF-α (28, 84, 85). Importantly, both normal and OA chondrocytes express CCR2, and exogenous CCL2 directly activates ERK and p38 signaling through CCR2, increasing the expression of cartilage-degrading enzymes including MMP1, MMP3, and MMP13 (28, 86). CCL2–CCR2 signaling has also been implicated in chondrocyte hypertrophic differentiation, as CCL2 stimulation promotes COL10A1, RUNX2, and IHH expression in human OA chondrocytes, whereas inhibition of CCL2 or CCR2 attenuates these responses (87). Thus, although immune-cell recruitment remains its best-established function, the CCL2–CCR2 axis can also exert direct catabolic and phenotype-modifying effects on chondrocytes.

The strongest evidence for CCL2–CCR2 signaling in OA nevertheless derives from its role in monocyte/macrophage recruitment. CCL2 and other CCR2 ligands are elevated in human OA synovial fluid, CCR2-positive macrophages accumulate at sites of cartilage erosion, and genetic deletion or pharmacological blockade of CCL2/CCR2 reduces macrophage accumulation, synovitis, and cartilage damage in experimental OA (21). These findings support a model in which CCL2–CCR2 signaling reinforces the inflammatory microenvironment surrounding cartilage and thereby indirectly amplifies chondrocyte catabolism. The axis is also relevant to symptomatic OA. In experimental OA, CCR2 deficiency reduces movement-provoked pain and macrophage accumulation in the dorsal root ganglia, supporting a role for CCL2–CCR2 signaling in nociceptive sensitization (88). However, its relationship with structural disease is context-dependent: another post-traumatic OA study found that CCL2/CCR2 deficiency substantially altered pain-related behavior and early inflammatory gene expression but had relatively limited effects on chondropathy (89). Human cohort data further show associations between circulating CCL2 levels and knee OA outcomes, although such observational findings do not establish causality (90).

Macrophage accumulation may further amplify pain-associated signaling through the production of inflammatory cytokines, prostaglandin-related mediators, and nerve growth factor (NGF). Human OA synovial macrophages express inflammatory mediators and contribute to NGF production, providing a mechanistic link between synovial inflammation and nociceptive signaling (52). In obesity-associated OA, systemic metabolic inflammation may further modify chemokine-dependent myeloid responses; however, direct evidence establishing CCL2–CCR2 as a defining pathway of a specific obesity-associated OA endotype remains limited. Obesity itself is associated with inflammatory changes relevant to OA, whereas CCL2–CCR2 signaling is well established as a regulator of monocyte/macrophage recruitment in obese metabolic tissues (91, 92). These observations therefore support this connection as a plausible but incompletely validated context-dependent mechanism rather than an established OA endotype.

Within the ligand–receptor framework, the CCL2–CCR2 axis is therefore best regarded primarily as an immune-recruitment pathway with additional direct effects on chondrocytes. Therapeutically, modulation of this pathway may help limit persistent macrophage accumulation and interrupt the synovitis–cartilage degradation cycle (21). Nevertheless, translation requires caution because macrophage functions are heterogeneous and include not only inflammatory amplification but also debris clearance and resolution. Moreover, experimental studies indicate that the contribution of CCL2–CCR2 signaling to pain and structural cartilage damage can differ according to disease model and stage (88, 89). Thus, future therapeutic studies should define the disease context, cellular source of CCL2, and CCR2-expressing target populations before considering patient-specific intervention.

### 4.3 CXCL8–CXCR1/2 and neutrophil-associated axes

CXCL8 (IL-8) is detectable in the synovial fluid of patients with knee OA and has been associated with local inflammatory activity and disease-related joint changes (93, 94). CXCL8 is a major neutrophil-attracting chemokine that signals predominantly through CXCR1 and CXCR2 on neutrophils (95). Although OA synovial fluid is generally less neutrophil-rich than that of acute inflammatory arthritis, neutrophils are detectable in knee OA synovial fluid, and neutrophil-associated inflammatory biomarkers correlate with OA severity (41, 42).

Neutrophil-derived effector molecules may contribute to cartilage injury in selected inflammatory OA states. Myeloperoxidase (MPO) and MPO-derived chlorinated products have been detected at increased levels in early OA synovial fluid (96). Neutrophil elastase is also increased in OA synovial fluid and experimentally induces reactive oxygen species accumulation, mitochondrial dysfunction, and apoptosis in chondrocytes (97). These observations support a potential link between neutrophil activation and cartilage injury, although the relative contribution of neutrophils to chronic OA remains less established than that of macrophages.

S100A8 and S100A9 provide another mechanism connecting myeloid inflammation with cartilage catabolism. These alarmins are associated with synovial activation and joint destruction in human and experimental OA (98). Importantly, S100A8/A9 directly induce MMPs, IL-6, IL-8, and other catabolic mediators in human OA chondrocytes, and these effects are predominantly dependent on TLR4 signaling (99). Evidence implicating neutrophil extracellular traps (NETs) in OA is comparatively less mature; NET-related molecular signatures have been reported, but their direct causal contribution to cartilage degeneration requires further experimental validation (100).

CXCL8 may also be relevant to symptomatic OA. Higher synovial-fluid IL-8 concentrations have been associated with movement-related pain and clinical severity in patients with knee OA (101, 102). However, these observational associations do not by themselves establish direct CXCL8–CXCR1/2-mediated nociceptive sensitization. Thus, the CXCL8–CXCR1/2 axis is best viewed as a component of a broader neutrophil-associated inflammatory network whose contribution may be most relevant in selected inflammatory OA phenotypes.

### 4.4 CX3CL1–CX3CR1 axis

CX3CL1, also known as fractalkine, is a unique chemokine that exists in both membrane-bound and soluble forms (103, 104). Its receptor, CX3CR1, is expressed by monocytes, macrophages, T cells, NK cells, and selected tissue-resident immune populations. This axis is important because it can regulate both immune cell adhesion and chemotaxis, thereby supporting persistent immune cell positioning within inflamed tissues. In OA, CX3CL1–CX3CR1 signaling may connect immune cell localization with joint tissue inflammation. CX3CL1 expression in synovial or cartilage-associated tissues can attract or retain CX3CR1-positive macrophages and T cells. These recruited or retained immune cells may produce inflammatory cytokines, chemokines, and proteases that influence chondrocyte behavior (105–107). Therefore, the CX3CL1–CX3CR1 axis may help maintain synovial immune niches that sustain cartilage degeneration. This pathway may also be relevant to pain. In other inflammatory and neuroimmune contexts, CX3CL1–CX3CR1 signaling contributes to communication between tissue-resident cells and immune or neural cells. In OA, where synovitis, neurovascular remodeling, and pain sensitization are closely linked, fractalkine signaling may participate in inflammatory pain mechanisms. However, compared with IL-1β, IL-6, IL-17, or CXCL12, the direct role of CX3CL1–CX3CR1 in chondrocyte phenotype regulation is less clearly established. It is therefore best discussed as a supportive immune-positioning and pain-associated axis rather than as a primary direct chondrocyte-catabolic pathway.

### 4.5 Chemokines as dual regulators: recruitment and direct chondrocyte signaling

Chemokines in OA should not be viewed only as immune cell recruitment factors. They also function as tissue-organizing signals that influence the spatial distribution, persistence, and activity of inflammatory niches within the joint. Some chemokines primarily recruit immune cells into the synovium, whereas others can also act directly on receptor-bearing chondrocytes. This dual function places chemokines at the interface between synovitis and cartilage degeneration. The recruitment function is central to OA inflammatory amplification. CCL2–CCR2 promotes monocyte/macrophage recruitment; CXCL8–CXCR1/2 supports neutrophil-associated inflammatory responses; CX3CL1–CX3CR1 contributes to immune cell positioning and retention; and CXCL12–CXCR4 can influence immune, stromal, vascular, and osteochondral compartments. These recruited immune cells produce additional cytokines, DAMPs, ROS, and proteases that stimulate chondrocytes and accelerate matrix degradation.

The direct chondrocyte-signaling function is equally important. CXCL12–CXCR4 is a key example, as CXCR4 expression in OA chondrocytes and senescent chondrocytes suggests that chemokines can directly regulate chondrocyte dysfunction. Chemokine receptor activation may influence catabolism, hypertrophy, senescence, survival, and pain-associated tissue responses through MAPK, PI3K–AKT, NF-κB, and other pathways. Chondrocytes can also produce chemokines such as CCL2 and CXCL8, thereby recruiting immune cells and reinforcing synovitis. The IFP represents an additional local source of chemotactic mediators, including CCL2/MCP-1 and CXCL8/IL-8, further expanding the network of tissues capable of regulating immune-cell recruitment within the OA joint (57, 58, 74). Therefore, Chemokine signaling contributes to bidirectional communication between cartilage and the inflamed synovial microenvironment. Inflammatory stimuli and cartilage damage can enhance chemokine production by chondrocytes and synovial cells, promoting immune-cell recruitment into the joint. Recruited immune cells can then release additional cytokines and damage-associated mediators that further reinforce chondrocyte catabolism and senescence. This reciprocal amplification helps explain both the pathogenic importance of chemokine networks in OA and the challenges associated with therapeutically targeting individual chemokine axes. Inhibiting chemokine signaling may reduce inflammatory cell recruitment and protect cartilage, but chemokines also participate in tissue repair, progenitor cell trafficking, vascular remodeling, and immune surveillance. Future strategies should therefore define which chemokine axes are pathogenic in specific OA endotypes and disease stages. Chemokines in OA do not merely recruit inflammatory cells; they may also act directly on receptor-bearing chondrocytes to regulate catabolism, senescence, hypertrophy, and pain-related tissue responses. This dual role makes chemokine–receptor signaling a critical component of immune–chondrocyte ligand–receptor communication. Chemokine–receptor signaling links synovial immune recruitment with direct chondrocyte reprogramming. As illustrated in **Figure 3**, chemokines establish spatial gradients that recruit monocytes, macrophages, neutrophils, and T cells while also acting on receptor-expressing chondrocytes to promote catabolism, senescence, hypertrophy, and pain-associated remodeling.

**Figure 3:**
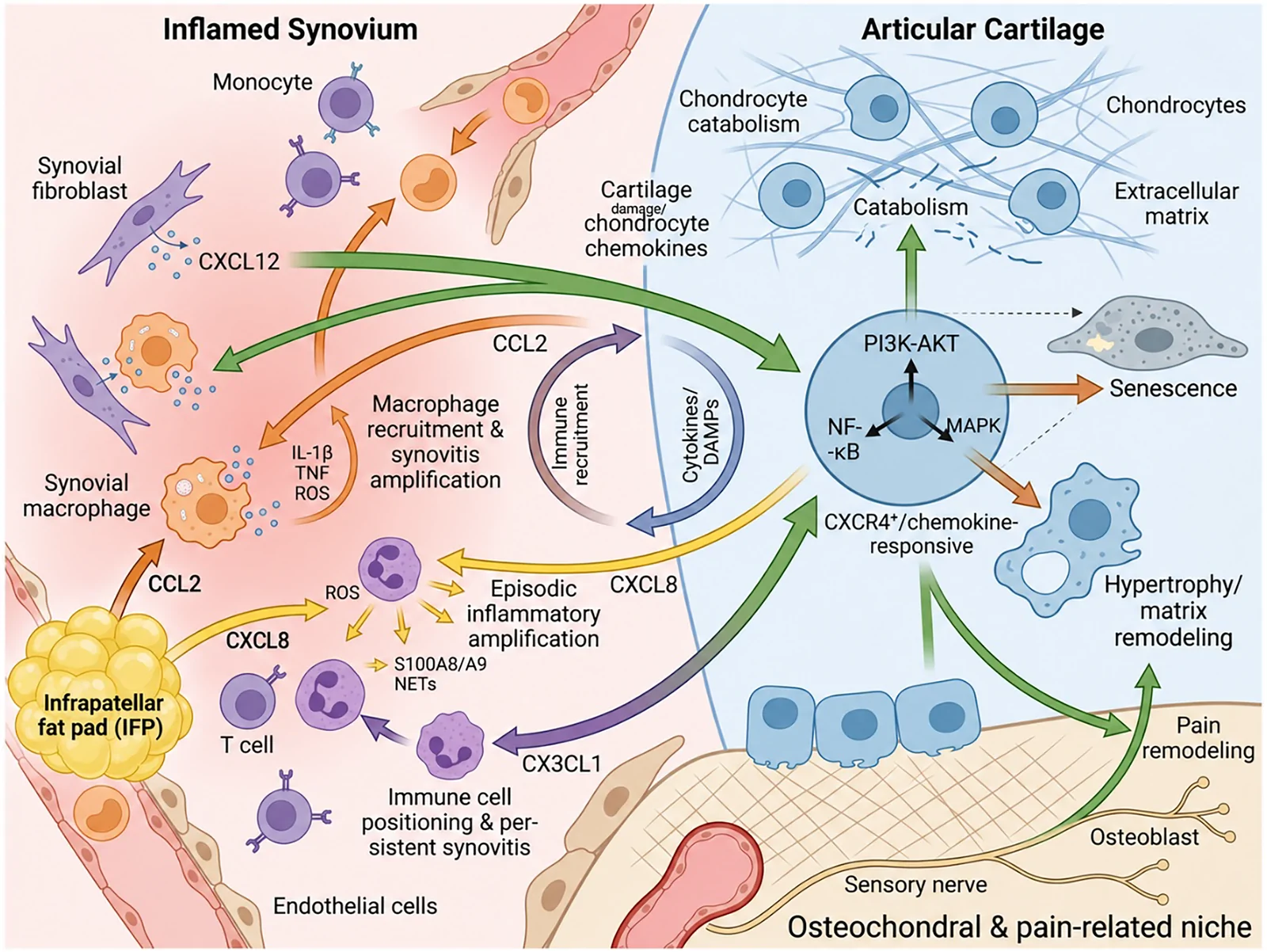
Chemokine–receptor networks linking joint inflammation with chondrocyte responses in osteoarthritis. Chemokines produced by synovial fibroblasts, immune cells, chondrocytes, vascular cells, osteochondral compartments, and the infrapatellar fat pad (IFP) regulate leukocyte recruitment and may also act on receptor-expressing chondrocytes. The IFP contributes chemokines including CCL2 and CXCL8, thereby supporting macrophage recruitment and episodic inflammatory amplification. Representative axes include CXCL12–CXCR4/CXCR7, CCL2–CCR2, CXCL8–CXCR1/2, and CX3CL1–CX3CR1, which are associated with inflammatory recruitment, cartilage catabolism, remodeling, and pain-related joint responses.

## 5 DAMP–PRR axes: converting matrix damage into innate immune activation

Damage-associated molecular pattern–pattern recognition receptor (DAMP–PRR) signaling provides a particularly important framework for understanding immune–chondrocyte communication in OA (108). Unlike classical cytokine pathways, which are mainly produced by activated immune or stromal cells, DAMPs are often generated directly from injured cartilage, stressed chondrocytes, degraded ECM, or mechanically damaged joint tissues (108). This makes DAMP–PRR signaling a bridge between structural tissue damage and innate immune activation. In OA, cartilage matrix degradation is not merely an endpoint of disease. Once ECM components are cleaved, fragmented, or released from damaged tissue, they can function as bioactive ligands that stimulate chondrocytes and other joint-resident cells (108, 109). These signals can engage innate and matrix-sensing receptors, including TLR2, TLR4, CD44, and integrins, while inflammatory stress can additionally promote inflammasome-associated signaling in chondrocytes (109–112). For example, fibronectin fragments activate TLR2-dependent catabolic signaling in human chondrocytes (109), whereas low-molecular-weight hyaluronan fragments induce inflammatory responses through both CD44 and TLR4 (110). HMGB1 is increased in OA tissues and can amplify inflammatory responses in patient-derived chondrocytes (113), while S100A8/A9 induce a pronounced TLR4-dependent catabolic response in human OA chondrocytes (99). As a result, structural cartilage injury can be converted into inflammatory signaling, creating a self-amplifying loop between matrix remodeling and innate immune activation. This section discusses major DAMP–PRR and damage-associated signaling axes in OA, including HMGB1-related signaling, S100A8/A9–TLR4-dominant signaling, ECM fragment–receptor interactions, and emerging alarmins such as 14-3-3ϵ (114).

### 5.1 Concept: DAMPs as ligands generated by cartilage injury

During OA progression, mechanical overload, oxidative stress, mitochondrial dysfunction, cellular senescence, apoptosis, pyroptosis, and ECM degradation all contribute to the release of DAMPs. These molecules are normally hidden within cells or structured ECM but become exposed or released after tissue injury. Once present in the extracellular joint space, they acquire signaling functions and act as endogenous ligands for innate immune receptors. Major DAMPs implicated in OA include HMGB1, S100A8/A9, fibronectin fragments, collagen fragments, tenascin-C, low-molecular-weight hyaluronan, biglycan, decorin, and 14-3-3ϵ (115–119). Some are intracellular alarmins released by stressed or dying cells, whereas others are ECM fragments generated by enzymatic degradation or mechanical damage. Despite their different origins, they share a common pathological function: they alert the joint microenvironment to tissue damage and activate inflammatory responses.

Chondrocytes are particularly important targets of DAMP signaling because they express multiple PRRs and matrix-sensing receptors. TLR2 and TLR4 recognize several ECM-derived ligands and alarmins. RAGE can respond to HMGB1 and S100 proteins. CD44 can bind hyaluronan fragments and osteopontin-related ligands. Integrins recognize altered matrix fragments and transmit mechanoinflammatory signals (120–122). In addition, DAMP-induced priming through NF-κB can support activation of NLRP3-related inflammasome pathways, leading to IL-1β and IL-18 production and inflammatory cell death programs. The pathological significance of DAMPs lies in their ability to create a feed-forward loop. Initial mechanical or inflammatory injury causes ECM breakdown and cell stress. This releases DAMPs. DAMPs then activate PRRs on chondrocytes and synovial cells. These cells produce cytokines, chemokines, MMPs, ADAMTS enzymes, ROS, nitric oxide, and prostaglandins. These mediators further degrade matrix and injure chondrocytes, releasing additional DAMPs.

### 5.2 HMGB1–TLR4/RAGE–related signaling

HMGB1 is one of the best-characterized alarmins involved in sterile inflammation and tissue injury. Under physiological conditions, HMGB1 is mainly a nuclear protein that regulates chromatin organization and gene transcription. Under stress, necrosis, pyroptosis, senescence, or inflammatory stimulation, HMGB1 can be released into the extracellular space, where it functions as a DAMP. In OA, increased HMGB1 expression or release has been observed in diseased cartilage and inflamed joint tissues, supporting its role as a mediator of damage-associated inflammation (123–127). Extracellular HMGB1 can engage several receptors, including TLR4 and RAGE, and may also interact with other PRR-related complexes depending on its redox state and molecular context. In chondrocytes, HMGB1-related signaling activates NF-κB and MAPK pathways, leading to increased expression of inflammatory mediators and matrix-degrading enzymes. This promotes the production of NO, PGE2, MMPs, cytokines, and chemokines, thereby linking chondrocyte stress to inflammatory amplification.

IL-1β stimulation can further enhance HMGB1-associated inflammatory responses. This is important because it places HMGB1 within a cytokine–DAMP amplification loop. IL-1β can induce chondrocyte inflammation and matrix degradation, while HMGB1 can reinforce NF-κB activation and sustain inflammatory mediator production (128, 129). In this way, HMGB1 does not simply act as a downstream marker of tissue damage; it actively participates in maintaining the OA inflammatory state. Pharmacological inhibition of HMGB1 has been shown to reduce IL-1β–induced inflammatory responses in OA chondrocytes, including reductions in NO, PGE2, inflammatory cytokines, and MMP production (113). This suggests that HMGB1 blockade may interrupt the DAMP-mediated amplification of cytokine-induced cartilage injury. In addition, the Nrf2/HMGB1/NF-κB axis has been implicated in chondrocyte apoptosis and ECM degradation, indicating that HMGB1 signaling also affects chondrocyte fate and matrix homeostasis (130).

### 5.3 S100A8/A9–TLR4-dominant signaling

S100A8 and S100A9 are calcium-binding proteins that often form the heterodimer calprotectin. They are mainly produced by neutrophils, monocytes, macrophages, and inflamed synovial tissues. In OA, S100A8/A9 can accumulate in synovial fluid and joint tissues, especially in inflammatory or damage-associated microenvironments (98, 99, 131, 132). Like HMGB1, S100A8/A9 can function as alarmins that convert immune cell activation and tissue stress into innate inflammatory signaling.

In human OA chondrocytes, S100A8 and S100A9 induce a pronounced catabolic response characterized by increased MMPs and inflammatory mediators and reduced expression of cartilage matrix components. Receptor-blocking experiments indicate that these effects are predominantly dependent on TLR4, whereas RAGE blockade does not substantially suppress the S100A8/A9-induced catabolic response (99). This axis is particularly relevant because it links neutrophil- and macrophage-associated inflammation with chondrocyte catabolism. Although OA is not typically dominated by acute neutrophil infiltration, neutrophil-derived alarmins and myeloid cell–derived S100 proteins can still contribute to sterile inflammatory amplification (97, 133, 134). S100A8/A9 may also promote recruitment and activation of additional myeloid cells, thereby reinforcing synovial inflammation and maintaining a DAMP-rich environment around cartilage.

S100A8/A9 signaling through TLR4 and RAGE activates downstream NF-κB and MAPK pathways, promoting the expression of matrix-degrading enzymes, inflammatory cytokines, COX-2, and chemokines that contribute to synovial inflammation and cartilage destruction. Compared with classical inflammatory cytokines such as IL-1β and TNF-α, S100A8/A9 is more closely associated with damage sensing and myeloid-driven sterile inflammation. This signaling axis therefore represents an important mechanism by which tissue stress and innate immune activation are translated into persistent inflammatory and catabolic responses in OA, thereby linking synovial inflammation, cartilage damage, and chondrocyte dysfunction.

### 5.4 ECM fragment–TLR/integrin/CD44 axes

ECM fragments represent another major category of DAMPs in OA (10). Articular cartilage is rich in type II collagen, aggrecan, fibronectin, hyaluronan, small leucine-rich proteoglycans, and other structural matrix components (111). During OA progression, these molecules are cleaved by MMPs, ADAMTS enzymes, mechanical stress, oxidative damage, and inflammatory proteases (135, 136). The resulting fragments are not biologically inert debris (137). Many function as endogenous ligands that activate PRRs, adhesion receptors, and matrix-sensing pathways (22). Fibronectin fragments are among the best-known matrix-derived inflammatory ligands (138). They can activate TLR2, TLR4, and integrin-related pathways in chondrocytes and synovial cells (108, 109, 139). This leads to NF-κB and MAPK activation, increased MMP expression, and further matrix degradation (140, 141). Collagen fragments can engage integrins and other matrix receptors, altering chondrocyte mechanotransduction and promoting catabolic signaling (142). Low-molecular-weight hyaluronan can signal through TLR2, TLR4, and CD44, whereas high-molecular-weight hyaluronan is generally associated with lubrication and anti-inflammatory or protective effects (110, 143, 144). This size-dependent difference is important because it shows that the same matrix family can produce either homeostatic or inflammatory signals depending on molecular structure (145).

Tenascin-C is another matrix molecule that can act as a DAMP. It can engage TLR4-related pathways and promote inflammatory activation in joint tissues. Biglycan and decorin, which are small leucine-rich proteoglycans, can also act as endogenous ligands for TLRs and contribute to sterile inflammation (146–149). These ECM-derived DAMPs are particularly important in OA because they directly link matrix remodeling to innate immune activation.

ECM degradation generates bioactive matrix fragments that can engage TLR2, TLR4, CD44, and integrins on chondrocytes and other joint-resident cells (109–111). For example, fibronectin fragments activate MyD88-dependent TLR2 signaling in human chondrocytes, whereas small hyaluronan fragments induce inflammatory responses through both TLR4 and CD44 (109, 110). Matrix-derived signals can subsequently activate NF-κB and MAPK pathways and increase the production of MMPs, ADAMTS enzymes, and inflammatory mediators (109, 140, 150). These responses further accelerate matrix breakdown and generate additional matrix-derived signals, establishing a self-reinforcing cycle of cartilage degeneration (151, 152). ECM fragment–receptor interactions also link the biochemical and biomechanical dimensions of OA. Chondrocyte integrins function as matrix sensors and mechanotransducers, and disruption of the ECM alters integrin-dependent signaling and cellular responses to mechanical cues (111, 153). In particular, fibronectin fragments acting through integrin-associated pathways activate FAK/PYK2, Src, MAPKs, and NF-κB, thereby promoting MMP expression and cartilage catabolism (140, 154, 155). Mechanically responsive transcriptional regulators such as YAP/TAZ provide an additional link between extracellular mechanical cues and chondrocyte anabolic–catabolic balance (156). Thus, matrix fragments are not merely products of cartilage destruction but can actively modify chondrocyte behavior and reinforce inflammatory and catabolic phenotypes (111, 157).

### 5.5 14-3-3ϵ as an emerging alarmin

Beyond classical DAMPs such as HMGB1, S100A8/A9, and ECM fragments, newer alarmins are being identified in OA. One notable example is 14-3-3ϵ, which has been proposed as a potential alarmin capable of activating innate immune responses in OA (114). The 14-3-3 protein family is traditionally known for intracellular roles in signal transduction, protein trafficking, and cell survival. However, extracellular 14-3-3ϵ may function as a damage-associated signal when released into the joint microenvironment.

Evidence suggests that 14-3-3ϵ can stimulate inflammatory responses in synovial cells, macrophages, and chondrocytes (158). It may induce production of cytokines, chemokines, and matrix-remodeling mediators, thereby contributing to synovial inflammation and cartilage catabolism. Its ability to affect both immune cells and chondrocytes makes it particularly relevant to immune–chondrocyte communication. Rather than acting as a classical cytokine, extracellular 14-3-3ϵ appears to function as an alarmin that signals tissue stress and promotes innate immune activation in OA (114). Earlier studies identified soluble 14-3-3ϵ as a mediator released from mechanically stressed subchondral bone cells that promotes catabolic responses in chondrocytes, including increased MMP-3 and MMP-13 expression (159). Mechanistically, CD13 has been identified as a binding partner for extracellular 14-3-3ϵ on chondrocytes, and blockade or knockdown of CD13 attenuates 14-3-3ϵ–induced MMP expression (160). More recent evidence further indicates that the pro-inflammatory effects of extracellular 14-3-3ϵ involve TLR2 and TLR4, as pharmacological or genetic disruption of these receptors reduces 14-3-3ϵ–induced inflammatory responses (114). Thus, although the relative contribution and cell-type specificity of these receptor pathways require further clarification, the identification of extracellular 14-3-3ϵ as a potential alarmin expands the DAMP repertoire in OA beyond the more commonly discussed HMGB1, S100 proteins, and matrix fragments (114). Extracellular 14-3-3ϵ can induce inflammatory phenotypes in synoviocytes, macrophages, and chondrocytes, including increased production of IL-6, CCL2/MCP-1, and TNF-α, thereby providing a mechanistic link between tissue stress and innate immune activation within the OA joint (114). Together with the observation that mechanically stressed subchondral bone releases soluble 14-3-3ϵ capable of promoting chondrocyte catabolism (159), these findings support the concept that intracellular proteins released or secreted under stress conditions may acquire previously underappreciated extracellular signaling functions in joint inflammation. The inclusion of 14-3-3ϵ also illustrates that the DAMP repertoire in OA is likely broader than currently appreciated. Advances in proteomics, spatial profiling, and functional screening may identify additional damage-associated mediators that participate in persistent joint inflammation. Such molecules could help explain how inflammatory activity is maintained in subsets of OA even when classical cytokine signaling is not strongly elevated.

DAMP–PRR signaling provides a unifying mechanism by which cartilage injury, matrix degradation, and cell stress are converted into innate immune activation. HMGB1, S100A8/A9, ECM fragments, low-molecular-weight hyaluronan, tenascin-C, biglycan, decorin, and emerging alarmins such as 14-3-3ϵ serve as endogenous ligands that engage TLRs, RAGE, CD44, integrins, and inflammasome-related pathways. These axes activate NF-κB, MAPK, oxidative stress, and inflammatory cell death programs in chondrocytes and synovial cells.

The key concept is that OA cartilage damage is not silent. Damaged matrix and stressed cells generate molecular signals that activate immune cells, chondrocytes, and other joint-resident cells, thereby amplifying inflammation. In this way, DAMP–PRR axes transform structural cartilage damage into inflammatory information and establish feed-forward loops that sustain matrix degradation, synovitis, chondrocyte dysfunction, and disease progression. DAMP–PRR signaling explains how structural cartilage injury becomes inflammatory information within the OA joint. As shown in **Figure 4**, stressed chondrocytes and fragmented ECM release endogenous ligands that activate innate immune receptors, thereby amplifying synovitis, catabolism, inflammatory cell death, and matrix degradation.

**Figure 4:**
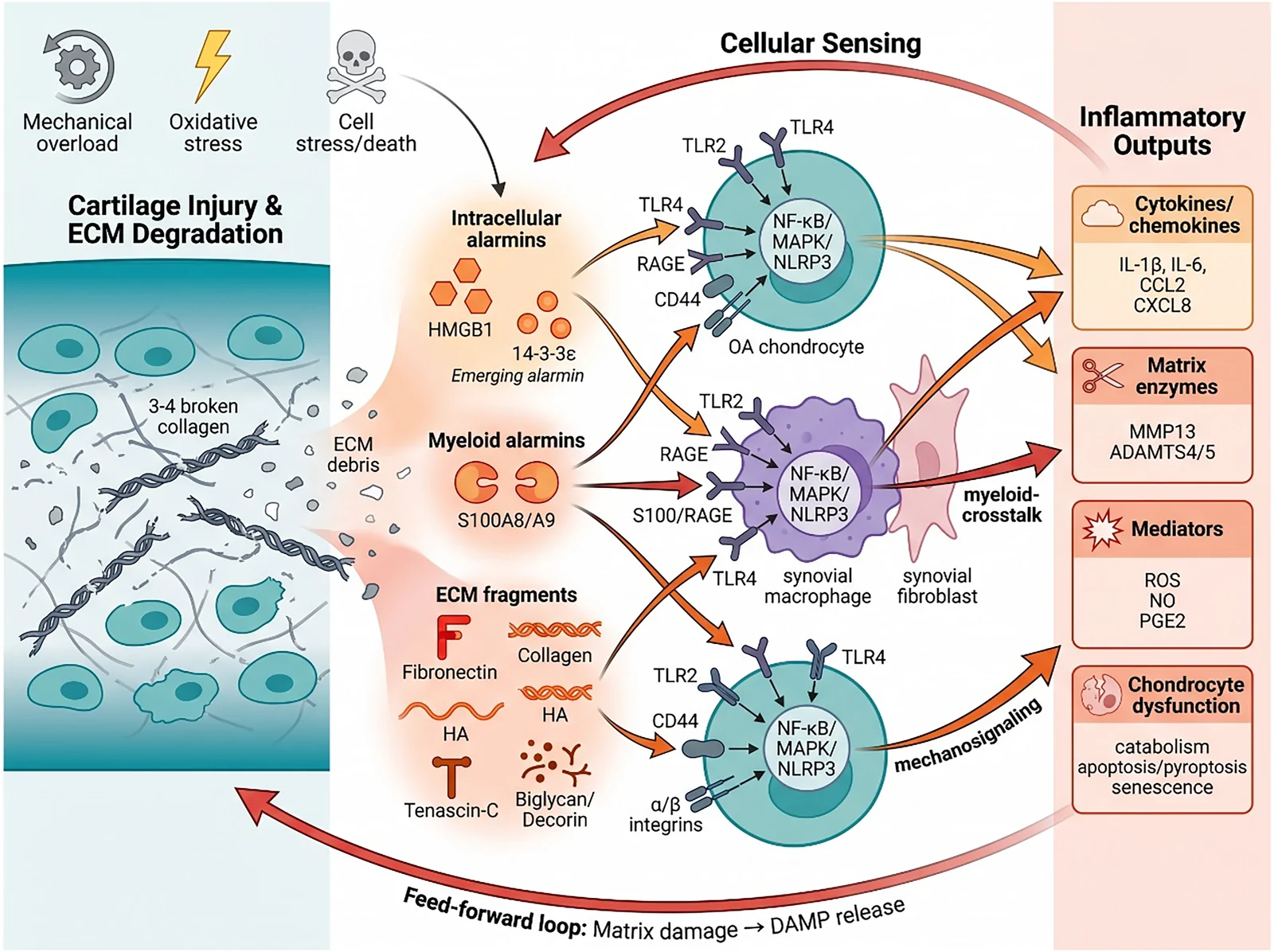
Damage-associated signaling links cartilage injury to innate inflammatory responses in osteoarthritis. Cartilage damage, chondrocyte stress, and extracellular matrix degradation generate DAMPs, including HMGB1, S100A8/A9, matrix fragments, and 14-3-3ϵ. These ligands engage pattern-recognition receptors such as TLR2, TLR4, and RAGE, together with matrix-sensing receptors including CD44 and integrins, promoting inflammatory signaling, protease production, oxidative stress, and further matrix damage.

## 6 Matrix–chondrocyte signaling and secondary inflammatory amplification

ECM-associated ligand–receptor axes represent a critical interface between mechanical stress, matrix remodeling, and immune inflammation in OA (161). Unlike soluble cytokines or chemokines, ECM-associated ligands are embedded within the structural environment of cartilage and periarticular tissues (24). When the matrix is remodeled, fragmented, stiffened, or mechanically overloaded, these ligands become exposed, released, or structurally altered (162). Chondrocytes then sense these changes through adhesion receptors and matrix-binding receptors, including CD44, integrins, syndecans, and other mechanosensitive receptor systems (163, 164). This layer of communication is particularly important because OA is strongly influenced by biomechanics (165). Mechanical overload, abnormal joint alignment, trauma, obesity-associated loading, and altered subchondral bone stiffness can all change the ECM environment surrounding chondrocytes (166, 167). In response, chondrocytes convert mechanical and matrix-derived cues into intracellular signals that regulate inflammation, matrix turnover, survival, hypertrophy, senescence, and death (3, 168). Therefore, ECM-associated ligand–receptor axes do not merely reflect cartilage damage; they actively instruct chondrocytes to adopt mechanoinflammatory and matrix-remodeling phenotypes (111, 169).

### 6.1 OPN/SPP1–CD44/integrin axis

Osteopontin, also known as secreted phosphoprotein 1 (OPN/SPP1), is an ECM-associated cytokine-like ligand that has attracted increasing attention in OA. It can be produced by multiple joint-resident and immune-associated cell types, including macrophages, synovial fibroblasts, chondrocytes, osteoblasts, osteoclasts, and other cells within the osteochondral unit. This broad cellular origin allows OPN/SPP1 to function as a communication molecule linking immune activation, matrix remodeling, subchondral bone changes, and chondrocyte responses (170, 171). OPN/SPP1 can signal through receptors such as CD44 and several integrins. Through these receptors, it can influence cell adhesion, migration, survival, inflammatory activation, and matrix remodeling. In chondrocytes, the OPN/CD44/PI3K axis has been implicated in regulation of OA progression (172). This suggests that OPN/SPP1 is not simply a structural ECM molecule but a ligand capable of activating receptor-dependent intracellular signaling programs. Depending on receptor usage and cellular context, OPN/SPP1 may activate PI3K–AKT, adhesion-related pathways, integrin-associated signaling, and downstream survival or inflammatory responses.

OPN/SPP1 can regulate chondrocyte behavior through multiple receptors and downstream pathways. In OA chondrocytes, OPN binding to CD44 has been linked to PI3K signaling and reduced cartilage matrix loss (172), whereas OPN interaction with CD44 and αvβ3 integrin can modulate ERK/MAPK signaling, autophagy, and chondrocyte proliferation (173). OPN–αvβ3 integrin signaling has also been shown to promote hyaluronic acid synthesis in OA chondrocytes (174). Depending on the cellular and disease context, these signals may therefore influence chondrocyte survival, ECM remodeling, inflammatory responses, and repair-associated processes (172, 173, 175).

A key point is that OPN/SPP1 appears to exert context-dependent effects in OA. Experimental studies have reported potentially protective functions, including suppression of cartilage degeneration, promotion of matrix anabolism, and inhibition of ADAMTS4-associated aggrecan degradation (172, 175–177). Conversely, other studies indicate that increased OPN can promote MMP13 expression through NF-κB signaling and accelerate subchondral bone turnover, type-H vessel formation, and cartilage degeneration (178, 179). Thus, the biological effects of OPN/SPP1 may vary according to tissue compartment, disease stage, receptor context, and local inflammatory conditions. Rather than being uniformly protective or pathogenic, OPN/SPP1 is therefore better viewed as a context-dependent regulator of cartilage and osteochondral remodeling.

OPN/SPP1 is also relevant to macrophage–chondrocyte communication. In OA-related inflammatory models, OPN has been implicated in macrophage-mediated inflammatory signaling and chondrocyte injury (180). More recent studies further suggest that SPP1-associated macrophage states participate in macrophage–chondrocyte crosstalk and may influence cartilage metabolic balance during OA progression (181). Because OPN/SPP1 can interact with CD44 and integrin receptors expressed by chondrocytes and stromal cells (172, 173), SPP1-associated signaling may provide a molecular link between immune-cell activity, ECM remodeling, and chondrocyte phenotype regulation. Importantly, ECM-associated ligand–receptor axes are not necessarily uniformly destructive; their effects may differ according to receptor usage, cellular source, tissue compartment, and disease stage. This principle is particularly relevant to OPN/SPP1, for which both cartilage-protective and disease-promoting effects have been experimentally reported (176, 178, 179). A balanced interpretation is therefore warranted: OPN/SPP1 should be considered a context-dependent matrix-associated regulator of immune–chondrocyte and osteochondral communication rather than a uniformly protective or pathogenic mediator.

### 6.2 Hyaluronan–CD44 as a matrix-derived inflammatory amplification axis

Hyaluronan (HA) is an ECM component rather than an immune cell–derived ligand; therefore, the HA–CD44 axis should not be considered a direct immune cell–chondrocyte communication pathway. We nevertheless include this axis as a matrix-derived secondary amplification mechanism because disruption of HA–CD44 interactions can profoundly alter cartilage matrix homeostasis, whereas HA degradation is enhanced in human OA cartilage (182–184). Notably, TNF-α can promote HA degradation in OA chondrocytes by inducing the HA-depolymerizing protein HYBID, providing a direct link between inflammatory signaling and matrix-derived HA remodeling (184). CD44 is expressed by articular chondrocytes and functions as an important matrix-sensing receptor through which changes in HA integrity can modify chondrocyte behavior (144, 182). In this context, the relevance of HA–CD44 signaling to immune–chondrocyte communication lies primarily in its interaction with inflammation-associated matrix damage. Intact or higher-molecular-weight HA can support chondrocyte survival and suppress catabolic responses through CD44-dependent mechanisms (144, 145)whereas low-molecular-weight HA and HA oligosaccharides can acquire DAMP-like activity. Experimental studies have shown that low-molecular-weight HA activates TLR2/TLR4–MyD88-dependent procatabolic signaling in articular chondrocytes (143), while small HA oligosaccharides activate CD44 and TLR4 in human articular chondrocytes, inducing NF-κB activation and increased production of IL-1β, IL-6, and TNF-α (110). HA fragments can also induce nitric oxide production and matrix-disruptive responses in articular chondrocytes (183, 185). Thus, HA–CD44 is included here not as a direct immune cell–chondrocyte axis, but as a matrix–chondrocyte pathway through which inflammation-associated matrix damage can generate secondary signals that reinforce inflammatory and catabolic responses in OA.

### 6.3 Integrin–ECM axes and mechanotransduction

Integrins are central receptors through which chondrocytes sense ECM composition, stiffness, and mechanical loading. Articular chondrocytes are embedded within a dense ECM, and their phenotype is strongly shaped by signals transmitted through the pericellular and territorial matrix. In OA, matrix degradation, altered collagen organization, fibronectin accumulation, abnormal mechanical stress, and subchondral bone stiffening all change integrin-mediated signaling (155, 186–188). These changes convert ECM remodeling into intracellular mechanotransduction pathways that promote inflammation and catabolism. Several integrin–ECM interactions are relevant to OA, including α5β1 integrin binding to fibronectin, αVβ3 integrin interactions with RGD-containing ligands, collagen–integrin interactions, and other matrix-associated adhesion events. Fibronectin fragments are particularly important because they can stimulate integrin-associated signaling while also activating TLR-related pathways. This dual receptor engagement links matrix damage to both mechanotransduction and innate immune activation.

Upon integrin engagement, chondrocytes activate focal adhesion kinase, Src family kinases, MAPK pathways, PI3K–AKT signaling, and cytoskeletal remodeling. These events can interact with NF-κB and AP-1 transcriptional programs, resulting in increased production of MMPs, ADAMTS enzymes, cytokines, chemokines, and inflammatory mediators. Integrin signaling can also influence YAP/TAZ activity, which connects mechanical cues to transcriptional regulation of cell survival, proliferation, hypertrophy, and matrix remodeling.

ECM remodeling and mechanical overload alter integrin-mediated signaling in chondrocytes, leading to activation of FAK/Src, MAPK, and YAP–TAZ pathways and promoting a mechanoinflammatory phenotype. Integrin–ECM signaling is particularly important in OA because it provides a molecular link between abnormal mechanical loading and inflammatory responses. Rather than acting as a soluble inflammatory mediator, mechanical overload modifies the physical organization and biomechanical properties of the ECM, thereby changing the signals transmitted to chondrocytes through matrix-sensing receptors. Chondrocytes sense these changes through integrins and mechanosensitive pathways. Mechanosensitive ion channels provide an additional interface between the altered biomechanical microenvironment and chondrocyte phenotype. PIEZO1 and PIEZO2 are expressed in articular chondrocytes and act cooperatively to mediate cellular responses to high-strain mechanical loading, including mechanically induced Ca2+ influx (189). Importantly, mechanosensitivity itself can be remodeled during OA. In primary chondrocytes, IL-1α increases PIEZO1 expression through p38 MAPK-dependent inflammatory signaling, whereas PIEZO1 expression is elevated in human OA cartilage (190). Enhanced PIEZO1 activity increases intracellular Ca2+ and sensitizes chondrocytes to mechanically induced cellular injury, thereby establishing a feed-forward interaction between inflammatory signaling and abnormal mechanotransduction (190). Thus, OA-associated changes in cartilage mechanics and mechanosensitive ion-channel activity may cooperate with cytokine and damage-associated signaling to promote chondrocyte phenotypic reprogramming. When the ECM is intact and appropriately loaded, integrin signaling may support survival and matrix homeostasis. When the ECM is fragmented, stiffened, or abnormally loaded, integrin signaling can shift toward catabolic and inflammatory outputs. This axis also interacts with cytokine and DAMP pathways. IL-1β and TNF-α can alter integrin expression and sensitize chondrocytes to matrix fragments. Fibronectin fragments can activate both integrins and TLRs, producing stronger inflammatory responses than either pathway alone. ECM degradation generates new ligands for integrins, CD44, and PRRs, while integrin activation promotes matrix-degrading enzyme production, creating a feed-forward loop of mechanoinflammation. Therefore, integrin–ECM axes are essential for integrating biomechanical stress with immune activation and matrix remodeling (191–195). They help position OA as a disease in which mechanical and inflammatory signals are inseparable. From a therapeutic perspective, targeting integrin-associated signaling, FAK/Src activation, YAP/TAZ dysregulation, or matrix fragment–receptor interactions may offer strategies to interrupt mechanoinflammatory chondrocyte reprogramming.

ECM-associated and adhesion receptor axes provide a key mechanistic bridge between cartilage structure, mechanical stress, and immune inflammation. OPN/SPP1–CD44/integrin signaling links immune and matrix remodeling programs to chondrocyte survival, inflammation, or repair. The HA–CD44 axis illustrates how the same matrix molecule can be protective when intact and inflammatory when fragmented. Integrin–ECM interactions explain how altered matrix composition and mechanical overload are translated into FAK/Src/MAPK/YAP–TAZ-dependent mechanoinflammatory signaling.

Together, these axes show that the ECM is not a passive scaffold in OA. It is a dynamic signaling platform that provides ligands, mechanical cues, and adhesion-dependent instructions to chondrocytes. ECM-associated ligand–receptor axes may be destructive, protective, or compensatory depending on disease stage, molecular form, receptor context, and cellular state. This context dependence is essential for understanding matrix remodeling and for designing therapies that preserve reparative ECM signaling while blocking pathological mechanoinflammation. ECM-associated ligand–receptor axes provide a mechanistic bridge between matrix structure, mechanical stress, and inflammatory chondrocyte reprogramming. As shown in **Figure 5**, intact ECM signals can support cartilage homeostasis, whereas fragmented or stiffened ECM activates CD44-, TLR-, and integrin-dependent mechanoinflammatory pathways that promote matrix remodeling and OA progression.

**Figure 5:**
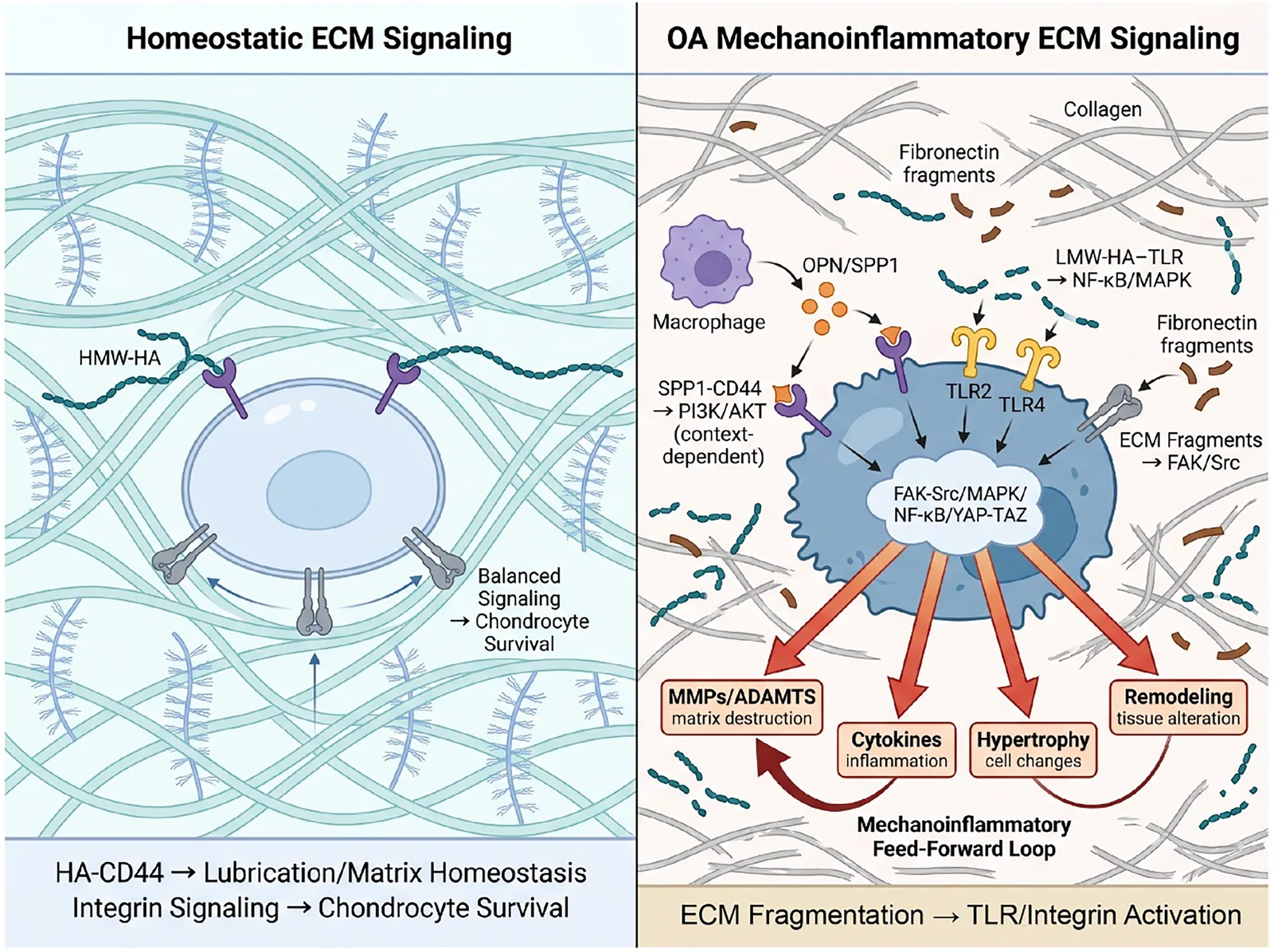
ECM-associated receptor signaling in cartilage homeostasis and osteoarthritis-associated mechanoinflammation. In relatively intact cartilage, high-molecular-weight hyaluronan and organized extracellular matrix support homeostatic CD44- and integrin-mediated signaling. During OA, matrix fragmentation, low-molecular-weight hyaluronan, OPN/SPP1, and altered matrix mechanics modify CD44, TLR, and integrin signaling and activate FAK/Src, MAPK, NF-κB, and YAP/TAZ pathways, thereby contributing to inflammatory and catabolic chondrocyte responses and abnormal matrix remodeling.

## 7 Convergence of immune–chondrocyte signaling on chondrocyte fate

The ligand–receptor axes discussed above converge on a limited number of maladaptive chondrocyte states rather than producing completely independent biological outcomes. Cytokine, chemokine, DAMP-associated, stromal, and matrix-derived signals share downstream nodes such as NF-κB, MAPK/AP-1, JAK/STAT, oxidative-stress pathways, and cell-death machinery (196–200). Their biological effects therefore depend on the intensity, duration, and combination of microenvironmental signals rather than on a single ligand–receptor pair.

Catabolic activation represents a major convergent outcome of inflammatory signaling. IL-1β–IL-1R, TNF-α–TNFR, IL-17–IL-17R, IL-6–IL-6R/gp130, DAMP–PRR, and selected chemokine pathways converge on inflammatory transcriptional programs that increase MMPs and ADAMTS enzymes while suppressing cartilage matrix synthesis (85, 196, 201). This shift promotes progressive loss of collagen and aggrecan, with ADAMTS4/5-mediated aggrecan degradation representing an important component of cartilage matrix breakdown (202). Matrix degradation can in turn generate additional bioactive fragments and damage-associated signals, thereby reinforcing inflammatory communication.

Hypertrophic differentiation and senescence represent related but distinct forms of phenotypic reprogramming. Persistent inflammatory, osteochondral, and mechanical signaling can promote RUNX2-, COL10A1-, MMP13-, and VEGF-associated hypertrophic remodeling, linking cartilage degeneration with calcification, vascular remodeling, and osteochondral changes (197, 203, 204). In parallel, chronic inflammatory signaling and cellular stress can promote p16/p21-associated senescence and development of a senescence-associated secretory phenotype. Experimental studies have linked IL-17–driven immune responses and IL-6R/JAK2 signaling to senescence-associated chondrocyte states in OA (198, 205). Importantly, clearance of senescent cells attenuates post-traumatic OA and reduces the pathological influence of the senescent-cell secretory program (206). Senescent chondrocytes can therefore reinforce joint inflammation through the release of cytokines, chemokines, and matrix-remodeling mediators.

Chondrocyte death and loss of cellular resilience form another convergent endpoint. Increased chondrocyte apoptosis has been demonstrated in human OA cartilage (207), whereas inflammasome-associated pyroptosis and RIPK1/RIPK3/MLKL-related necroptosis provide inflammatory forms of chondrocyte death that can further amplify cartilage injury (208–210). Oxidative and redox dysregulation can additionally increase susceptibility to ferroptosis; experimental OA models show that IL-1β can induce lipid peroxidation, iron accumulation, mitochondrial abnormalities, and suppression of SLC7A11/GPX4 in chondrocytes (199). Autophagy represents an important counter-regulatory mechanism, and reduced autophagic activity in aging and OA cartilage is associated with increased chondrocyte dysfunction and cell death (200, 211). Thus, apoptosis, pyroptosis, necroptosis, ferroptosis, and impaired cellular quality control should be viewed as interconnected downstream consequences of persistent microenvironmental stress rather than as independent ligand–receptor pathways.

Taken together, the principal immune–chondrocyte communication axes converge on four major disease-associated outcomes: catabolic activation, hypertrophic remodeling, senescence, and cell death/loss of stress resilience. These outcomes are interconnected: matrix catabolism generates additional damage-associated signals, hypertrophic remodeling alters the osteochondral environment, senescent cells reinforce inflammation through SASP production, and lytic cell death can release intracellular alarmins that further stimulate inflammatory signaling (206, 209). Thus, Section 7 provides an integrative view of how diverse upstream communication modules discussed in Sections 3–6 are translated into a restricted set of chondrocyte fate programs that collectively drive OA progression. The principal convergent pathways and cellular outcomes are summarized in **Supplementary Table S2**.

## 8 Modes of immune–chondrocyte communication and failure of inflammatory resolution

The communication pathways discussed above should not all be interpreted as direct cell–cell interactions. In OA, immune cells can influence chondrocytes through at least three experimentally distinguishable modes: physical or contact-dependent interaction, paracrine signaling through soluble mediators, and indirect relay signaling through synovial fibroblasts or other joint-resident cells (212–215). Here, we use the term direct cell–cell interaction only when experimental systems permit physical contact between defined immune cells and chondrocytes or when spatial evidence supports close cellular juxtaposition. By contrast, conditioned-medium and transwell experiments are interpreted as evidence of paracrine communication (213, 214), whereas effects requiring an intermediate stromal population are considered indirect relay signaling (215, 216). Importantly, computational ligand–receptor inference or transcript co-expression alone should not be considered proof of direct cell–cell interaction because such analyses do not independently establish physical contact, ligand secretion, receptor activation, or functional signaling (217).

### 8.1 Experimental evidence for direct and paracrine immune–chondrocyte communication

Evidence for T-cell–chondrocyte communication has been obtained using human cell-based co-culture systems. In one study, primary human chondrocytes were co-cultured with activated CD4+ T cells either in direct contact or across a transwell membrane, allowing contact-dependent and soluble-factor-mediated effects to be experimentally distinguished; chondrocyte expression of aggrecan, type II collagen, and MMP13 was altered under these conditions (218). More recently, chondrocytes isolated from patients with knee OA were co-cultured with polarized autologous or patient-derived CD4+ T-cell subsets, with Th17-containing co-cultures showing particularly strong increases in inflammatory cytokines and matrix metalloproteinases (212). These studies support functional T-cell–chondrocyte communication, but soluble cytokine signaling should be distinguished from strictly contact-dependent effects.

Macrophage–chondrocyte communication is supported more strongly by paracrine experimental models. Conditioned medium from human monocyte-derived pro-inflammatory macrophages promotes inflammatory and degenerative responses in human OA cartilage explants, demonstrating that macrophage-secreted factors can alter cartilage homeostasis without direct macrophage–chondrocyte contact (213). Similarly, a three-dimensional transwell co-culture model using human chondrocytes and activated macrophages demonstrated reciprocal inflammatory responses while physically separating the two cell populations, providing evidence for bidirectional paracrine rather than contact-dependent communication (214). Thus, macrophage–chondrocyte crosstalk in OA should primarily be described as a soluble mediator-driven reciprocal circuit unless direct contact has been specifically demonstrated.

### 8.2 Indirect relay communication within the joint microenvironment

Not all immune effects on chondrocytes occur through direct immune–chondrocyte communication (25). Immune-derived cytokines can first activate synovial fibroblasts, adipose-associated cells, endothelial cells, or osteochondral populations, which subsequently release IL-6, chemokines, growth factors, proteases, and other mediators that act on chondrocytes (32, 219). These interactions are more appropriately classified as stromal relay signaling (215). Likewise, chondrocyte-derived chemokines and DAMPs may act initially on macrophages or fibroblasts, which then reshape the inflammatory milieu and indirectly alter cartilage behavior (216). Distinguishing these relay circuits from direct immune–chondrocyte communication is important because ligand expression alone does not establish the cellular route through which a biological effect occurs (217). Spatial transcriptomics and multiplex imaging can complement functional culture systems by determining whether ligand-producing immune populations and receptor-bearing chondrocyte states occupy compatible anatomical niches (220, 221). However, spatial proximity and computational ligand–receptor inference should be considered supportive rather than definitive evidence of direct communication unless accompanied by functional perturbation (222).

### 8.3 Efferocytosis failure and unresolved inflammation

Defective inflammatory resolution represents a distinct mechanism by which immune dysfunction can indirectly sustain chondrocyte injury. Analysis of human knee OA synovial tissue has demonstrated accumulation of apoptotic cells together with impaired efferocytosis by CD14+ synovial macrophages (223). Experimental work in obesity-associated OA further showed that reduced GAS6 availability impaired macrophage efferocytosis, promoted accumulation of apoptotic material and inflammatory mediators, and aggravated cartilage degeneration, whereas restoration of GAS6 improved macrophage clearance capacity and attenuated OA pathology (224). These findings support defective efferocytosis as an experimentally validated macrophage abnormality in OA rather than a direct macrophage–chondrocyte interaction.

Overall, immune–chondrocyte communication in OA comprises contact-dependent interactions, soluble paracrine signaling, and indirect multicellular relay circuits. Explicitly distinguishing these modes prevents evidence from conditioned-medium or transwell experiments from being interpreted as proof of physical cell–cell contact and provides a more rigorous framework for evaluating immune–chondrocyte crosstalk in OA.

## 9 Single-cell and spatial evidence for cellular communication networks in OA

Published single-cell and spatial studies have progressively shifted OA research from descriptive cell-state cataloguing toward the mapping of multicellular and cross-tissue communication networks. Representative OA studies using single-cell sequencing, spatial transcriptomics, and cell–cell communication analyses are summarized in **Supplementary Table S3**, with emphasis on tissue source, major cell states, candidate ligand–receptor modules, and the level of spatial or functional validation.

### 9.1 Human cartilage studies reveal disease-associated chondrocyte states

Single-cell studies have substantially refined the cellular landscape of human OA cartilage. Ji et al. performed scRNA-seq on 1,464 chondrocytes from 10 patients undergoing knee arthroplasty and identified seven molecularly distinct chondrocyte populations, including proliferative, prehypertrophic, hypertrophic, fibrocartilage, regulatory, and cartilage progenitor-associated states (225). This study provided early evidence that OA cartilage comprises transcriptionally distinct chondrocyte states rather than a homogeneous cell population. Single-cell mass cytometry subsequently identified inflammation-amplifying and inflammation-dampening chondrocyte populations in human OA cartilage, further supporting functional heterogeneity within diseased cartilage (226).

More recent studies have linked these cellular states to spatially organized OA pathology. Fan et al. combined single-cell and spatial transcriptomics in human knee cartilage from OA and non-OA donors and identified 11 chondrocyte populations, including previously unrecognized pre-inflammatory and inflammatory chondrocytes (220). Inflammatory chondrocytes were associated with MIF–CD74 signaling, whereas prehypertrophic and hypertrophic chondrocytes emerged as OA-critical populations. Importantly, OA-associated transcriptional changes were enriched at the articular surface and superficial cartilage zones, providing spatial evidence that disease-associated cell states are not uniformly distributed across cartilage (220).

### 9.2 Published evidence for synovium–cartilage and whole-joint communication

Single-cell studies examining multiple joint compartments have provided more direct evidence for communication between synovial and cartilage cell populations. Chou et al. profiled 10,640 synoviocytes and 26,192 chondrocytes from matched human OA tissues and identified 12 synovial cell types and seven chondrocyte phenotypes (25). Among 61 cytokines and growth factors predicted to regulate chondrocyte phenotypes, more than half were expressed by synovial populations, and a substantial proportion were detected exclusively in synoviocytes. IL-1β-producing synovial cells were predominantly inflammatory macrophages and dendritic cells, providing cell-resolved evidence that synovial immune populations constitute important sources of ligands acting on cartilage (25).

The inflammatory network extends beyond the synovium itself. Tang et al. generated single-cell and single-nucleus atlases of human infrapatellar fat pad and synovial tissues and identified a profibrotic and inflammatory mesenchymal program in OA (32). APOE signaling from intermediate fibroblasts and macrophages emerged as a candidate pathogenic communication pathway; soluble APOE accelerated proteoglycan degeneration in human cartilage *ex vivo*, whereas APOE neutralization attenuated experimental OA in mice (32). This study therefore provides an example in which single-cell inference was accompanied by functional validation of a stromal/immune-derived signal affecting cartilage.

A more recent multi-tissue human knee atlas integrating cartilage, meniscus, synovium, and subchondral bone demonstrated expansion of inflammatory IL1B/IL6/NOS2/TNF-expressing macrophages together with MMP13-producing detrimental chondrocytes and RUNX2-associated osteogenic cell states in OA (219). Cell–cell communication analysis further identified altered signaling between chondrocytes, macrophages, fibroblasts, and tissue stem/progenitor populations, supporting the concept that OA communication networks extend across multiple joint compartments (219).

### 9.3 Ligand–receptor and spatial analyses identify specific OA communication modules

Computational ligand–receptor analyses have begun to identify specific communication modules altered in OA. Using publicly available human cartilage scRNA-seq data and CellChat, Kang et al. reported increased overall communication frequency and strength in damaged compared with intact cartilage, with selective alterations in PTN, VISFATIN, SPP1, and TGF-β signaling among chondrocyte subpopulations (227). These findings are relevant to the present review because they indicate that OA progression involves reorganization of communication networks rather than uniform activation of all ligand–receptor pathways.

Other analyses have extended this framework to synovium–cartilage communication. Single-cell profiling of OA synovial fibroblasts identified distinct fibroblast populations with predicted interactions with chondrocytes from damaged and relatively preserved cartilage (216). More recently, integrated single-cell and spatial transcriptomics of human hip OA synovium identified EREG-positive inflammatory lining fibroblasts and spatially supported FGF2–SDC4 communication between COL1A1-positive/IGFBP5-positive fibrotic macrophages and these fibroblasts (221). Although this interaction represents macrophage–fibroblast rather than direct macrophage–chondrocyte signaling, it illustrates how spatially resolved OA studies can distinguish direct immune communication from stromal relay circuits (221).

### 9.4 Evidence limitations

Despite these advances, most OA ligand–receptor networks identified by single-cell analysis remain inferential. Transcript co-expression does not establish ligand secretion, receptor activation, spatial proximity, or downstream signaling, and several studies rely on reanalysis of existing datasets rather than independent experimental cohorts. Moreover, many human cartilage samples are obtained from patients undergoing arthroplasty and therefore predominantly represent advanced disease. Spatial transcriptomics, protein-level validation, co-culture or explant models, and cell-specific perturbation are consequently required to distinguish functional communication pathways from computational associations. Published OA studies combining transcriptomic inference with spatial or functional validation remain comparatively limited, underscoring the need for cautious interpretation of predicted ligand–receptor networks.

## 10 Therapeutic translation of immune–chondrocyte LR axes: clinical evidence, preclinical strategies, and emerging concepts

Therapeutic strategies targeting immune–chondrocyte ligand–receptor (LR) signaling are at markedly different stages of development. To avoid conflating mechanistic promise with clinical efficacy, we distinguish three levels of evidence in this section: clinically tested approaches, which have been evaluated in patients with OA; preclinical strategies, supported by cell, explant, or animal studies but not yet established clinically; and hypothesis-generating concepts, including LR-based endotyping, combination targeting, and biomarker-guided treatment selection that currently lack prospective clinical validation. This distinction is particularly important because strong mechanistic evidence for an inflammatory pathway does not necessarily translate into therapeutic benefit in patients with OA.

### 10.1 Clinically tested inflammatory and ligand–receptor–directed approaches

Clinical trials targeting inflammatory ligand–receptor pathways in OA have generally produced limited or inconsistent efficacy. In a randomized placebo-controlled trial of knee OA, a single intra-articular injection of the IL-1 receptor antagonist anakinra was well tolerated but did not significantly improve WOMAC outcomes compared with placebo (228). Dual blockade of IL-1α and IL-1β with lutikizumab was subsequently evaluated in phase II trials in knee OA with synovitis and erosive hand OA; despite pharmacodynamic evidence of IL-1 inhibition, clinically meaningful symptomatic or structural benefits were limited (229, 230). These studies illustrate that the strong experimental rationale for IL-1 signaling has not translated into consistent clinical efficacy.

Similar limitations have been observed with other cytokine pathways. Tocilizumab, an IL-6 receptor-blocking antibody, failed to improve pain or secondary clinical outcomes in a randomized trial of symptomatic hand OA (231). TNF inhibition has also been clinically tested. Adalimumab did not improve pain or MRI-detected synovitis in erosive hand OA (232), while etanercept did not achieve its primary pain endpoint in inflammatory hand OA, although exploratory structural signals were observed in selected inflamed joints (233). Collectively, these trials indicate that inhibition of a single cytokine or receptor is not an established disease-modifying strategy for unselected OA populations.

### 10.2 Preclinical targeting of receptor modules and signaling nodes

In contrast to the clinically tested cytokine-blocking approaches described above, most strategies targeting receptor-proximal signaling modules remain preclinical. For example, selective modulation of gp130 signaling reduced cartilage loss, synovitis, and fibrosis and enhanced regenerative responses in experimental OA models, supporting gp130 pathway selectivity as a mechanistically attractive but as yet unvalidated clinical strategy (19). Likewise, blockade or genetic disruption of the CCL2–CCR2 axis reduced monocyte/macrophage accumulation, synovitis, and cartilage damage in murine OA, although comparable efficacy has not yet been demonstrated in clinical OA trials (21).

A mechanistic study combining human OA cartilage, a surgically induced rabbit knee OA model established using Hulth’s method, and TNF-α-stimulated C28/I2 human chondrocytes demonstrated increased FUT10-associated α-1,3-fucosylation under OA-related conditions. Mechanistic experiments in C28/I2 chondrocytes further showed that FUT10-dependent α-1,3-fucosylation of TNFR1 enhanced TNF-α binding and activation of NF-κB and p38/JNK-MAPK signaling, whereas FUT10 silencing reduced these responses and attenuated ECM degradation, senescence, and apoptosis (234). CXCL12–CXCR4 inhibition has also shown protective effects in experimental OA models and human OA chondrocyte systems, but these findings should currently be interpreted as preclinical evidence rather than proof of therapeutic efficacy in patients (80, 235).

### 10.3 Preclinical local delivery and macrophage-directed strategies

Local delivery and macrophage-directed approaches are also predominantly at the preclinical stage. For example, IgG-opsonized bilirubin/JPH203 nanoparticles preferentially targeted inflammatory macrophages, promoted macrophage repolarization, and reduced cartilage pathology after intra-articular administration in a rat OA model (236). Similarly, macrophage-targeted rapamycin-loaded nanoparticles reduced GSDMD-associated pyroptosis, enhanced mitophagy, and attenuated cartilage degeneration in a collagen-induced OA mouse model (237). These studies provide proof-of-concept that macrophage states can be therapeutically manipulated within the joint, but neither platform has yet established clinical efficacy in patients with OA. Nanoparticles, hydrogels, extracellular vesicles, nucleic-acid delivery systems, and sustained-release formulations may improve intra-articular retention and cell-specific targeting. However, most such systems remain experimental, and their translation will require demonstration of reproducible efficacy, long-term biocompatibility, scalable manufacturing, and safety in large-animal models and clinical trials. Accordingly, these platforms should be presented as delivery technologies under preclinical development rather than as established OA therapies.

### 10.4 Hypothesis-generating precision strategies

LR-axis-based patient stratification remains a hypothesis-generating concept rather than a clinically validated strategy. In principle, OA could be stratified according to dominant inflammatory, chemokine, DAMP, senescence, or remodeling-associated communication modules. Such molecular endotypes might eventually help identify patients more likely to respond to pathway-specific interventions. However, proposed categories such as “IL-6-high,” “Th17-high,” “DAMP-high,” or “chemokine-high” OA have not yet been prospectively validated as treatment-selection biomarkers. Similarly, composite LR-module scores integrating ligand expression, receptor abundance, downstream pathway activation, and spatial cell-state information remain exploratory. Before these approaches can inform clinical decision making, candidate biomarkers will require analytical validation, replication in independent patient cohorts, association with longitudinal clinical outcomes, and prospective testing as predictive biomarkers in intervention trials. Therefore, LR-based endotyping should currently be viewed as a framework for future precision-OA research rather than an established therapeutic strategy.

## 11 Challenges and future research priorities

Despite rapid progress in defining immune–chondrocyte communication in OA, several limitations currently prevent candidate ligand–receptor (LR) networks from being translated into robust disease mechanisms or therapeutic targets. Future work should prioritize three areas: causal validation of cell-specific communication, resolution of spatial and temporal heterogeneity, and improved translational validation across human OA populations.

### 11.1 From ligand–receptor inference to causal and cell-specific validation

A major challenge is that transcriptomic co-expression of a ligand in one cell population and its receptor in another does not by itself establish functional communication. Single-cell studies have identified extensive predicted signaling between synovial populations and chondrocyte states, while spatial transcriptomic studies have further localized OA-associated chondrocyte populations and candidate signaling modules (25, 220). However, these observations require functional confirmation at the protein and cellular levels. Experimental studies using direct-contact and transwell co-culture of human CD4+ T cells and chondrocytes, as well as macrophage-conditioned medium applied to human OA cartilage, illustrate how communication routes can be distinguished experimentally (213, 218). Particularly informative studies combine cell-resolved discovery with functional perturbation; for example, single-cell analysis of human infrapatellar fat pad and synovium identified APOE-associated signaling, which was subsequently tested in human cartilage ex vivo and in experimental OA *in vivo* (32). Future studies should therefore prioritize cell-source-specific perturbation, spatial confirmation, and functional receptor blockade rather than relying on LR prediction alone.

### 11.2 Resolving spatial, temporal, and clinical heterogeneity

OA communication networks are likely to vary across anatomical compartments, disease stages, and patient phenotypes. Recent spatial analyses demonstrate that OA-associated transcriptional changes and inflammatory chondrocyte states are not uniformly distributed within cartilage (220). Human studies also illustrate the importance of disease stage: analyses of synovial macrophage efferocytosis have included both late-stage arthroplasty tissue and synovial fluid from earlier-stage disease, revealing stage-associated differences in the inflammatory microenvironment (223). Nevertheless, many available human datasets remain cross-sectional and are enriched for surgically obtained tissues. Future studies should therefore incorporate earlier disease stages, longitudinal sampling, multiple joint compartments, and clinically defined OA phenotypes to determine which LR networks initiate disease, which sustain progression, and which reflect late-stage remodeling.

### 11.3 Reproducibility and translational validation

A final priority is to establish communication modules that are reproducible across cohorts and experimental platforms. Published OA single-cell studies differ substantially in the tissues analyzed, including cartilage, synovium, infrapatellar fat pad, and combined joint compartments (25, 32, 220). These studies have substantially expanded the cellular landscape of OA but also highlight the need for harmonized cell-state annotation, standardized tissue processing, and independent cohort validation. Candidate LR axes should ideally be supported by convergent evidence from human single-cell or spatial datasets, protein-level localization, physiologically relevant co-culture or explant systems, and perturbation experiments. Only communication modules that remain consistent across these levels should be prioritized for biomarker development or therapeutic translation.

Collectively, the next phase of OA LR research should move from increasingly large catalogs of predicted interactions toward a smaller number of cell-source-defined, spatially resolved, functionally validated, and clinically reproducible communication pathways. Such an evidence hierarchy will be essential for distinguishing causal drivers of OA progression from secondary or compensatory signaling responses.

## 12 Conclusion

OA is a multifactorial whole-joint disorder involving interacting mechanical, inflammatory, metabolic, stromal, osteochondral, and ECM-associated processes. Within this broader disease context, immune–chondrocyte communication represents one important component of the local microenvironment that can influence cartilage homeostasis and degeneration. Cytokine and chemokine signaling, together with damage-associated and matrix-derived cues, can modify chondrocyte inflammatory activation, matrix catabolism, senescence, hypertrophic remodeling, and cell survival. Importantly, the evidence supporting these communication pathways is not uniform. Some immune cell–chondrocyte interactions are supported by co-culture, conditioned-medium, human tissue, and functional perturbation studies, whereas other proposed pathways involve indirect stromal relay, chondrocyte autocrine signaling, or matrix-derived ligands rather than direct immune cell–chondrocyte communication. In addition, several ligand–receptor networks identified by single-cell or spatial analyses remain computationally inferred and require protein-level, spatial, and functional validation. Many therapeutic strategies targeting these pathways also remain predominantly preclinical. Future studies should therefore prioritize cell-source-specific validation, spatial and temporal resolution, physiologically relevant experimental models, and confirmation in well-characterized human OA cohorts. Defining which communication modules are causal, context-dependent, and therapeutically tractable may improve understanding of OA heterogeneity and help identify more precisely targeted interventions within the broader framework of whole-joint disease. Future progress in this field will depend less on expanding catalogs of predicted ligand–receptor pairs and more on identifying a smaller number of communication pathways that are reproducible, spatially resolved, functionally validated, and clinically relevant. Integrating human OA tissues with single-cell and spatial profiling, protein-level localization, physiologically relevant co-culture or explant systems, and cell-specific perturbation will be essential for distinguishing causal signaling axes from associative or context-dependent interactions. Equally important will be determining how these pathways vary across disease stage, anatomical compartment, and patient phenotype. Such an evidence-based framework may ultimately support more precise therapeutic targeting while avoiding oversimplification of OA as a single inflammatory or immune-driven disease. In this context, immune–chondrocyte communication should be viewed not as an isolated mechanism but as a dynamic component of the broader multicellular and biomechanical network that governs whole-joint homeostasis and degeneration.

## References

[1] Loeser RF Goldring SR Scanzello CR Goldring MB . Osteoarthritis: a disease of the joint as an organ. Arthritis Rheumatism. (2012) 64:1697–707. doi: 10.1002/art.34453 22392533 PMC3366018

[2] Poole AR . Osteoarthritis as a whole joint disease. HSS Journal: Musculoskeletal J Hosp For Special Surg. (2012) 8:4–6. doi: 10.1007/s11420-011-9248-6 23372516 PMC3295952

[3] Fang T Zhou X Jin M Nie J Li X . Molecular mechanisms of mechanical load-induced osteoarthritis. Int Orthopaedics. (2021) 45:1125–36. doi: 10.1007/s00264-021-04938-1 33459826

[4] Chow YY Chin KY . The role of inflammation in the pathogenesis of osteoarthritis. Mediators Inflammation. (2020) 2020:8293921. doi: 10.1155/2020/8293921 32189997 PMC7072120

[5] Lambert C Zappia J Sanchez C Florin A Dubuc JE Henrotin Y . The damage-associated molecular patterns (DAMPs) as potential targets to treat osteoarthritis: perspectives from a review of the literature. Front Med. (2020) 7:607186. doi: 10.3389/fmed.2020.607186 33537330 PMC7847938

[6] Zheng L Zhang Z Sheng P Mobasheri A . The role of metabolism in chondrocyte dysfunction and the progression of osteoarthritis. Ageing Res Rev. (2021) 66:101249. doi: 10.1016/j.arr.2020.101249 33383189

[7] Mathiessen A Conaghan PG . Synovitis in osteoarthritis: current understanding with therapeutic implications. Arthritis Res Ther. (2017) 19:18. doi: 10.1186/s13075-017-1229-9 28148295 PMC5289060

[8] Ramezanpour S Kanthawang T Lynch J McCulloch CE Nevitt MC Link TM . Impact of sustained synovitis on knee joint structural degeneration: 4-year MRI data from the osteoarthritis initiative. J Magnetic Resonance Imaging: JMRI. (2023) 57:153–64. doi: 10.1002/jmri.28223 35561016 PMC9653513

[9] de Lange-Brokaar BJ Ioan-Facsinay A van Osch GJ Zuurmond AM Schoones J Toes RE . Synovial inflammation, immune cells and their cytokines in osteoarthritis: a review. Osteoarthritis Cartilage. (2012) 20:1484–99. doi: 10.1016/j.joca.2012.08.027 22960092

[10] Sofat N . Analysing the role of endogenous matrix molecules in the development of osteoarthritis. Int J Exp Path. (2009) 90:463–79. doi: 10.1111/j.1365-2613.2009.00676.x 19765101 PMC2768145

[11] Sokolove J Lepus CM . Role of inflammation in the pathogenesis of osteoarthritis: latest findings and interpretations. Ther Adv Musculoskeletal Dis. (2013) 5:77–94. doi: 10.1177/1759720x12467868 23641259 PMC3638313

[12] Fernandes JC Martel-Pelletier J Pelletier JP . The role of cytokines in osteoarthritis pathophysiology. Biorheology. (2002) 39:237–46. doi: 10.1177/0006355x2002039001002025 12082286

[13] Jenei-Lanzl Z Meurer A Zaucke F . Interleukin-1β signaling in osteoarthritis - chondrocytes in focus. Cell Signalling. (2019) 53:212–23. doi: 10.1016/j.cellsig.2018.10.005 30312659

[14] Wiegertjes R van de Loo FAJ Blaney Davidson EN . A roadmap to target interleukin-6 in osteoarthritis. Rheumatol (Oxford England). (2020) 59:2681–94. doi: 10.1093/rheumatology/keaa248 32691066 PMC7516110

[15] Honorati MC Bovara M Cattini L Piacentini A Facchini A . Contribution of interleukin 17 to human cartilage degradation and synovial inflammation in osteoarthritis. Osteoarthritis Cartilage. (2002) 10:799–807. doi: 10.1053/joca.2002.0829 12359166

[16] Rosenberg JH Rai V Dilisio MF Agrawal DK . Damage-associated molecular patterns in the pathogenesis of osteoarthritis: potentially novel therapeutic targets. Mol Cell Biochem. (2017) 434:171–9. doi: 10.1007/s11010-017-3047-4 28474284 PMC5671379

[17] Martel-Pelletier J McCollum R DiBattista J Faure MP Chin JA Fournier S . The interleukin-1 receptor in normal and osteoarthritic human articular chondrocytes. Identification as the type I receptor and analysis of binding kinetics and biologic function. Arthritis Rheumatism. (1992) 35:530–40. doi: 10.1002/art.1780350507 1533521

[18] Webb GR Westacott CI Elson CJ . Osteoarthritic synovial fluid and synovium supernatants up-regulate tumor necrosis factor receptors on human articular chondrocytes. Osteoarthritis Cartilage. (1998) 6:167–76. doi: 10.1053/joca.1998.0109 9682783

[19] Shkhyan R Flynn C Lamoure E Sarkar A Van Handel B Li J . Inhibition of a signaling modality within the GP130 receptor enhances tissue regeneration and mitigates osteoarthritis. Sci Transl Med. (2023) 15:eabq2395. doi: 10.1126/scitranslmed.abq2395 36947594 PMC10792550

[20] Lu W Shi J Zhang J Lv Z Guo F Huang H . CXCL12/CXCR4 axis regulates aggrecanase activation and cartilage degradation in a post-traumatic osteoarthritis rat model. Int J Mol Sci. (2016) 17. doi: 10.3390/ijms17101522 27690009 PMC5085615

[21] Raghu H Lepus CM Wang Q Wong HH Lingampalli N Oliviero F . CCL2/CCR2, but not CCL5/CCR5, mediates monocyte recruitment, inflammation and cartilage destruction in osteoarthritis. Ann Rheumatic Dis. (2017) 76:914–22. doi: 10.1136/annrheumdis-2016-210426 27965260 PMC5834918

[22] Loeser RF Yammani RR Carlson CS Chen H Cole A Im HJ . Articular chondrocytes express the receptor for advanced glycation end products: potential role in osteoarthritis. Arthritis Rheumatism. (2005) 52:2376–85. doi: 10.1002/art.21199 16052547 PMC1488730

[23] Sun XH Liu Y Han Y Wang J . Expression and significance of high-mobility group protein B1 (HMGB1) and the receptor for advanced glycation end-product (RAGE) in knee osteoarthritis. Med Sci Monitor: Int Med J Exp Clin Res. (2016) 22:2105–12. doi: 10.12659/msm.895689 27320800 PMC4918532

[24] Knudson W Loeser RF . CD44 and integrin matrix receptors participate in cartilage homeostasis. Cell Mol Life Sciences: CMLS. (2002) 59:36–44. doi: 10.1007/s00018-002-8403-0 11846031 PMC11337496

[25] Chou CH Jain V Gibson J Attarian DE Haraden CA Yohn CB . Synovial cell cross-talk with cartilage plays a major role in the pathogenesis of osteoarthritis. Sci Rep. (2020) 10:10868. doi: 10.1038/s41598-020-67730-y 32616761 PMC7331607

[26] Knights AJ Farrell EC Ellis OM Lammlin L Junginger LM Rzeczycki PM . Synovial fibroblasts assume distinct functional identities and secrete R-spondin 2 in osteoarthritis. Ann Rheumatic Dis. (2023) 82:272–82. doi: 10.1136/ard-2022-222773 36175067 PMC9972892

[27] Towle CA Hung HH Bonassar LJ Treadwell BV Mangham DC . Detection of interleukin-1 in the cartilage of patients with osteoarthritis: a possible autocrine/paracrine role in pathogenesis. Osteoarthritis Cartilage. (1997) 5:293–300. doi: 10.1016/s1063-4584(97)80008-8 9497936

[28] Yuan GH Masuko-Hongo K Sakata M Tsuruha J Onuma H Nakamura H . The role of C-C chemokines and their receptors in osteoarthritis. Arthritis Rheumatism. (2001) 44:1056–70. doi: 10.1002/1529-0131(200105)44:5<1056::AID-ANR186>3.0.CO;2-U 11352237

[29] Alaaeddine N Antoniou J Moussa M Hilal G Kreichaty G Ghanem I . The chemokine CCL20 induces proinflammatory and matrix degradative responses in cartilage. Inflammation Research: Off J Eur Histamine Res Soc [et al]. (2015) 64:721–31. doi: 10.1007/s00011-015-0854-5 26189947

[30] Salter DM Millward-Sadler SJ Nuki G Wright MO . Integrin-interleukin-4 mechanotransduction pathways in human chondrocytes. Clin Orthopaedics Related Res. (2001), S49–60. doi: 10.1097/00003086-200110001-00006 11603724

[31] Huang ZY Luo ZY Cai YR Chou CH Yao ML Pei FX . Single cell transcriptomics in human osteoarthritis synovium and in silico deconvoluted bulk RNA sequencing. Osteoarthritis Cartilage. (2022) 30:475–80. doi: 10.1016/j.joca.2021.12.007 34971754 PMC10097426

[32] Tang S Yao L Ruan J Kang J Cao Y Nie X . Single-cell atlas of human infrapatellar fat pad and synovium implicates APOE signaling in osteoarthritis pathology. Sci Transl Med. (2024) 16:eadf4590. doi: 10.1126/scitranslmed.adf4590 38266107

[33] Chen B Sun Y Xu G Jiang J Zhang W Wu C . Role of crosstalk between synovial cells and chondrocytes in osteoarthritis (review). Exp Ther Med. (2024) 27:201. doi: 10.3892/etm.2024.12490 38590580 PMC11000048

[34] Pearson MJ Herndler-Brandstetter D Tariq MA Nicholson TA Philp AM Smith HL . IL-6 secretion in osteoarthritis patients is mediated by chondrocyte-synovial fibroblast cross-talk and is enhanced by obesity. Sci Rep. (2017) 7:3451. doi: 10.1038/s41598-017-03759-w 28615667 PMC5471184

[35] Wood MJ Leckenby A Reynolds G Spiering R Pratt AG Rankin KS . Macrophage proliferation distinguishes 2 subgroups of knee osteoarthritis patients. JCI Insight. (2019) 4. doi: 10.1172/jci.insight.125325 30674730 PMC6413777

[36] Shen PC Wu CL Jou IM Lee CH Juan HY Lee PJ . T helper cells promote disease progression of osteoarthritis by inducing macrophage inflammatory protein-1γ. Osteoarthritis Cartilage. (2011) 19:728–36. doi: 10.1016/j.joca.2011.02.014 21376128

[37] Keller LE Tait Wojno ED Begum L Fortier LA . T helper 17-like regulatory T cells in equine synovial fluid are associated with disease severity of naturally occurring posttraumatic osteoarthritis. Am J Sports Med. (2023) 51:1047–58. doi: 10.1177/03635465231153588 36794851 PMC10375548

[38] de Lange-Brokaar BJ Kloppenburg M Andersen SN Dorjée AL Yusuf E Herb-van Toorn L . Characterization of synovial mast cells in knee osteoarthritis: association with clinical parameters. Osteoarthritis Cartilage. (2016) 24:664–71. doi: 10.1016/j.joca.2015.11.011 26671522

[39] Farinelli L Aquili A Mattioli-Belmonte M Manzotti S D'Angelo F Ciccullo C . Synovial mast cells from knee and hip osteoarthritis: histological study and clinical correlations. J Exp Orthopaedics. (2022) 9:13. doi: 10.1186/s40634-022-00446-2 35079910 PMC8789998

[40] Kriegova E Manukyan G Mikulkova Z Gabcova G Kudelka M Gajdos P . Gender-related differences observed among immune cells in synovial fluid in knee osteoarthritis. Osteoarthritis Cartilage. (2018) 26:1247–56. doi: 10.1016/j.joca.2018.04.016 29753948

[41] Manukyan G Gallo J Mikulkova Z Trajerova M Savara J Slobodova Z . Phenotypic and functional characterisation of synovial fluid-derived neutrophils in knee osteoarthritis and knee infection. Osteoarthritis Cartilage. (2023) 31:72–82. doi: 10.1016/j.joca.2022.09.011 36216277

[42] Haraden CA Huebner JL Hsueh MF Li YJ Kraus VB . Synovial fluid biomarkers associated with osteoarthritis severity reflect macrophage and neutrophil related inflammation. Arthritis Res Ther. (2019) 21:146. doi: 10.1186/s13075-019-1923-x 31196179 PMC6567574

[43] Nanus DE Badoume A Wijesinghe SN Halsey AM Hurley P Ahmed Z . Synovial tissue from sites of joint pain in knee osteoarthritis patients exhibits a differential phenotype with distinct fibroblast subsets. EBioMedicine. (2021) 72:103618. doi: 10.1016/j.ebiom.2021.103618 34628351 PMC8511845

[44] Zhou X Cao H Yuan Y Wu W . Biochemical signals mediate the crosstalk between cartilage and bone in osteoarthritis. BioMed Res Int. (2020) 2020:5720360. doi: 10.1155/2020/5720360 32337258 PMC7165323

[45] Jiang A Xu P Sun S Zhao Z Tan Q Li W . Cellular alterations and crosstalk in the osteochondral joint in osteoarthritis and promising therapeutic strategies. Connective Tissue Res. (2021) 62:709–19. doi: 10.1080/03008207.2020.1870969 33397157

[46] Hu Y Chen X Wang S Jing Y Su J . Subchondral bone microenvironment in osteoarthritis and pain. Bone Res. (2021) 9:20. doi: 10.1038/s41413-021-00147-z 33731688 PMC7969608

[47] Walsh DA Bonnet CS Turner EL Wilson D Situ M McWilliams DF . Angiogenesis in the synovium and at the osteochondral junction in osteoarthritis. Osteoarthritis Cartilage. (2007) 15:743–51. doi: 10.1016/j.joca.2007.01.020 17376709

[48] Aso K Shahtaheri SM Hill R Wilson D McWilliams DF Nwosu LN . Contribution of nerves within osteochondral channels to osteoarthritis knee pain in humans and rats. Osteoarthritis Cartilage. (2020) 28:1245–54. doi: 10.1016/j.joca.2020.05.010 32470596

[49] Walsh DA McWilliams DF Turley MJ Dixon MR Fransès RE Mapp PI . Angiogenesis and nerve growth factor at the osteochondral junction in rheumatoid arthritis and osteoarthritis. Rheumatol (Oxford England). (2010) 49:1852–61. doi: 10.1093/rheumatology/keq188 20581375 PMC2936950

[50] Clockaerts S Bastiaansen-Jenniskens YM Runhaar J Van Osch GJ Van Offel JF Verhaar JA . The infrapatellar fat pad should be considered as an active osteoarthritic joint tissue: a narrative review. Osteoarthritis Cartilage. (2010) 18:876–82. doi: 10.1016/j.joca.2010.03.014 20417297

[51] Macchi V Stocco E Stecco C Belluzzi E Favero M Porzionato A . The infrapatellar fat pad and the synovial membrane: An anatomo-functional unit. J Anat. (2018) 233:146–54. doi: 10.1111/joa.12820 29761471 PMC6036933

[52] Takano S Uchida K Inoue G Miyagi M Aikawa J Iwase D . Nerve growth factor regulation and production by macrophages in osteoarthritic synovium. Clin Exp Immunol. (2017) 190:235–43. doi: 10.1111/cei.13007 28677145 PMC5629442

[53] Bondeson J Wainwright SD Lauder S Amos N Hughes CE . The role of synovial macrophages and macrophage-produced cytokines in driving aggrecanases, matrix metalloproteinases, and other destructive and inflammatory responses in osteoarthritis. Arthritis Res Ther. (2006) 8:R187. doi: 10.1186/ar2099 17177994 PMC1794533

[54] Cameron-Donaldson M Holland C Hungerford DS Frondoza CG . Cartilage debris increases the expression of chondrodestructive tumor necrosis factor-alpha by articular chondrocytes. Arthroscopy: J Arthroscopic Related Surgery: Off Publ Arthroscopy Assoc North America Int Arthroscopy Assoc. (2004) 20:1040–3. doi: 10.1016/j.arthro.2004.08.009 15592232

[55] Guerne PA Carson DA Lotz M . Il-6 production by human articular chondrocytes. Modulation of its synthesis by cytokines, growth factors, and hormones *in vitro*. J Immunol (Baltimore Md 1950). (1990) 144:499–505. doi: 10.4049/jimmunol.144.2.499 2104896

[56] Pelletier JP McCollum R Cloutier JM Martel-Pelletier J . Synthesis of metalloproteases and interleukin 6 (Il-6) in human osteoarthritic synovial membrane is an Il-1 mediated process. J Rheumatol Supplement. (1995) 43:109–14. 7752112

[57] Clockaerts S Bastiaansen-Jenniskens YM Feijt C De Clerck L Verhaar JA Zuurmond AM . Cytokine production by infrapatellar fat pad can be stimulated by interleukin 1β and inhibited by peroxisome proliferator activated receptor α agonist. Ann Rheumatic Dis. (2012) 71:1012–8. doi: 10.1136/annrheumdis-2011-200688 22307941

[58] Favero M El-Hadi H Belluzzi E Granzotto M Porzionato A Sarasin G . Infrapatellar fat pad features in osteoarthritis: A histopathological and molecular study. Rheumatol (Oxford England). (2017) 56:1784–93. doi: 10.1093/rheumatology/kex287 28957567

[59] Snelling SJ Bas S Puskas GJ Dakin SG Suva D Finckh A . Presence of Il-17 in synovial fluid identifies a potential inflammatory osteoarthritic phenotype. PloS One. (2017) 12:e0175109. doi: 10.1371/journal.pone.0175109 28399156 PMC5388337

[60] Chen B Deng Y Tan Y Qin J Chen LB . Association between severity of knee osteoarthritis and serum and synovial fluid interleukin 17 concentrations. J Int Med Res. (2014) 42:138–44. doi: 10.1177/0300060513501751 24319050

[61] Kamel S Khalaf R Moness H Ahmed S . Serum and synovial fluid levels of interleukin-17a in primary knee osteoarthritis patients: Correlations with functional status, pain, and disease severity. Arch Rheumatol. (2022) 37:187–94. doi: 10.46497/ArchRheumatol.2022.7931 36017202 PMC9377169

[62] Nees TA Rosshirt N Zhang JA Platzer H Sorbi R Tripel E . T helper cell infiltration in osteoarthritis-related knee pain and disability. J Clin Med. (2020) 9. doi: 10.3390/jcm9082423 32751139 PMC7464429

[63] Rosshirt N Trauth R Platzer H Tripel E Nees TA Lorenz HM . Proinflammatory T cell polarization is already present in patients with early knee osteoarthritis. Arthritis Res Ther. (2021) 23:37. doi: 10.1186/s13075-020-02410-w 33482899 PMC7821658

[64] Sun L Wang X Kaplan DL . A 3d cartilage - inflammatory cell culture system for the modeling of human osteoarthritis. Biomaterials. (2011) 32:5581–9. doi: 10.1016/j.biomaterials.2011.04.028 21565399 PMC3109142

[65] Ferrao Blanco MN Bastiaansen-Jenniskens YM Chambers MG Pitsillides AA Narcisi R van Osch G . Effect of inflammatory signaling on human articular chondrocyte hypertrophy: Potential involvement of tissue repair macrophages. Cartilage. (2021) 13:168s–74s. doi: 10.1177/19476035211021907 34165367 PMC8739598

[66] Donato AC Belluzzi E Masola V Ruggieri P Brun P . Lactose-modified hyaluronic acid molecule attenuates *in vitro* chondrocyte inflammation. Cells. (2025) 14. doi: 10.3390/cells14241977 41439997 PMC12731145

[67] Na HS Park JS Cho KH Kwon JY Choi J Jhun J . Interleukin-1-interleukin-17 signaling axis induces cartilage destruction and promotes experimental osteoarthritis. Front Immunol. (2020) 11:730. doi: 10.3389/fimmu.2020.00730 32431699 PMC7214841

[68] Scanzello CR . Chemokines and inflammation in osteoarthritis: Insights from patients and animal models. J Orthopaedic Research: Off Publ Orthopaedic Res Soc. (2017) 35:735–9. doi: 10.1002/jor.23471 27808445 PMC5912941

[69] Zhang Y Liu D Vithran DTA Kwabena BR Xiao W Li Y . Cc chemokines and receptors in osteoarthritis: New insights and potential targets. Arthritis Res Ther. (2023) 25:113. doi: 10.1186/s13075-023-03096-6 37400871 PMC10316577

[70] Luo H Li L Han S Liu T . The role of monocyte/macrophage chemokines in pathogenesis of osteoarthritis: A review. Int J Immunogenet. (2024) 51:130–42. doi: 10.1111/iji.12664 38462560

[71] Borzì RM Mazzetti I Marcu KB Facchini A . Chemokines in cartilage degradation. Clin Orthopaedics Related Res. (2004), S53–61. doi: 10.1097/01.blo.0000143805.64755.4f 15480075

[72] Miller RE Miller RJ Malfait AM . Osteoarthritis joint pain: The cytokine connection. Cytokine. (2014) 70:185–93. doi: 10.1016/j.cyto.2014.06.019 25066335 PMC4254338

[73] Li J Chen H Zhang D Xie J Zhou X . The role of stromal cell-derived factor 1 on cartilage development and disease. Osteoarthritis Cartilage. (2021) 29:313–22. doi: 10.1016/j.joca.2020.10.010 33253889

[74] Eymard F Pigenet A Citadelle D Flouzat-Lachaniette CH Poignard A Benelli C . Induction of an inflammatory and prodegradative phenotype in autologous fibroblast-like synoviocytes by the infrapatellar fat pad from patients with knee osteoarthritis. Arthritis Rheumatol (Hoboken NJ). (2014) 66:2165–74. doi: 10.1002/art.38657 24719336

[75] Li R Guan Z Bi S Wang F He L Niu X . The proton-activated G protein-coupled receptor Gpr4 regulates the development of osteoarthritis via modulating Cxcl12/Cxcr7 signaling. Cell Death Dis. (2022) 13:152. doi: 10.1038/s41419-021-04455-4 35165253 PMC8844071

[76] Lin C Liu L Zeng C Cui ZK Chen Y Lai P . Activation of Mtorc1 in subchondral bone preosteoblasts promotes osteoarthritis by stimulating bone sclerosis and secretion of Cxcl12. Bone Res. (2019) 7:5. doi: 10.1038/s41413-018-0041-8 30792936 PMC6381187

[77] Lu J Wu Z Xiong Y . Knockdown of long noncoding RNA Hotair inhibits osteoarthritis chondrocyte injury by Mir-107/Cxcl12 axis. J Orthop Surg Res. (2021) 16:410. doi: 10.1186/s13018-021-02547-7 34183035 PMC8237457

[78] Sheng C Zhou X Wang J Yang Y Xie Z Wu M . Jiawei Yanghe decoction alleviates osteoporotic osteoarthritis by promoting Msc osteogenic differentiation and homing via Itgb6/Tgf-β/Cxcr4 pathway. Phytomedicine. (2025) 147:157203. doi: 10.1016/j.phymed.2025.157203 40929885

[79] Wei J Yang X Zhao L Liu X Ge X Zhang J . Fire needling acupuncture attenuates synovial inflammation and cartilage degeneration in knee osteoarthritis via Sdf-1/Cxcr4-mediated macrophage polarization. Clin Rheumatol. (2025) 44:4283–99. doi: 10.1007/s10067-025-07612-8 40779147

[80] Ruths L de Hesselle H Haußner F Albers S Harms M Freitag T . Targeting the Cxcl12/Cxcr4 pathway by an optimized derivative or Epi-X4 preserves chondrocyte function and offers a novel therapeutic approach in rheumatic diseases. Arthritis Res Ther. (2025) 27:222. doi: 10.1186/s13075-025-03691-9 41331944 PMC12670779

[81] Appleton CT Usmani SE Pest MA Pitelka V Mort JS Beier F . Reduction in disease progression by inhibition of transforming growth factor α-Ccl2 signaling in experimental posttraumatic osteoarthritis. Arthritis Rheumatol. (2015) 67:2691–701. doi: 10.1002/art.39255 26138996

[82] Eisinger K Bauer S Schäffler A Walter R Neumann E Buechler C . Chemerin induces Ccl2 and Tlr4 in synovial fibroblasts of patients with rheumatoid arthritis and osteoarthritis. Exp Mol Pathol. (2012) 92:90–6. doi: 10.1016/j.yexmp.2011.10.006 22037282

[83] Tao Z Zhou Y Zeng B Yang X Su M . Microrna-183 attenuates osteoarthritic pain by inhibiting the Tgfα-mediated Ccl2/Ccr2 signalling axis. Bone Joint Res. (2021) 10:548–57. doi: 10.1302/2046-3758.108.Bjr-2019-0308.R2 34463129 PMC8414439

[84] Pulsatelli L Dolzani P Piacentini A Silvestri T Ruggeri R Gualtieri G . Chemokine production by human chondrocytes. J Rheumatol. (1999) 26:1992–2001. 10493682

[85] Sandell LJ Xing X Franz C Davies S Chang LW Patra D . Exuberant expression of chemokine genes by adult human articular chondrocytes in response to Il-1beta. Osteoarthritis Cartilage. (2008) 16:1560–71. doi: 10.1016/j.joca.2008.04.027 18565769 PMC2605974

[86] Willcockson H Ozkan H Chubinskaya S Loeser RF Longobardi L . Ccl2 induces articular chondrocyte Mmp expression through Erk and P38 signaling pathways. Osteoarthritis Cartilage Open. (2021) 3:100136. doi: 10.1016/j.ocarto.2020.100136 36475068 PMC9718225

[87] Wang Z Wang B Zhang J Wu Z Yu L Sun Z . Chemokine (C-C motif) ligand 2/chemokine receptor 2 (Ccr2) axis blockade to delay chondrocyte hypertrophy as a therapeutic strategy for osteoarthritis. Med Sci Monitor: Int Med J Exp Clin Res. (2021) 27:e930053. doi: 10.12659/msm.930053 34876548 PMC8667482

[88] Miller RE Tran PB Das R Ghoreishi-Haack N Ren D Miller RJ . Ccr2 chemokine receptor signaling mediates pain in experimental osteoarthritis. PNAS. (2012) 109:20602–7. doi: 10.1073/pnas.1209294110 23185004 PMC3528555

[89] Miotla Zarebska J Chanalaris A Driscoll C Burleigh A Miller RE Malfait AM . Ccl2 and Ccr2 regulate pain-related behaviour and early gene expression in post-traumatic murine osteoarthritis but contribute little to chondropathy. Osteoarthritis Cartilage. (2017) 25:406–12. doi: 10.1016/j.joca.2016.10.008 27746376 PMC5358501

[90] Longobardi L Jordan JM Shi XA Renner JB Schwartz TA Nelson AE . Associations between the chemokine biomarker Ccl2 and knee osteoarthritis outcomes: The Johnston County Osteoarthritis Project. Osteoarthritis Cartilage. (2018) 26:1257–61. doi: 10.1016/j.joca.2018.04.012 29723633 PMC6098742

[91] Collins KH Lenz KL Pollitt EN Ferguson D Hutson I Springer LE . Adipose tissue is a critical regulator of osteoarthritis. PNAS. (2021) 118. doi: 10.1073/pnas.2021096118 33443201 PMC7817130

[92] Ito A Suganami T Yamauchi A Degawa-Yamauchi M Tanaka M Kouyama R . Role of Cc chemokine receptor 2 in bone marrow cells in the recruitment of macrophages into obese adipose tissue. J Biol Chem. (2008) 283:35715–23. doi: 10.1074/jbc.M804220200 18977759

[93] Pierzchala AW Kusz DJ Hajduk G . Cxcl8 and Ccl5 expression in synovial fluid and blood serum in patients with osteoarthritis of the knee. Archivum Immunologiae Therapiae Experimentalis. (2011) 59:151–5. doi: 10.1007/s00005-011-0115-4 21336628

[94] Yang P Tan J Yuan Z Meng G Bi L Liu J . Expression profile of cytokines and chemokines in osteoarthritis patients: Proinflammatory roles for Cxcl8 and Cxcl11 to chondrocytes. Int Immunopharmacol. (2016) 40:16–23. doi: 10.1016/j.intimp.2016.08.005 27567247

[95] Rajarathnam K Schnoor M Richardson RM Rajagopal S . How do chemokines navigate neutrophils to the target site: Dissecting the structural mechanisms and signaling pathways. Cell Signalling. (2019) 54:69–80. doi: 10.1016/j.cellsig.2018.11.004 30465827 PMC6664297

[96] Steinbeck MJ Nesti LJ Sharkey PF Parvizi J . Myeloperoxidase and chlorinated peptides in osteoarthritis: Potential biomarkers of the disease. J Orthopaedic Research: Off Publ Orthopaedic Res Soc. (2007) 25:1128–35. doi: 10.1002/jor.20400 17474133 PMC2954494

[97] Wang G Jing W Bi Y Li Y Ma L Yang H . Neutrophil elastase induces chondrocyte apoptosis and facilitates the occurrence of osteoarthritis via caspase signaling pathway. Front Pharmacol. (2021) 12:666162. doi: 10.3389/fphar.2021.666162 33935789 PMC8080035

[98] van Lent PL Blom AB Schelbergen RF Slöetjes A Lafeber FP Lems WF . Active involvement of alarmins S100a8 and S100a9 in the regulation of synovial activation and joint destruction during mouse and human osteoarthritis. Arthritis Rheumatism. (2012) 64:1466–76. doi: 10.1002/art.34315 22143922

[99] Schelbergen RF Blom AB van den Bosch MH Slöetjes A Abdollahi-Roodsaz S Schreurs BW . Alarmins S100a8 and S100a9 elicit a catabolic effect in human osteoarthritic chondrocytes that is dependent on toll-like receptor 4. Arthritis Rheumatism. (2012) 64:1477–87. doi: 10.1002/art.33495 22127564

[100] Luan T Yang X Kuang G Wang T He J Liu Z . Identification and analysis of neutrophil extracellular trap-related genes in osteoarthritis by bioinformatics and experimental verification. J Inflammation Res. (2023) 16:3837–52. doi: 10.2147/jir.S414452 37671131 PMC10476866

[101] Leung YY Huebner JL Haaland B Wong SBS Kraus VB . Synovial fluid pro-inflammatory profile differs according to the characteristics of knee pain. Osteoarthritis Cartilage. (2017) 25:1420–7. doi: 10.1016/j.joca.2017.04.001 28433814

[102] García-Manrique M Calvet J Orellana C Berenguer-Llergo A Garcia-Cirera S Llop M . Synovial fluid but not plasma interleukin-8 is associated with clinical severity and inflammatory markers in knee osteoarthritis women with joint effusion. Sci Rep. (2021) 11:5258. doi: 10.1038/s41598-021-84582-2 33664374 PMC7933151

[103] Hou SM Hou CH Liu JF . Cx3cl1 promotes Mmp-3 production via the Cx3cr1, C-Raf, Mek, Erk, and Nf-Kb signaling pathway in osteoarthritis synovial fibroblasts. Arthritis Res Ther. (2017) 19:282. doi: 10.1186/s13075-017-1487-6 29268768 PMC5740560

[104] Wojdasiewicz P Poniatowski LA Kotela A Deszczyński J Kotela I Szukiewicz D . The chemokine Cx3cl1 (fractalkine) and its receptor Cx3cr1: occurrence and potential role in osteoarthritis. Arch Immunol Ther Exp (Warsz). (2014) 62:395–403. doi: 10.1007/s00005-014-0275-0 24556958 PMC4164853

[105] Liu X Li G Yang L Pan N Tang S Yu C . Circular rna Pvt1 promotes osteoarthritis progression through the Mir-550a-3p/Cx3cr1 pathway: a mechanistic study. Int Immunopharmacol. (2025) 163:115272. doi: 10.1016/j.intimp.2025.115272 40730119

[106] Liu XC Xu L Cai YL Zheng ZY Dai EN Sun S . Mir-1207-5p/Cx3cr1 axis regulates the progression of osteoarthritis via the modulation of the activity of Nf-Kb pathway. Int J Rheum Dis. (2020) 23:1057–65. doi: 10.1111/1756-185x.13898 32597559

[107] Sun Y Wang F Sun X Wang X Zhang L Li Y . Cx3cr1 regulates osteoarthrosis chondrocyte proliferation and apoptosis via Wnt/B-catenin signaling. BioMed Pharmacother. (2017) 96:1317–23. doi: 10.1016/j.biopha.2017.11.080 29217163

[108] Scanzello CR Plaas A Crow MK . Innate immune system activation in osteoarthritis: is osteoarthritis a chronic wound? Curr Opin Rheumatol. (2008) 20:565–72. doi: 10.1097/BOR.0b013e32830aba34 18698179

[109] Hwang HS Park SJ Cheon EJ Lee MH Kim HA . Fibronectin fragment-induced expression of matrix metalloproteinases is mediated by Myd88-dependent Tlr-2 signaling pathway in human chondrocytes. Arthritis Res Ther. (2015) 17:320. doi: 10.1186/s13075-015-0833-9 26563875 PMC4643537

[110] Campo GM Avenoso A Campo S D'Ascola A Nastasi G Calatroni A . Small hyaluronan oligosaccharides induce inflammation by engaging both Toll-like-4 and Cd44 receptors in human chondrocytes. Biochem Pharmacol. (2010) 80:480–90. doi: 10.1016/j.bcp.2010.04.024 20435021

[111] Loeser RF . Integrins and chondrocyte-matrix interactions in articular cartilage. Matrix Biology: J Int Soc For Matrix Biol. (2014) 39:11–6. doi: 10.1016/j.matbio.2014.08.007 25169886 PMC4699681

[112] Zhang Y Lin Z Chen D He Y . Cy-09 attenuates the progression of osteoarthritis via inhibiting Nlrp3 inflammasome-mediated pyroptosis. Biochem Biophys Res Commun. (2021) 553:119–25. doi: 10.1016/j.bbrc.2021.03.055 33765556

[113] Zhou S Liu G Si Z Yu L Hou L . Glycyrrhizin, an Hmgb1 inhibitor, suppresses interleukin-1β-induced inflammatory responses in chondrocytes from patients with osteoarthritis. Cartilage. (2021) 13:947s–55s. doi: 10.1177/1947603520934858 32602358 PMC8804755

[114] Millerand M Sudre L Nefla M Pène F Rousseau C Pons A . Activation of innate immunity by 14-3-3 E, a new potential alarmin in osteoarthritis. Osteoarthritis Cartilage. (2020) 28:646–57. doi: 10.1016/j.joca.2020.03.002 32173627

[115] Schelbergen RF van Dalen S ter Huurne M Roth J Vogl T Noël D . Treatment efficacy of adipose-derived stem cells in experimental osteoarthritis is driven by high synovial activation and reflected by S100a8/A9 serum levels. Osteoarthritis Cartilage. (2014) 22:1158–66. doi: 10.1016/j.joca.2014.05.022 24928317

[116] van Kooten NJT Blom AB Teunissen van Manen IJ Theeuwes WF Roth J Gorris MAJ . S100a8/A9 drives monocytes towards M2-like macrophage differentiation and associates with M2-like macrophages in osteoarthritic synovium. Rheumatol (Oxford). (2025) 64:332–43. doi: 10.1093/rheumatology/keae020 38216750 PMC11701306

[117] Chen Y Dong S Zeng X Xu Q Liang M Liao G . Ezh2/Mir-142-3p/Hmgb1 axis mediates chondrocyte pyroptosis by regulating endoplasmic reticulum stress in knee osteoarthritis. Chin Med J (Engl). (2025) 138:79–92. doi: 10.1097/cm9.0000000000003186 39704001 PMC11717515

[118] Li S Li Y Hou L Tang L Gao F . Forsythoside B alleviates osteoarthritis through the Hmgb1/Tlr4/Nf-Kb and Keap1/Nrf2/Ho-1 pathways. J Biochem Mol Toxicol. (2024) 38:e23569. doi: 10.1002/jbt.23569 37943572

[119] Sun H Chu X Qian W Yin H . Circmyo1c silencing alleviates chondrocytes inflammation and apoptosis through M(6)a/Hmgb1 axis in osteoarthritis. Biotechnol Appl Biochem. (2024) 71:1360–9. doi: 10.1002/bab.2635 38982736

[120] Li M Wei CB Li HF He K Bai RJ Zhang FJ . Osteopontin inhibits autophagy via Cd44 and Avβ3 integrin and promotes cell proliferation in osteoarthritic fibroblast-like synoviocytes. BMC Musculoskelet Disord. (2025) 26:274. doi: 10.1186/s12891-025-08509-y 40102843 PMC11916941

[121] Wang T Hao Z Liu C Yuan L Li L Yin M . Mir-193b modulates osteoarthritis progression through targeting St3gal4 via sialylation of Cd44 and Nf-Kb pathway. Cell Signal. (2020) 76:109814. doi: 10.1016/j.cellsig.2020.109814 33080315

[122] Zhang FJ Gao SG Cheng L Tian J Xu WS Luo W . The effect of hyaluronic acid on osteopontin and Cd44 mrna of fibroblast-like synoviocytes in patients with osteoarthritis of the knee. Rheumatol Int. (2013) 33:79–83. doi: 10.1007/s00296-011-2339-3 22218639

[123] Lin Z Li P Tang Y Tan H Luo L . Hsa_circ_0007292 promotes chondrocyte injury in osteoarthritis via targeting the Mir-1179/Hmgb1 axis. J Orthop Surg Res. (2023) 18:544. doi: 10.1186/s13018-023-04026-7 37516834 PMC10386318

[124] Meng Y Qiu S Sun L Zuo J . Knockdown of exosome-mediated Lnc-Pvt1 alleviates lipopolysaccharide-induced osteoarthritis progression by mediating the Hmgb1/Tlr4/Nf-Kb pathway via Mir-93-5p. Mol Med Rep. (2020) 22:5313–25. doi: 10.3892/mmr.2020.11594 33174011 PMC7646997

[125] Qiu M Liu D Fu Q . Mir-129-5p shuttled by human synovial mesenchymal stem cell-derived exosomes relieves Il-1β induced osteoarthritis via targeting Hmgb1. Life Sci. (2021) 269:118987. doi: 10.1016/j.lfs.2020.118987 33417958

[126] Wang X Guo Y Wang C Yu H Yu X Yu H . Microrna-142-3p inhibits chondrocyte apoptosis and inflammation in osteoarthritis by targeting Hmgb1. Inflammation. (2016) 39:1718–28. doi: 10.1007/s10753-016-0406-3 27447821

[127] Xiong T Huang S Wang X Shi Y He J Yuan Y . N-3 polyunsaturated fatty acids alleviate the progression of obesity-related osteoarthritis and protect cartilage through inhibiting the Hmgb1-Rage/Tlr4 signaling pathway. Int Immunopharmacol. (2024) 128:111498. doi: 10.1016/j.intimp.2024.111498 38218011

[128] Abo-Zalam HB Gendy AM Ali BM Ragab GM Selim H Hamed NO . Acetyl-11-keto-beta-boswellic acid ameliorates monosodium iodoacetate-induced osteoarthritis in rats: implications of Hmgb1/Tlr4/Nf-Kb and Nrf2/Ho-1. Front Pharmacol. (2025) 16:1694803. doi: 10.3389/fphar.2025.1694803 41403440 PMC12702862

[129] Jiang Y Zhu L Zhang T Lu H Wang C Xue B . Brd4 has dual effects on the Hmgb1 and Nf-Kb signalling pathways and is a potential therapeutic target for osteoarthritis. Biochim Biophys Acta Mol Basis Dis. (2017) 1863:3001–15. doi: 10.1016/j.bbadis.2017.08.009 28844955

[130] Li W Tao C Mao M Zhu K . The Nrf2/Hmgb1/Nf-Kb axis modulates chondrocyte apoptosis and extracellular matrix degradation in osteoarthritis. Acta Biochim Biophys Sin. (2023) 55:818–30. doi: 10.3724/abbs.2023078 37232576 PMC10281874

[131] Chen Z Zhou M Luo J Li C Li S Lin S . Targeted S100a8 pp-Cas9@Plga-Apt microparticles reduced cartilage degradation and subchondral bone isomerism in osteoarthritis treatment through Jak/Stat3 pathway modulation. Mater Today Bio. (2025) 35:102354. doi: 10.1016/j.mtbio.2025.102354 41111484 PMC12528939

[132] Schelbergen RF Geven EJ van den Bosch MH Eriksson H Leanderson T Vogl T . Prophylactic treatment with S100a9 inhibitor Paquinimod reduces pathology in experimental collagenase-induced osteoarthritis. Ann Rheum Dis. (2015) 74:2254–8. doi: 10.1136/annrheumdis-2014-206517 25969431

[133] Deng C Chen Y Zhao X Yu L Xiao Y Li H . Apoptotic neutrophil membrane-camouflaged liposomes for dually targeting synovial macrophages and fibroblasts to attenuate osteoarthritis. ACS Appl Mater Interfaces. (2023) 15:39064–80. doi: 10.1021/acsami.3c05861 37523857

[134] Yu Q Huang Y Chen X Chen Y Zhu X Liu Y . A neutrophil cell membrane-biomimetic nanoplatform based on L-arginine nanoparticles for early osteoarthritis diagnosis and nitric oxide therapy. Nanoscale. (2022) 14:11619–34. doi: 10.1039/d2nr02601e 35894521

[135] Bondeson J Wainwright S Hughes C Caterson B . The regulation of the Adamts4 and Adamts5 aggrecanases in osteoarthritis: a review. Clin Exp Rheumatol. (2008) 26:139–45. 18328163

[136] Jiang L Lin J Zhao S Wu J Jin Y Yu L . Adamts5 in osteoarthritis: biological functions, regulatory network, and potential targeting therapies. Front Mol Biosci. (2021) 8:703110. doi: 10.3389/fmolb.2021.703110 34434966 PMC8381022

[137] Zack MD Arner EC Anglin CP Alston JT Malfait AM Tortorella MD . Identification of fibronectin neoepitopes present in human osteoarthritic cartilage. Arthritis Rheumatism. (2006) 54:2912–22. doi: 10.1002/art.22045 16948117

[138] Xie DL Meyers R Homandberg GA . Fibronectin fragments in osteoarthritic synovial fluid. J Rheumatol. (1992) 19:1448–52. 1433014

[139] Homandberg GA Costa V Wen C . Fibronectin fragments active in chondrocytic chondrolysis can be chemically cross-linked to the alpha5 integrin receptor subunit. Osteoarthritis Cartilage. (2002) 10:938–49. doi: 10.1053/joca.2002.0854 12464554

[140] Forsyth CB Pulai J Loeser RF . Fibronectin fragments and blocking antibodies to alpha2beta1 and alpha5beta1 integrins stimulate mitogen-activated protein kinase signaling and increase collagenase 3 (matrix metalloproteinase 13) production by human articular chondrocytes. Arthritis Rheumatism. (2002) 46:2368–76. doi: 10.1002/art.10502 12355484

[141] Pulai JI Chen H Im HJ Kumar S Hanning C Hegde PS . Nf-kappa B mediates the stimulation of cytokine and chemokine expression by human articular chondrocytes in response to fibronectin fragments. J Immunol (Baltimore Md 1950). (2005) 174:5781–8. doi: 10.4049/jimmunol.174.9.5781 15843581 PMC2903737

[142] Jennings L Wu L King KB Hämmerle H Cs-Szabo G Mollenhauer J . The effects of collagen fragments on the extracellular matrix metabolism of bovine and human chondrocytes. Connective Tissue Res. (2001) 42:71–86. doi: 10.3109/03008200109014250 11696990

[143] Liu-Bryan R Terkeltaub R . Chondrocyte innate immune myeloid differentiation factor 88-dependent signaling drives procatabolic effects of the endogenous Toll-like receptor 2/Toll-like receptor 4 ligands low molecular weight hyaluronan and high mobility group box chromosomal protein 1 in mice. Arthritis Rheumatism. (2010) 62:2004–12. doi: 10.1002/art.27475 20506365 PMC2902571

[144] Brun P Panfilo S Daga Gordini D Cortivo R Abatangelo G . The effect of hyaluronan on Cd44-mediated survival of normal and hydroxyl radical-damaged chondrocytes. Osteoarthritis Cartilage. (2003) 11:208–16. doi: 10.1016/s1063-4584(02)00352-7 12623292

[145] Julovi SM Ito H Nishitani K Jackson CJ Nakamura T . Hyaluronan inhibits matrix metalloproteinase-13 in human arthritic chondrocytes via Cd44 and p38. J Orthopaedic Research: Off Publ Orthopaedic Res Soc. (2011) 29:258–64. doi: 10.1002/jor.21216 20706991

[146] Barreto G Senturk B Colombo L Brück O Neidenbach P Salzmann G . Lumican is upregulated in osteoarthritis and contributes to Tlr4-induced pro-inflammatory activation of cartilage degradation and macrophage polarization. Osteoarthritis Cartilage. (2020) 28:92–101. doi: 10.1016/j.joca.2019.10.011 31715293

[147] Lin J Huang Y Lin X Liu W Wu X Qiu H . Bauhinia championii alleviates extracellular matrix degradation in Il-1β induced chondrocytes via Mirna-145-5p/Tlr4/Nf-Kb axis. Heliyon. (2023) 9:e19138. doi: 10.1016/j.heliyon.2023.e19138 37664703 PMC10469563

[148] Sun W Liao Y Feng J Liang J He Q Cui Y . Targeting of Cirp attenuates osteoarthritis progression via suppressing Tlr4/Nf-Kb/Nlrp3 signaling axis. Int J Mol Med. (2026) 57. doi: 10.3892/ijmm.2025.5674 41133454 PMC12582850

[149] Zhang Y Zhao P Li S Mu X Wang H . Circscaper knockdown attenuates Il-1β-induced chondrocyte injury by Mir-127-5p/Tlr4 axis in osteoarthritis. Autoimmunity. (2022) 55:577–86. doi: 10.1080/08916934.2022.2103798 35993243

[150] Guo D Ding L Homandberg GA . Telopeptides of type II collagen upregulate proteinases and damage cartilage but are less effective than highly active fibronectin fragments. Inflammation Research: Off J Eur Histamine Res Soc [et al]. (2009) 58:161–9. doi: 10.1007/s00011-009-8090-5 19190855

[151] Homandberg GA . Potential regulation of cartilage metabolism in osteoarthritis by fibronectin fragments. Front Bioscience A J Virtual Library. (1999) 4:D713-30. doi: 10.2741/homandberg 10525477

[152] Ding L Guo D Homandberg GA Buckwalter JA Martin JA . A single blunt impact on cartilage promotes fibronectin fragmentation and upregulates cartilage degrading stromelysin-1/matrix metalloproteinase-3 in a bovine ex vivo model. J Orthopaedic Res Off Publ Orthopaedic Res Soc. (2014) 32:811–8. doi: 10.1002/jor.22610 24610678 PMC4034576

[153] Millward-Sadler SJ Salter DM . Integrin-dependent signal cascades in chondrocyte mechanotransduction. Ann BioMed Eng. (2004) 32:435–46. doi: 10.1023/b:abme.0000017538.72511.48 15095818

[154] Ding L Guo D Homandberg GA . Fibronectin fragments mediate matrix metalloproteinase upregulation and cartilage damage through proline rich tyrosine kinase 2, c-src, nf-kappab and protein kinase cdelta. Osteoarthritis Cartilage. (2009) 17:1385–92. doi: 10.1016/j.joca.2009.03.024 19409294

[155] Miao MZ Su QP Cui Y Bahnson EM Li G Wang M . Redox-active endosomes mediate α5β1 integrin signaling and promote chondrocyte matrix metalloproteinase production in osteoarthritis. Sci Signaling. (2023) 16:eadf8299. doi: 10.1126/scisignal.adf8299 37906629 PMC10666734

[156] Cui Y Miao MZ Wang M Su QP Qiu K Arbeeva L . Yes-associated protein nuclear translocation promotes anabolic activity in human articular chondrocytes. Osteoarthritis Cartilage. (2023) 31:1078–90. doi: 10.1016/j.joca.2023.04.006 37100374 PMC10524185

[157] Reed KSM Ulici V Kim C Chubinskaya S Loeser RF Phanstiel DH . Transcriptional response of human articular chondrocytes treated with fibronectin fragments: an *in vitro* model of the osteoarthritis phenotype. Osteoarthritis Cartilage. (2021) 29:235–47. doi: 10.1016/j.joca.2020.09.006 33248223 PMC7870543

[158] Fu W Hettinghouse A Chen Y Hu W Ding X Chen M . 14-3-3 epsilon is an intracellular component of TNFR2 receptor complex and its activation protects against osteoarthritis. Ann Rheumatic Dis. (2021) 80:1615–27. doi: 10.1136/annrheumdis-2021-220000 34226187 PMC8595573

[159] Priam S Bougault C Houard X Gosset M Salvat C Berenbaum F . Identification of soluble 14-3-3∈ as a novel subchondral bone mediator involved in cartilage degradation in osteoarthritis. Arthritis Rheumatism. (2013) 65:1831–42. doi: 10.1002/art.37951 23552998

[160] Nefla M Sudre L Denat G Priam S Andre-Leroux G Berenbaum F . The pro-inflammatory cytokine 14-3-3ϵ is a ligand of CD13 in cartilage. J Cell Sci. (2015) 128:3250–62. doi: 10.1242/jcs.169573 26208633 PMC4582189

[161] Hodgkinson T Kelly DC Curtin CM O'Brien FJ . Mechanosignalling in cartilage: an emerging target for the treatment of osteoarthritis. Nat Rev Rheumatol. (2022) 18:67–84. doi: 10.1038/s41584-021-00724-w 34934171

[162] Huang K Cai H . Matrix stiffness in osteoarthritis: from mechanism introduction to biomaterial-based therapies. Front Endocrinol. (2025) 16:1571502. doi: 10.3389/fendo.2025.1571502 40405967 PMC12095870

[163] Hodgkinson T Amado IN O'Brien FJ Kennedy OD . The role of mechanobiology in bone and cartilage model systems in characterizing initiation and progression of osteoarthritis. APL Bioeng. (2022) 6:011501. doi: 10.1063/5.0068277 40746901 PMC12313331

[164] Sanchez C Lambert C Dubuc JE Bertrand J Pap T Henrotin Y . Syndecan-4 is increased in osteoarthritic knee, but not hip or shoulder, articular hypertrophic chondrocytes. Cartilage. (2021) 13:862s–71s. doi: 10.1177/1947603519870855 31455087 PMC8804772

[165] Guilak F . Biomechanical factors in osteoarthritis. Best Pract Res Clin Rheumatol. (2011) 25:815–23. doi: 10.1016/j.berh.2011.11.013 22265263 PMC3266544

[166] Chen L Zheng JJY Li G Yuan J Ebert JR Li H . Pathogenesis and clinical management of obesity-related knee osteoarthritis: impact of mechanical loading. J Orthopaedic Translation. (2020) 24:66–75. doi: 10.1016/j.jot.2020.05.001 32695606 PMC7349942

[167] Feng R Hu W Li Y Yao X Li J Li X . Mechanotransduction in subchondral bone microenvironment and targeted interventions for osteoarthritis. Mechanobiology Med. (2024) 2:100043. doi: 10.1016/j.mbm.2024.100043 40395860 PMC12082324

[168] Hashemi Motahar HS Eivazitork M Sadeghi MR . Targeting mechanotransduction components as therapeutic strategies in osteoarthritis. Mol Biol Rep. (2026) 53. doi: 10.1007/s11033-026-11622-3 41790317

[169] Zhang X Cai D Zhou F Yu J Wu X Yu D . Targeting downstream subcellular YAP activity as a function of matrix stiffness with verteporfin-encapsulated chitosan microsphere attenuates osteoarthritis. Biomaterials. (2020) 232:119724. doi: 10.1016/j.biomaterials.2019.119724 31918221

[170] Tian J Cheng C Kuang SD Su C Zhao X Xiong YL . OPN deficiency increases the severity of osteoarthritis associated with aberrant chondrocyte senescence and apoptosis and upregulates the expression of osteoarthritis-associated genes. Pain Res Manag. (2020) 2020:3428587. doi: 10.1155/2020/3428587 33144900 PMC7599390

[171] Zeng S Tu M . The lncRNA MIAT/miR-181a-5p axis regulates osteopontin (OPN)-mediated proliferation and apoptosis of human chondrocytes in osteoarthritis. J Mol Histol. (2022) 53:285–96. doi: 10.1007/s10735-022-10067-9 35286539

[172] Liu Q Zeng H Yuan Y Wang Z Wu Z Luo W . Osteopontin inhibits osteoarthritis progression via the OPN/CD44/PI3K signal axis. Genes Dis. (2022) 9:128–39. doi: 10.1016/j.gendis.2020.06.006 35005113 PMC8720673

[173] Bai RJ Liu D Li YS Tian J Yu DJ Li HZ . OPN inhibits autophagy through CD44, integrin and the MAPK pathway in osteoarthritic chondrocytes. Front Endocrinol. (2022) 13:919366. doi: 10.3389/fendo.2022.919366 36034459 PMC9411521

[174] Yuan Y Liu Q Wu Z Luo W . Mechanistic insight on the interaction between OPN and integrin ανβ3 in osteoarthritis. BioMed Res Int. (2020) 2020:2905634. doi: 10.1155/2020/2905634 33134374 PMC7593734

[175] Luo W Lin Z Yuan Y Wu Z Zhong W Liu Q . Osteopontin (OPN) alleviates the progression of osteoarthritis by promoting the anabolism of chondrocytes. Genes Dis. (2023) 10:1714–25. doi: 10.1016/j.gendis.2022.08.010 37397527 PMC10311054

[176] Matsui Y Iwasaki N Kon S Takahashi D Morimoto J Matsui Y . Accelerated development of aging-associated and instability-induced osteoarthritis in osteopontin-deficient mice. Arthritis Rheumatism. (2009) 60:2362–71. doi: 10.1002/art.24705 19644889

[177] Gao SG Zeng C Song Y Tian J Cheng C Yang T . Effect of osteopontin on the mRNA expression of ADAMTS4 and ADAMTS5 in chondrocytes from patients with knee osteoarthritis. Exp Ther Med. (2015) 9:1979–83. doi: 10.3892/etm.2015.2310 26136925 PMC4471803

[178] Li Y Jiang W Wang H Deng Z Zeng C Tu M . Osteopontin promotes expression of matrix metalloproteinase 13 through NF-κB signaling in osteoarthritis. BioMed Res Int. (2016) 2016:6345656. doi: 10.1155/2016/6345656 27656654 PMC5021466

[179] Lin C Chen Z Guo D Zhou L Lin S Li C . Increased expression of osteopontin in subchondral bone promotes bone turnover and remodeling, and accelerates the progression of OA in a mouse model. Aging. (2022) 14:253–71. doi: 10.18632/aging.203707 34982732 PMC8791213

[180] Li L Lv G Wang B Kuang L . XIST/miR-376c-5p/OPN axis modulates the influence of proinflammatory M1 macrophages on osteoarthritis chondrocyte apoptosis. J Cell Physiol. (2020) 235:281–93. doi: 10.1002/jcp.28968 31215024

[181] Jiang B Ma H Pan S Wang J Zhu Y Zhou S . TREM2 modulates CXCL9:SPP1 polarity to influence macrophage-chondrocyte crosstalk and promote osteoarthritis progression. Clin Exp Rheumatol. (2026). doi: 10.55563/clinexprheumatol/3bxp70 42207580

[182] Chow G Nietfeld JJ Knudson CB Knudson W . Antisense inhibition of chondrocyte CD44 expression leading to cartilage chondrolysis. Arthritis Rheumatism. (1998) 41:1411–9. doi: 10.1002/1529-0131(199808)41:8<1411::AID-ART10>3.0.CO;2-Z 9704639

[183] Knudson W Casey B Nishida Y Eger W Kuettner KE Knudson CB . Hyaluronan oligosaccharides perturb cartilage matrix homeostasis and induce chondrocytic chondrolysis. Arthritis Rheumatism. (2000) 43:1165–74. doi: 10.1002/1529-0131(200005)43:5<1165::AID-ANR27>3.0.CO;2-H 10817571

[184] Shimizu H Shimoda M Mochizuki S Miyamae Y Abe H Chijiiwa M . Hyaluronan-binding protein involved in hyaluronan depolymerization is up-regulated and involved in hyaluronan degradation in human osteoarthritic cartilage. Am J Pathol. (2018) 188:2109–19. doi: 10.1016/j.ajpath.2018.05.012 29935163

[185] Iacob S Knudson CB . Hyaluronan fragments activate nitric oxide synthase and the production of nitric oxide by articular chondrocytes. Int J Biochem Cell Biol. (2006) 38:123–33. doi: 10.1016/j.biocel.2005.08.011 16181799 PMC3139231

[186] Almonte-Becerril M Gimeno LI Villarroya O Benito-Jardón M Kouri JB Costell M . Genetic abrogation of the fibronectin-α5β1 integrin interaction in articular cartilage aggravates osteoarthritis in mice. PloS One. (2018) 13:e0198559. doi: 10.1371/journal.pone.0198559 29870552 PMC5988303

[187] Fan A Pang Z Liu Z Yin F Wang Y . Decellularized extracellular matrix restores fibronectin/integrin β1 balance through extracellular vesicles to rejuvenate chondrocytes and alleviate osteoarthritis progression. J Orthop Translat. (2026) 56:101019. doi: 10.1016/j.jot.2025.10.011 41836569 PMC12988504

[188] Zhang C Zhu M Wang H Wen J Huang Z Chen S . LOXL2 attenuates osteoarthritis through inactivating integrin/FAK signaling. Sci Rep. (2021) 11:17020. doi: 10.1038/s41598-021-96348-x 34426599 PMC8382747

[189] Lee W Leddy HA Chen Y Lee SH Zelenski NA McNulty AL . Synergy between Piezo1 and Piezo2 channels confers high-strain mechanosensitivity to articular cartilage. PNAS. (2014) 111:E5114-22. doi: 10.1073/pnas.1414298111 25385580 PMC4250098

[190] Lee W Nims RJ Savadipour A Zhang Q Leddy HA Liu F . Inflammatory signaling sensitizes Piezo1 mechanotransduction in articular chondrocytes as a pathogenic feed-forward mechanism in osteoarthritis. PNAS. (2021) 118. doi: 10.1073/pnas.2001611118 33758095 PMC8020656

[191] He Q Lin Y Chen B Chen C Zeng J Dou X . Vitamin K2 ameliorates osteoarthritis by suppressing ferroptosis and extracellular matrix degradation through activation GPX4's dual functions. BioMed Pharmacother. (2024) 175:116697. doi: 10.1016/j.biopha.2024.116697 38759289

[192] Li Q Gu H Song K Kong X Li Y Liu Z . Lentinan rewrites extracellular matrix homeostasis by activating mitophagy via mTOR/PINK1/Parkin pathway in cartilage to alleviating osteoarthritis. Int J Biol Macromol. (2025) 322:146900. doi: 10.1016/j.ijbiomac.2025.146900 40819752

[193] Ma Y Sun C Wang Z Chen C . Cartilage extracellular matrix regeneration with 10-gingerol via Keap1-Nrf2-ARE axis for osteoarthritis therapy. Arch Pharm Res. (2025) 48:1460–81. doi: 10.1007/s12272-025-01579-5 41212495

[194] Qiu Y Yu B Jiang C Yin H Meng J Wang H . Bone marrow mesenchymal stem cells overexpressing FGF-2 loaded onto a decellularized extracellular matrix hydrogel for the treatment of osteoarthritis. Biomater Sci. (2026) 14:9–30. doi: 10.1039/d5bm00920k 41200806

[195] Wang J Yang J Fang Y Lou C Yu H Li Y . Vinpocetine protects against osteoarthritis by inhibiting ferroptosis and extracellular matrix degradation via activation of the Nrf2/GPX4 pathway. Phytomedicine. (2024) 135:156115. doi: 10.1016/j.phymed.2024.156115 39368343

[196] Kobayashi H Hirata M Saito T Itoh S Chung UI Kawaguchi H . Transcriptional induction of ADAMTS5 protein by nuclear factor-κB (NF-κB) family member RelA/p65 in chondrocytes during osteoarthritis development. J Biol Chem. (2013) 288:28620–9. doi: 10.1074/jbc.M113.452169 23963448 PMC3789961

[197] Saito T Fukai A Mabuchi A Ikeda T Yano F Ohba S . Transcriptional regulation of endochondral ossification by HIF-2alpha during skeletal growth and osteoarthritis development. Nat Med. (2010) 16:678–86. doi: 10.1038/nm.2146 20495570

[198] Zhao X Lin J Liu F Zhang Y Shi B Ma C . Targeting p21-positive senescent chondrocytes via IL-6R/JAK2 inhibition to alleviate osteoarthritis. Advanced Sci (Weinheim Baden-Wurttemberg Germany). (2025) 12:e2410795. doi: 10.1002/advs.202410795 39853717 PMC11923994

[199] Wang X Liu Z Peng P Gong Z Huang J Peng H . Astaxanthin attenuates osteoarthritis progression via inhibiting ferroptosis and regulating mitochondrial function in chondrocytes. Chem Biol Interact. (2022) 366:110148. doi: 10.1016/j.cbi.2022.110148 36084724

[200] Caramés B Taniguchi N Otsuki S Blanco FJ Lotz M . Autophagy is a protective mechanism in normal cartilage, and its aging-related loss is linked with cell death and osteoarthritis. Arthritis Rheumatism. (2010) 62:791–801. doi: 10.1002/art.27305 20187128 PMC2838960

[201] So MW Lee EJ Lee HS Koo BS Kim YG Lee CK . Protective effects of ginsenoside Rg3 on human osteoarthritic chondrocytes. Modern Rheumatol. (2013) 23:104–11. doi: 10.1007/s10165-012-0635-8 22454193

[202] Malfait AM Liu RQ Ijiri K Komiya S Tortorella MD . Inhibition of Adam-Ts4 and Adam-Ts5 prevents aggrecan degradation in osteoarthritic cartilage. J Biol Chem. (2002) 277:22201–8. doi: 10.1074/jbc.M200431200 11956193

[203] Chen CG Thuillier D Chin EN Alliston T . Chondrocyte-intrinsic Smad3 represses Runx2-inducible matrix metalloproteinase 13 expression to maintain articular cartilage and prevent osteoarthritis. Arthritis Rheumatism. (2012) 64:3278–89. doi: 10.1002/art.34566 22674505 PMC3544176

[204] Chen J Chen F Wu X Bian H Chen C Zhang X . Dlx5 promotes Col10a1 expression and chondrocyte hypertrophy and is involved in osteoarthritis progression. Genes Dis. (2023) 10:2097–108. doi: 10.1016/j.gendis.2022.12.016 37492739 PMC10363643

[205] Faust HJ Zhang H Han J Wolf MT Jeon OH Sadtler K . Il-17 and immunologically induced senescence regulate response to injury in osteoarthritis. J Clin Invest. (2020) 130:5493–507. doi: 10.1172/jci134091 32955487 PMC7524483

[206] Jeon OH Kim C Laberge RM Demaria M Rathod S Vasserot AP . Local clearance of senescent cells attenuates the development of post-traumatic osteoarthritis and creates a pro-regenerative environment. Nat Med. (2017) 23:775–81. doi: 10.1038/nm.4324 28436958 PMC5785239

[207] Kim HA Lee YJ Seong SC Choe KW Song YW . Apoptotic chondrocyte death in human osteoarthritis. J Rheumatol. (2000) 27:455–62. 10685814

[208] Zhang L Qiu J Shi J Liu S Zou H . Microrna-140-5p represses chondrocyte pyroptosis and relieves cartilage injury in osteoarthritis by inhibiting cathepsin B/Nod-like receptor protein 3. Bioengineered. (2021) 12:9949–64. doi: 10.1080/21655979.2021.1985342 34565303 PMC8810115

[209] Riegger J Brenner RE . Evidence of necroptosis in osteoarthritic disease: Investigation of blunt mechanical impact as possible trigger in regulated necrosis. Cell Death Dis. (2019) 10:683. doi: 10.1038/s41419-019-1930-5 31527653 PMC6746800

[210] Cheng J Duan X Fu X Jiang Y Yang P Cao C . Rip1 perturbation induces chondrocyte necroptosis and promotes osteoarthritis pathogenesis via targeting Bmp7. Front Cell Dev Biol. (2021) 9:638382. doi: 10.3389/fcell.2021.638382 33937236 PMC8085605

[211] Caramés B Olmer M Kiosses WB Lotz MK . The relationship of autophagy defects to cartilage damage during joint aging in a mouse model. Arthritis Rheumatol (Hoboken NJ). (2015) 67:1568–76. doi: 10.1002/art.39073 25708836 PMC4446178

[212] Platzer H Wellbrock M Pourbozorg G Mayakrishnan R Gantz S Khamees B . In knee osteoarthritis, the production of cytokines and metalloproteinases in presence of chondrocytes and Cd4(+) T cells depends on T cell subset: An *in vitro* analysis. Osteoarthritis Cartilage Open. (2025) 7:100642. doi: 10.1016/j.ocarto.2025.100642 40612887 PMC12226085

[213] Utomo L Bastiaansen-Jenniskens YM Verhaar JA van Osch GJ . Cartilage inflammation and degeneration is enhanced by pro-inflammatory (M1) macrophages *in vitro*, but not inhibited directly by anti-inflammatory (M2) macrophages. Osteoarthritis Cartilage. (2016) 24:2162–70. doi: 10.1016/j.joca.2016.07.018 27502245

[214] Samavedi S Diaz-Rodriguez P Erndt-Marino JD Hahn MS . A three-dimensional chondrocyte-macrophage coculture system to probe inflammation in experimental osteoarthritis. Tissue Eng Part A. (2017) 23:101–14. doi: 10.1089/ten.TEA.2016.0007 27736317 PMC5312455

[215] Wang M Tan G Jiang H Liu A Wu R Li J . Molecular crosstalk between articular cartilage, meniscus, synovium, and subchondral bone in osteoarthritis. Bone Joint Res. (2022) 11:862–72. doi: 10.1302/2046-3758.1112.Bjr-2022-0215.R1 36464496 PMC9792876

[216] Liu Z Sun Y Pan J Guo K Tang Z Wang X . Single-cell profiling uncovers synovial fibroblast subpopulations associated with chondrocyte injury in osteoarthritis. Front Endocrinol. (2024) 15:1479909. doi: 10.3389/fendo.2024.1479909 39720254 PMC11666364

[217] Cesaro G Nagai JS Gnoato N Chiodi A Tussardi G Klöker V . Advances and challenges in cell-cell communication inference: A comprehensive review of tools, resources, and future directions. Briefings Bioinf. (2025) 26. doi: 10.1093/bib/bbaf280 40536815 PMC12204611

[218] Scotece M Pérez T Conde J Abella V López V Pino J . Adipokines induce pro-inflammatory factors in activated Cd4+ T cells from osteoarthritis patient. J Orthopaedic Res Off Publ Orthopaedic Res Soc. (2017) 35:1299–303. doi: 10.1002/jor.23377 27472907

[219] Raut RD Chakraborty AK Neogi T Albro M Snyder B Schaer TP . A multi-tissue human knee single-cell atlas identifies that osteoarthritis reduces regenerative tissue stem cells while increasing inflammatory pain macrophages. Commun Biol. (2025) 8:1146. doi: 10.1038/s42003-025-08586-8 40753276 PMC12317986

[220] Fan Y Bian X Meng X Li L Fu L Zhang Y . Unveiling inflammatory and prehypertrophic cell populations as key contributors to knee cartilage degeneration in osteoarthritis using multi-omics data integration. Ann Rheumatic Dis. (2024) 83:926–44. doi: 10.1136/ard-2023-224420 38325908 PMC11187367

[221] Kulzhanova G Klee A Botros M Hansen VL Reuter J Tashbib ET . Integrative single-cell RNA sequencing and spatial transcriptomics uncover distinct fibroblast-macrophage crosstalk in human hip synovium between patients with femoroacetabular impingement and osteoarthritis. Arthritis Rheumatol (Hoboken NJ). (2026) 78:1219–30. doi: 10.1002/art.70033 41524472

[222] Wu Y Su H Cui Y . Decoding cell-cell communication in spatial transcriptomics: Mechanistic insights, modeling constraints, and analytical caveats. Briefings Bioinf. (2026) 27. doi: 10.1093/bib/bbag477 42704267 PMC13548332

[223] Del Sordo L Blackler GB Philpott HT Riviere J Gunaratnam L Heit B . Impaired efferocytosis by synovial macrophages in patients with knee osteoarthritis. Arthritis Rheumatol (Hoboken NJ). (2023) 75:685–96. doi: 10.1002/art.42412 36448607

[224] Yao Z Qi W Zhang H Zhang Z Liu L Shao Y . Down-regulated Gas6 impairs synovial macrophage efferocytosis and promotes obesity-associated osteoarthritis. eLife. (2023) 12. doi: 10.7554/eLife.83069 37144868 PMC10191622

[225] Ji Q Zheng Y Zhang G Hu Y Fan X Hou Y . Single-cell RNA-seq analysis reveals the progression of human osteoarthritis. Ann Rheumatic Dis. (2019) 78:100–10. doi: 10.1136/annrheumdis-2017-212863 30026257 PMC6317448

[226] Grandi FC Baskar R Smeriglio P Murkherjee S Indelli PF Amanatullah DF . Single-cell mass cytometry reveals cross-talk between inflammation-dampening and inflammation-amplifying cells in osteoarthritic cartilage. Sci Adv. (2020) 6:eaay5352. doi: 10.1126/sciadv.aay5352 32201724 PMC7069698

[227] Kang X Zhang K Wang Y Zhao Y Lu Y . Single-cell RNA sequencing analysis of human chondrocytes reveals cell-cell communication alterations mediated by interactive signaling pathways in osteoarthritis. Front Cell Dev Biol. (2023) 11:1099287. doi: 10.3389/fcell.2023.1099287 37082621 PMC10112522

[228] Chevalier X Goupille P Beaulieu AD Burch FX Bensen WG Conrozier T . Intraarticular injection of anakinra in osteoarthritis of the knee: A multicenter, randomized, double-blind, placebo-controlled study. Arthritis Rheumatism. (2009) 61:344–52. doi: 10.1002/art.24096 19248129

[229] Fleischmann RM Bliddal H Blanco FJ Schnitzer TJ Peterfy C Chen S . A phase II trial of lutikizumab, an anti-interleukin-1α/β dual variable domain immunoglobulin, in knee osteoarthritis patients with synovitis. Arthritis Rheumatol (Hoboken NJ). (2019) 71:1056–69. doi: 10.1002/art.40840 30653843

[230] Kloppenburg M Peterfy C Haugen IK Kroon F Chen S Wang L . Phase IIa, placebo-controlled, randomised study of lutikizumab, an anti-interleukin-1α and anti-interleukin-1β dual variable domain immunoglobulin, in patients with erosive hand osteoarthritis. Ann Rheumatic Dis. (2019) 78:413–20. doi: 10.1136/annrheumdis-2018-213336 30552176 PMC6390132

[231] Richette P Latourte A Sellam J Wendling D Piperno M Goupille P . Efficacy of tocilizumab in patients with hand osteoarthritis: Double blind, randomised, placebo-controlled, multicentre trial. Ann Rheumatic Dis. (2021) 80:349–55. doi: 10.1136/annrheumdis-2020-218547 33055078

[232] Aitken D Laslett LL Pan F Haugen IK Otahal P Bellamy N . A randomised double-blind placebo-controlled crossover trial of Humira (adalimumab) for erosive hand osteoarthritis - the Humor trial. Osteoarthritis Cartilage. (2018) 26:880–7. doi: 10.1016/j.joca.2018.02.899 29499287

[233] Kloppenburg M Ramonda R Bobacz K Kwok WY Elewaut D Huizinga TWJ . Etanercept in patients with inflammatory hand osteoarthritis (Ehoa): A multicentre, randomised, double-blind, placebo-controlled trial. Ann Rheumatic Dis. (2018) 77:1757–64. doi: 10.1136/annrheumdis-2018-213202 30282670

[234] Yu H Li M Wen X Yang J Liang X Li X . Elevation of A-1,3 fucosylation promotes the binding ability of Tnfr1 to Tnf-A and contributes to osteoarthritic cartilage destruction and apoptosis. Arthritis Res Ther. (2022) 24:93. doi: 10.1186/s13075-022-02776-z 35488351 PMC9052622

[235] Lu W He Z Shi J Wang Z Wu W Liu J . Amd3100 attenuates post-traumatic osteoarthritis by maintaining transforming growth factor-B1-induced expression of tissue inhibitor of metalloproteinase-3 via the phosphatidylinositol 3-kinase/Akt pathway. Front Pharmacol. (2019) 10:1554. doi: 10.3389/fphar.2019.01554 32038242 PMC6987846

[236] Huang H Zheng S Wu J Liang X Li S Mao P . Opsonization inveigles macrophages engulfing carrier-free bilirubin/Jph203 nanoparticles to suppress inflammation for osteoarthritis therapy. Advanced Sci (Weinheim Baden-Wurttemberg Germany). (2024) 11:e2400713. doi: 10.1002/advs.202400713 38593402 PMC11165524

[237] Qi W Jin L Huang S Aikebaier A Xue S Wang Q . Modulating synovial macrophage pyroptosis and mitophagy interactions to mitigate osteoarthritis progression using functionalized nanoparticles. Acta Biomater. (2024) 181:425–39. doi: 10.1016/j.actbio.2024.05.014 38729544

