# Supplementary material for: Immune–chondrocyte ligand–receptor axes in osteoarthritis: microenvironmental control of inflammation, matrix remodeling, and chondrocyte fate
Source: Front Immunol. 2026 Oct 23;17:1957574. doi: 10.3389/fimmu.2026.1957574 (PMC13740828; doi:10.3389/fimmu.2026.1957574)
Supplement: Supplementary file 1 [file Table1.docx]

**Table 1. High-priority ligand–receptor axes in immune–chondrocyte and inflammation-associated communication in osteoarthritis.**

| **Ligand–receptor axis** | **Major ligand source(s)** | **Key chondrocyte effect / critical interpretation** | **Evidence support** | **Functional classification** | **Representative reference(s)** |
| --- | --- | --- | --- | --- | --- |
| **IL-1β–IL-1R** | Synovial macrophages; chondrocytes (including stressed/inflamed chondrocytes) | Inflammatory/catabolic activation via NF-κB/MAPK; strong mechanistic evidence, but clinical IL-1 blockade has shown limited benefit. | HT; SL; PH | Pathogenic | (1, 2) |
| **TNF-α–TNFR1/TNFR2** | Macrophages; T cells; mast cells; synovial cells | TNFR1 is mainly catabolic, whereas TNFR2 can be protective; receptor context is therefore critical. | HT; GM; PH | Biphasic | (3, 4) |
| **IL-6–IL-6R/gp130** | Synovial fibroblasts; chondrocytes; synovial membrane; infrapatellar fat pad (IFP) | Promotes catabolism, senescence and pain signaling; gp130 outputs are context- and sex-dependent. | HT; SL; GM; PH | Biphasic | (5-9) |
| **IL-17–IL-17R** | Th17-polarized CD4+ T cells | Promotes chemokine/MMP production and senescence; supported by human chondrocytes, T-cell co-culture, and genetic models. | HT; CC; GM; PH | Pathogenic | (10-13) |
| **CXCL12–CXCR4/CXCR7** | Stromal/subchondral bone cells; endothelial and inflammatory cells | Links osteochondral/stromal compartments to cartilage remodeling; human data support CXCR4 upregulation, but net effects remain context-dependent. | HT; SL; PH | Uncertain | (14, 15) |
| **CCL2–CCR2** | Chondrocytes; fibroblasts; macrophages; endothelial cells | Promotes macrophage recruitment and chondrocyte catabolism; strong human, knockout, and pharmacological support. | HT; SL; GM; PH | Pathogenic | (16, 17) |
| **HMGB1–TLR4/RAGE** | Damaged/stressed chondrocytes; synovial and dying cells | DAMP-driven innate/catabolic signaling; supported by human OA tissue and TLR/MyD88 genetic perturbation. | HT; GM; PH | Pathogenic | (18-20) |
| **S100A8/A9–TLR4/RAGE** | Neutrophils; macrophages; inflamed synovium | Strong TLR4-dependent catabolism in human OA chondrocytes; tissue localization tracks cartilage matrix damage. | HT; SL; PH | Pathogenic | (21) |
| **OPN/SPP1–CD44/integrins** | Macrophages; chondrocytes; synovial/osteochondral cells | Protective CD44–PI3K effects are reported experimentally, but single-cell studies show altered SPP1 signaling; net role remains unresolved. | HT; SL; PH; CP | Uncertain | (22-24) |
| **HA–CD44/TLR2/TLR4** | Cartilage ECM; synovial fluid; fragmented matrix | High-molecular-weight HA is usually protective; small fragments can activate CD44/TLRs, although human findings are inconsistent. | HT; GM; PH | Biphasic | (20, 25-27) |

**Not:** Evidence support: HT, human OA tissue or patient-derived cells; SL, spatial/histologic localization; CC, co-culture; GM, genetic model; PH, pharmacological perturbation; CP, computational prediction. Evidence types are not equivalent; human tissue, spatial localization, direct functional perturbation, and genetic evidence are weighted more heavily than computational prediction or simplified cytokine-stimulation models. Functional classification reflects the dominant interpretation of current evidence and may vary with receptor usage, molecular form, disease stage, and tissue context.

**References**

1.	Towle CA, Hung HH, Bonassar LJ, Treadwell BV, Mangham DC. Detection of Interleukin-1 in the Cartilage of Patients with Osteoarthritis: A Possible Autocrine/Paracrine Role in Pathogenesis. *Osteoarthritis and cartilage* (1997) 5(5):293-300. Epub 1998/03/14. doi: 10.1016/s1063-4584(97)80008-8.

2.	Chevalier X, Goupille P, Beaulieu AD, Burch FX, Bensen WG, Conrozier T, et al. Intraarticular Injection of Anakinra in Osteoarthritis of the Knee: A Multicenter, Randomized, Double-Blind, Placebo-Controlled Study. *Arthritis and rheumatism* (2009) 61(3):344-52. Epub 2009/02/28. doi: 10.1002/art.24096.

3.	Yu H, Li M, Wen X, Yang J, Liang X, Li X, et al. Elevation of Α-1,3 Fucosylation Promotes the Binding Ability of Tnfr1 to Tnf-Α and Contributes to Osteoarthritic Cartilage Destruction and Apoptosis. *Arthritis research & therapy* (2022) 24(1):93. Epub 2022/05/01. doi: 10.1186/s13075-022-02776-z.

4.	Fu W, Hettinghouse A, Chen Y, Hu W, Ding X, Chen M, et al. 14-3-3 Epsilon Is an Intracellular Component of Tnfr2 Receptor Complex and Its Activation Protects against Osteoarthritis. *Annals of the rheumatic diseases* (2021) 80(12):1615-27. Epub 2021/07/07. doi: 10.1136/annrheumdis-2021-220000.

5.	Liao Y, Ren Y, Luo X, Mirando AJ, Long JT, Leinroth A, et al. Interleukin-6 Signaling Mediates Cartilage Degradation and Pain in Posttraumatic Osteoarthritis in a Sex-Specific Manner. *Science signaling* (2022) 15(744):eabn7082. Epub 2022/07/27. doi: 10.1126/scisignal.abn7082.

6.	Shkhyan R, Flynn C, Lamoure E, Sarkar A, Van Handel B, Li J, et al. Inhibition of a Signaling Modality within the Gp130 Receptor Enhances Tissue Regeneration and Mitigates Osteoarthritis. *Science translational medicine* (2023) 15(688):eabq2395. Epub 2023/03/23. doi: 10.1126/scitranslmed.abq2395.

7.	Zhao X, Lin J, Liu F, Zhang Y, Shi B, Ma C, et al. Targeting P21-Positive Senescent Chondrocytes Via Il-6r/Jak2 Inhibition to Alleviate Osteoarthritis. *Advanced science (Weinheim, Baden-Wurttemberg, Germany)* (2025) 12(11):e2410795. Epub 2025/01/24. doi: 10.1002/advs.202410795.

8.	Clockaerts S, Bastiaansen-Jenniskens YM, Feijt C, De Clerck L, Verhaar JA, Zuurmond AM, et al. Cytokine Production by Infrapatellar Fat Pad Can Be Stimulated by Interleukin 1β and Inhibited by Peroxisome Proliferator Activated Receptor Α Agonist. *Annals of the rheumatic diseases* (2012) 71(6):1012-8. Epub 2012/02/07. doi: 10.1136/annrheumdis-2011-200688.

9.	Favero M, El-Hadi H, Belluzzi E, Granzotto M, Porzionato A, Sarasin G, et al. Infrapatellar Fat Pad Features in Osteoarthritis: A Histopathological and Molecular Study. *Rheumatology (Oxford, England)* (2017) 56(10):1784-93. Epub 2017/09/29. doi: 10.1093/rheumatology/kex287.

10.	Honorati MC, Bovara M, Cattini L, Piacentini A, Facchini A. Contribution of Interleukin 17 to Human Cartilage Degradation and Synovial Inflammation in Osteoarthritis. *Osteoarthritis and cartilage* (2002) 10(10):799-807. Epub 2002/10/03. doi: 10.1053/joca.2002.0829.

11.	Hu G, Zhang N, Li J, Wang J, Wu W, Li J, et al. Tumor Necrosis Factor Receptor Associated Factor 3 Modulates Cartilage Degradation through Suppression of Interleukin 17 Signaling. *The American journal of pathology* (2020) 190(8):1701-12. Epub 2020/05/18. doi: 10.1016/j.ajpath.2020.04.016.

12.	Faust HJ, Zhang H, Han J, Wolf MT, Jeon OH, Sadtler K, et al. Il-17 and Immunologically Induced Senescence Regulate Response to Injury in Osteoarthritis. *The Journal of clinical investigation* (2020) 130(10):5493-507. Epub 2020/09/22. doi: 10.1172/jci134091.

13.	Platzer H, Wellbrock M, Pourbozorg G, Mayakrishnan R, Gantz S, Khamees B, et al. In Knee Osteoarthritis, the Production of Cytokines and Metalloproteinases in Presence of Chondrocytes and Cd4(+) T Cells Depends on T Cell Subset: An in Vitro Analysis. *Osteoarthritis and cartilage open* (2025) 7(3):100642. Epub 2025/07/04. doi: 10.1016/j.ocarto.2025.100642.

14.	Qin HJ, Xu T, Wu HT, Yao ZL, Hou YL, Xie YH, et al. Sdf-1/Cxcr4 Axis Coordinates Crosstalk between Subchondral Bone and Articular Cartilage in Osteoarthritis Pathogenesis. *Bone* (2019) 125:140-50. Epub 2019/05/21. doi: 10.1016/j.bone.2019.05.010.

15.	Ruths L, de Hesselle H, Haußner F, Albers S, Harms M, Freitag T, et al. Targeting the Cxcl12/Cxcr4 Pathway by an Optimized Derivative or Epi-X4 Preserves Chondrocyte Function and Offers a Novel Therapeutic Approach in Rheumatic Diseases. *Arthritis research & therapy* (2025) 27(1):222. Epub 2025/12/03. doi: 10.1186/s13075-025-03691-9.

16.	Raghu H, Lepus CM, Wang Q, Wong HH, Lingampalli N, Oliviero F, et al. Ccl2/Ccr2, but Not Ccl5/Ccr5, Mediates Monocyte Recruitment, Inflammation and Cartilage Destruction in Osteoarthritis. *Annals of the rheumatic diseases* (2017) 76(5):914-22. Epub 2016/12/15. doi: 10.1136/annrheumdis-2016-210426.

17.	Willcockson H, Ozkan H, Chubinskaya S, Loeser RF, Longobardi L. Ccl2 Induces Articular Chondrocyte Mmp Expression through Erk and P38 Signaling Pathways. *Osteoarthritis and cartilage open* (2021) 3(1):100136. Epub 2021/01/06. doi: 10.1016/j.ocarto.2020.100136.

18.	Zhou S, Liu G, Si Z, Yu L, Hou L. Glycyrrhizin, an Hmgb1 Inhibitor, Suppresses Interleukin-1β-Induced Inflammatory Responses in Chondrocytes from Patients with Osteoarthritis. *Cartilage* (2021) 13(2_suppl):947s-55s. Epub 2020/07/01. doi: 10.1177/1947603520934858.

19.	Li W, Tao C, Mao M, Zhu K. The Nrf2/Hmgb1/Nf-Κb Axis Modulates Chondrocyte Apoptosis and Extracellular Matrix Degradation in Osteoarthritis. *Acta biochimica et biophysica Sinica* (2023) 55(5):818-30. Epub 2023/05/26. doi: 10.3724/abbs.2023078.

20.	Liu-Bryan R, Terkeltaub R. Chondrocyte Innate Immune Myeloid Differentiation Factor 88-Dependent Signaling Drives Procatabolic Effects of the Endogenous Toll-Like Receptor 2/Toll-Like Receptor 4 Ligands Low Molecular Weight Hyaluronan and High Mobility Group Box Chromosomal Protein 1 in Mice. *Arthritis and rheumatism* (2010) 62(7):2004-12. Epub 2010/05/28. doi: 10.1002/art.27475.

21.	Schelbergen RF, Blom AB, van den Bosch MH, Slöetjes A, Abdollahi-Roodsaz S, Schreurs BW, et al. Alarmins S100a8 and S100a9 Elicit a Catabolic Effect in Human Osteoarthritic Chondrocytes That Is Dependent on Toll-Like Receptor 4. *Arthritis and rheumatism* (2012) 64(5):1477-87. Epub 2011/12/01. doi: 10.1002/art.33495.

22.	Liu Q, Zeng H, Yuan Y, Wang Z, Wu Z, Luo W. Osteopontin Inhibits Osteoarthritis Progression Via the Opn/Cd44/Pi3k Signal Axis. *Genes & diseases* (2022) 9(1):128-39. Epub 2020/06/27. doi: 10.1016/j.gendis.2020.06.006.

23.	Kang X, Zhang K, Wang Y, Zhao Y, Lu Y. Single-Cell Rna Sequencing Analysis of Human Chondrocytes Reveals Cell-Cell Communication Alterations Mediated by Interactive Signaling Pathways in Osteoarthritis. *Frontiers in cell and developmental biology* (2023) 11:1099287. Epub 2023/04/21. doi: 10.3389/fcell.2023.1099287.

24.	Qu Y, Wang Y, Wang S, Yu X, He Y, Lu R, et al. A Comprehensive Analysis of Single-Cell Rna Transcriptome Reveals Unique Spp1+ Chondrocytes in Human Osteoarthritis. *Computers in biology and medicine* (2023) 160:106926. Epub 2023/05/04. doi: 10.1016/j.compbiomed.2023.106926.

25.	Brun P, Panfilo S, Daga Gordini D, Cortivo R, Abatangelo G. The Effect of Hyaluronan on Cd44-Mediated Survival of Normal and Hydroxyl Radical-Damaged Chondrocytes. *Osteoarthritis and cartilage* (2003) 11(3):208-16. Epub 2003/03/08. doi: 10.1016/s1063-4584(02)00352-7.

26.	Campo GM, Avenoso A, Campo S, D'Ascola A, Nastasi G, Calatroni A. Small Hyaluronan Oligosaccharides Induce Inflammation by Engaging Both Toll-Like-4 and Cd44 Receptors in Human Chondrocytes. *Biochemical pharmacology* (2010) 80(4):480-90. Epub 2010/05/04. doi: 10.1016/j.bcp.2010.04.024.

27.	Cowman MK, Shortt C, Arora S, Fu Y, Villavieja J, Rathore J, et al. Role of Hyaluronan in Inflammatory Effects on Human Articular Chondrocytes. *Inflammation* (2019) 42(5):1808-20. Epub 2019/06/28. doi: 10.1007/s10753-019-01043-9.
