# Supplementary material for: Immune–chondrocyte ligand–receptor axes in osteoarthritis: microenvironmental control of inflammation, matrix remodeling, and chondrocyte fate
Source: Front Immunol. 2026 Oct 23;17:1957574. doi: 10.3389/fimmu.2026.1957574 (PMC13740828; doi:10.3389/fimmu.2026.1957574)
Supplement: Supplementary file 2 [file Table2.docx]

**Table 2. Chondrocyte fate transitions controlled by immune–chondrocyte ligand–receptor axes.**

| **Chondrocyte fate transition** | **Main inducing LR axes** | **Key downstream pathways** | **Representative markers** | **Functional consequence in OA** | **Therapeutic implication** | **Representative references** |
| --- | --- | --- | --- | --- | --- | --- |
| **Catabolic activation** | IL-1β–IL-1R; TNF–TNFR; IL-17–IL-17R; IL-6–IL-6R/gp130; HMGB1–TLR4/RAGE; fibronectin fragments–TLR/integrins | NF-κB, MAPK, AP-1, JAK/STAT | MMP13, MMP3, ADAMTS4, ADAMTS5, COX-2, iNOS, IL-6, CCL2, CXCL8 | Accelerates collagen II and aggrecan degradation; suppresses matrix repair | Target cytokine receptors, NF-κB/MAPK, DAMP–PRR signaling | (1-4) |
| **Loss of anabolic cartilage identity** | IL-1β–IL-1R; TNF–TNFR; DAMP–TLR/RAGE; integrin–ECM stress signaling | NF-κB, MAPK, oxidative stress pathways | Reduced COL2A1, ACAN, SOX9 | Impairs matrix synthesis and cartilage homeostasis | Restore SOX9-related anabolic programs; reduce upstream inflammation | (5-8) |
| **Hypertrophic differentiation** | TGF-β–TGFBR; WNT–FZD/LRP; IL-1β–IL-1R; TNF–TNFR; integrin–ECM; CXCL12–CXCR4 | SMAD, β-catenin, MAPK, YAP/TAZ, RUNX2 signaling | COL10A1, RUNX2, MMP13, ALPL, VEGF | Promotes calcification, osteochondral remodeling, osteophyte formation | Stage-specific modulation of TGF-β/WNT/integrin signaling | (9-11) |
| **Senescence** | IL-6–IL-6R/JAK2; IL-17–IL-17R; TNF–TNFR; DAMP–TLR/RAGE; CXCL12–CXCR4 | JAK/STAT, NF-κB, MAPK, ROS/DNA damage responses | p16, p21, SA-β-gal, SASP factors, IL-6, CCL2, MMPs | Sustains chronic inflammation, reduces matrix synthesis, promotes immune recruitment | Senomorphic/senolytic strategies combined with LR-axis inhibition | (12-14) |
| **Apoptosis** | TNF–TNFR1; FasL–Fas; HMGB1/NF-κB-related stress signaling | Caspases, JNK, mitochondrial stress, BAX/BCL2 imbalance | Cleaved caspase-3, BAX, reduced BCL2, TUNEL positivity | Reduces chondrocyte number and matrix-maintaining capacity | Block death receptor overactivation; preserve chondrocyte survival | (4, 15, 16) |
| **Pyroptosis** | DAMP–TLR signaling; IL-1β/IL-18-related inflammasome activation | NF-κB priming, NLRP3 inflammasome, caspase-1, GSDMD | NLRP3, caspase-1, GSDMD-N, IL-1β, IL-18 | Causes inflammatory cell death and DAMP release | Target NLRP3, TLR4/RAGE, IL-1β amplification | (17, 18) |
| **Necroptosis** | TNF–TNFR under apoptosis-impaired conditions | RIPK1, RIPK3, MLKL | p-RIPK3, p-MLKL | Promotes inflammatory membrane rupture and DAMP release | Explore RIPK1/RIPK3/MLKL inhibition in defined OA contexts | (19-21) |
| **Ferroptosis-prone state** | IL-1β/TNF/DAMP-induced oxidative stress; macrophage-derived inflammatory mediators | ROS, lipid peroxidation, iron dysregulation, GPX4/SLC7A11 impairment | Reduced GPX4/SLC7A11, increased lipid ROS, MDA, iron accumulation | Links inflammation, oxidative damage, and chondrocyte death | Antioxidant, anti-ferroptotic, iron-modulating strategies | (22-24) |
| **Autophagy dysfunction** | Chronic IL-1β/TNF signaling; DAMP–PRR activation; mTOR-related stress signaling | NF-κB, MAPK, mTOR, impaired mitophagy | Reduced LC3-II/autophagic flux, p62 accumulation, mitochondrial dysfunction | Increases senescence, apoptosis, oxidative stress, and ECM degradation | Restore physiological autophagy/mitophagy while reducing inflammatory stress | (25-27) |
| **Repair-associated or adaptive state** | OPN/SPP1–CD44/integrins; IL-10–IL-10R; TGF-β–TGFBR; MerTK/GAS6–AXL-related resolution signals | PI3K/AKT, SMAD, anti-inflammatory and pro-resolution signaling | SOX9, COL2A1, ACAN, IL-10-related markers, efferocytosis markers | May support matrix repair, inflammation resolution, and tissue adaptation | Preserve protective communication while blocking pathological signaling | (28-30) |

1.	Bau B, Gebhard PM, Haag J, Knorr T, Bartnik E, Aigner T. Relative Messenger Rna Expression Profiling of Collagenases and Aggrecanases in Human Articular Chondrocytes in Vivo and in Vitro. *Arthritis and rheumatism* (2002) 46(10):2648-57. Epub 2002/10/18. doi: 10.1002/art.10531.

2.	Liacini A, Sylvester J, Li WQ, Huang W, Dehnade F, Ahmad M, et al. Induction of Matrix Metalloproteinase-13 Gene Expression by Tnf-Alpha Is Mediated by Map Kinases, Ap-1, and Nf-Kappab Transcription Factors in Articular Chondrocytes. *Experimental cell research* (2003) 288(1):208-17. Epub 2003/07/25. doi: 10.1016/s0014-4827(03)00180-0.

3.	Hwang HS, Park SJ, Cheon EJ, Lee MH, Kim HA. Fibronectin Fragment-Induced Expression of Matrix Metalloproteinases Is Mediated by Myd88-Dependent Tlr-2 Signaling Pathway in Human Chondrocytes. *Arthritis research & therapy* (2015) 17:320. Epub 2015/11/14. doi: 10.1186/s13075-015-0833-9.

4.	Latourte A, Cherifi C, Maillet J, Ea HK, Bouaziz W, Funck-Brentano T, et al. Systemic Inhibition of Il-6/Stat3 Signalling Protects against Experimental Osteoarthritis. *Annals of the rheumatic diseases* (2017) 76(4):748-55. Epub 2016/11/01. doi: 10.1136/annrheumdis-2016-209757.

5.	Fan Z, Bau B, Yang H, Soeder S, Aigner T. Freshly Isolated Osteoarthritic Chondrocytes Are Catabolically More Active Than Normal Chondrocytes, but Less Responsive to Catabolic Stimulation with Interleukin-1beta. *Arthritis and rheumatism* (2005) 52(1):136-43. Epub 2005/01/11. doi: 10.1002/art.20725.

6.	Zhang Q, Ji Q, Wang X, Kang L, Fu Y, Yin Y, et al. Sox9 Is a Regulator of Adamtss-Induced Cartilage Degeneration at the Early Stage of Human Osteoarthritis. *Osteoarthritis and cartilage* (2015) 23(12):2259-68. Epub 2015/07/15. doi: 10.1016/j.joca.2015.06.014.

7.	Hwang HS, Lee MH, Kim HA. Fibronectin Fragment Inhibits Xylosyltransferase-1 Expression by Regulating Sp1/Sp3- Dependent Transcription in Articular Chondrocytes. *Osteoarthritis and cartilage* (2019) 27(5):833-43. Epub 2019/01/28. doi: 10.1016/j.joca.2019.01.006.

8.	Chen Y, Chen Y, Wang W, Chen J, Tang Q, Wu R, et al. The Mir-302c/Transforming Growth Factor-Β Receptor Type-2 Axis Modulates Interleukin-1β-Induced Degenerative Changes in Osteoarthritic Chondrocytes. *Journal of cell communication and signaling* (2022) 16(1):93-102. Epub 2021/06/15. doi: 10.1007/s12079-020-00591-2.

9.	Yang X, Chen L, Xu X, Li C, Huang C, Deng CX. Tgf-Beta/Smad3 Signals Repress Chondrocyte Hypertrophic Differentiation and Are Required for Maintaining Articular Cartilage. *The Journal of cell biology* (2001) 153(1):35-46. Epub 2001/04/04. doi: 10.1083/jcb.153.1.35.

10.	Li TF, Gao L, Sheu TJ, Sampson ER, Flick LM, Konttinen YT, et al. Aberrant Hypertrophy in Smad3-Deficient Murine Chondrocytes Is Rescued by Restoring Transforming Growth Factor Beta-Activated Kinase 1/Activating Transcription Factor 2 Signaling: A Potential Clinical Implication for Osteoarthritis. *Arthritis and rheumatism* (2010) 62(8):2359-69. Epub 2010/05/28. doi: 10.1002/art.27537.

11.	Thielen NGM, Neefjes M, Vitters EL, van Beuningen HM, Blom AB, Koenders MI, et al. Identification of Transcription Factors Responsible for a Transforming Growth Factor-Β-Driven Hypertrophy-Like Phenotype in Human Osteoarthritic Chondrocytes. *Cells* (2022) 11(7). Epub 2022/04/13. doi: 10.3390/cells11071232.

12.	Si HB, Yang TM, Li L, Tian M, Zhou L, Li DP, et al. Mir-140 Attenuates the Progression of Early-Stage Osteoarthritis by Retarding Chondrocyte Senescence. *Molecular therapy Nucleic acids* (2020) 19:15-30. Epub 2019/12/04. doi: 10.1016/j.omtn.2019.10.032.

13.	Ro DH, Cho GH, Kim JY, Min SK, Yang HR, Park HJ, et al. Selective Targeting of Dipeptidyl-Peptidase 4 (Dpp-4) Positive Senescent Chondrocyte Ameliorates Osteoarthritis Progression. *Aging cell* (2024) 23(7):e14161. Epub 2024/04/01. doi: 10.1111/acel.14161.

14.	Zhao X, Lin J, Liu F, Zhang Y, Shi B, Ma C, et al. Targeting P21-Positive Senescent Chondrocytes Via Il-6r/Jak2 Inhibition to Alleviate Osteoarthritis. *Advanced science (Weinheim, Baden-Wurttemberg, Germany)* (2025) 12(11):e2410795. Epub 2025/01/24. doi: 10.1002/advs.202410795.

15.	Kim HA, Lee YJ, Seong SC, Choe KW, Song YW. Apoptotic Chondrocyte Death in Human Osteoarthritis. *The Journal of rheumatology* (2000) 27(2):455-62. Epub 2000/02/24.

16.	Mistry D, Oue Y, Chambers MG, Kayser MV, Mason RM. Chondrocyte Death During Murine Osteoarthritis. *Osteoarthritis and cartilage* (2004) 12(2):131-41. Epub 2004/01/16. doi: 10.1016/j.joca.2003.10.006.

17.	Zhang L, Qiu J, Shi J, Liu S, Zou H. Microrna-140-5p Represses Chondrocyte Pyroptosis and Relieves Cartilage Injury in Osteoarthritis by Inhibiting Cathepsin B/Nod-Like Receptor Protein 3. *Bioengineered* (2021) 12(2):9949-64. Epub 2021/09/28. doi: 10.1080/21655979.2021.1985342.

18.	Shi J, Zhang Z, Zhang W, Zhao X. Mechanism of the Ddx3x/Nlrp3/Gsdmd Signaling Axis in Pyroptosis and Inflammatory Response in Osteoarthritis Chondrocytes. *Journal of biochemical and molecular toxicology* (2026) 40(1):e70618. Epub 2025/12/23. doi: 10.1002/jbt.70618.

19.	Riegger J, Brenner RE. Evidence of Necroptosis in Osteoarthritic Disease: Investigation of Blunt Mechanical Impact as Possible Trigger in Regulated Necrosis. *Cell death & disease* (2019) 10(10):683. Epub 2019/09/19. doi: 10.1038/s41419-019-1930-5.

20.	Cheng J, Duan X, Fu X, Jiang Y, Yang P, Cao C, et al. Rip1 Perturbation Induces Chondrocyte Necroptosis and Promotes Osteoarthritis Pathogenesis Via Targeting Bmp7. *Frontiers in cell and developmental biology* (2021) 9:638382. Epub 2021/05/04. doi: 10.3389/fcell.2021.638382.

21.	Piao L, Wu D, Rui C, Yang Y, Liu S, Liu J, et al. The Bcr-Abl Inhibitor Dcc-2036 Inhibits Necroptosis and Ameliorates Osteoarthritis by Targeting Ripk1 and Ripk3 Kinases. *Biomedicine & pharmacotherapy = Biomedecine & pharmacotherapie* (2023) 161:114528. Epub 2023/03/18. doi: 10.1016/j.biopha.2023.114528.

22.	Wang X, Liu Z, Peng P, Gong Z, Huang J, Peng H. Astaxanthin Attenuates Osteoarthritis Progression Via Inhibiting Ferroptosis and Regulating Mitochondrial Function in Chondrocytes. *Chemico-biological interactions* (2022) 366:110148. Epub 2022/09/10. doi: 10.1016/j.cbi.2022.110148.

23.	Ruan Q, Wang C, Zhang Y, Sun J. Ruscogenin Attenuates Cartilage Destruction in Osteoarthritis through Suppressing Chondrocyte Ferroptosis Via Nrf2/Slc7a11/Gpx4 Signaling Pathway. *Chemico-biological interactions* (2024) 388:110835. Epub 2023/12/21. doi: 10.1016/j.cbi.2023.110835.

24.	Zeng L, Liu Y, Wang Q, Wan H, Meng X, Tu P, et al. Botulinum Toxin a Attenuates Osteoarthritis Development Via Inhibiting Chondrocyte Ferroptosis through Slc7al1/Gpx4 Axis. *Biochimica et biophysica acta Molecular basis of disease* (2024) 1870(5):167215. Epub 2024/05/08. doi: 10.1016/j.bbadis.2024.167215.

25.	Caramés B, Hasegawa A, Taniguchi N, Miyaki S, Blanco FJ, Lotz M. Autophagy Activation by Rapamycin Reduces Severity of Experimental Osteoarthritis. *Annals of the rheumatic diseases* (2012) 71(4):575-81. Epub 2011/11/16. doi: 10.1136/annrheumdis-2011-200557.

26.	Zhang Y, Vasheghani F, Li YH, Blati M, Simeone K, Fahmi H, et al. Cartilage-Specific Deletion of Mtor Upregulates Autophagy and Protects Mice from Osteoarthritis. *Annals of the rheumatic diseases* (2015) 74(7):1432-40. Epub 2014/03/22. doi: 10.1136/annrheumdis-2013-204599.

27.	Gu Y, Yan R, Wang Y, Zeng Y, Yao Q. High Trb3 Expression Induces Chondrocyte Autophagy and Senescence in Osteoarthritis Cartilage. *Aging* (2022) 14(13):5366-75. Epub 2022/07/02. doi: 10.18632/aging.204066.

28.	Iannone F, De Bari C, Dell'Accio F, Covelli M, Cantatore FP, Patella V, et al. Interleukin-10 and Interleukin-10 Receptor in Human Osteoarthritic and Healthy Chondrocytes. *Clinical and experimental rheumatology* (2001) 19(2):139-45. Epub 2001/05/03.

29.	Liu Q, Zeng H, Yuan Y, Wang Z, Wu Z, Luo W. Osteopontin Inhibits Osteoarthritis Progression Via the Opn/Cd44/Pi3k Signal Axis. *Genes & diseases* (2022) 9(1):128-39. Epub 2020/06/27. doi: 10.1016/j.gendis.2020.06.006.

30.	Yao Z, Qi W, Zhang H, Zhang Z, Liu L, Shao Y, et al. Down-Regulated Gas6 Impairs Synovial Macrophage Efferocytosis and Promotes Obesity-Associated Osteoarthritis. *eLife* (2023) 12. Epub 2023/05/05. doi: 10.7554/eLife.83069.
