# Supplementary material for: Immune–chondrocyte ligand–receptor axes in osteoarthritis: microenvironmental control of inflammation, matrix remodeling, and chondrocyte fate
Source: Front Immunol. 2026 Oct 23;17:1957574. doi: 10.3389/fimmu.2026.1957574 (PMC13740828; doi:10.3389/fimmu.2026.1957574)
Supplement: Supplementary file 3 [file Table3.docx]

**Supplementary Table S3. Representative single-cell, spatial, and cell–cell communication studies in osteoarthritis.**

| **First author, year** | **OA tissue / cohort** | **Platform / analysis** | **Major cell populations / focus** | **Key OA finding** | **Cell–cell / ligand–receptor finding and validation** | **Representative references** |
| --- | --- | --- | --- | --- | --- | --- |
| Ji et al., 2019 | Human knee OA cartilage; 1,464 chondrocytes from 10 patients undergoing arthroplasty | scRNA-seq; trajectory analysis; histologic validation | Seven chondrocyte populations, including proliferative, prehypertrophic, hypertrophic, fibrocartilage, regulatory, and progenitor-associated states | Established transcriptionally distinct chondrocyte states and potential lineage transitions during OA progression. | No direct LR inference; provides a foundational cell-state framework for subsequent communication analyses. | (1) |
| Chou et al., 2020 | Matched human OA synovium and cartilage; 10,640 synoviocytes and 26,192 chondrocytes | scRNA-seq; cross-tissue cytokine/growth-factor analysis | 12 synovial cell types and 7 chondrocyte phenotypes | Damaged cartilage was enriched for fibrocartilage, reparative, regulatory, and prehypertrophic chondrocytes. | Predicted 61 cytokine/growth-factor regulators of chondrocyte states; >50% were produced by synovial populations. IL-1β-producing synovial cells were mainly inflammatory macrophages and dendritic cells; selected mediators were confirmed in OA synovial fluid. | (2) |
| Huang et al., 2022 | Human knee OA synovium from 17 patients undergoing total knee arthroplasty | scRNA-seq; paired bulk RNA-seq; in silico deconvolution | Fibroblasts, antigen-presenting cells, T cells, endothelial cells, mural cells, B cells, and mast cells | Defined major synovial cell populations and showed marked cellular heterogeneity in OA synovium. | Did not directly test LR signaling; provides a synovial cellular reference and validates deconvolution approaches for larger OA cohorts. | (3) |
| Kang et al., 2023 | Publicly available human OA cartilage scRNA-seq datasets; damaged versus intact cartilage | scRNA-seq reanalysis; CellChat; bulk RNA-seq validation | Seven chondrocyte subtypes | Overall cell-cell communication frequency and strength were increased in damaged cartilage. | PTN, VISFATIN, SPP1, and TGF-β signaling were selectively altered among chondrocyte subtypes. Findings are primarily computational and require direct functional validation. | (4) |
| Tang et al., 2024 | Human infrapatellar fat pad (IFP) and synovium from healthy and OA knees | scRNA-seq and snRNA-seq; trajectory and communication analysis; ex vivo and in vivo validation | Mesenchymal-lineage cells, fibroblasts, adipocytes, macrophages, and other IFP/synovial populations | OA induced coordinated profibrotic and inflammatory changes in the IFP-synovium unit, with BGN+ intermediate fibroblasts contributing to fibrosis. | APOE signaling from intermediate fibroblasts and macrophages was implicated in cartilage damage. Soluble APOE accelerated proteoglycan loss in human cartilage ex vivo, whereas anti-APOE neutralization attenuated experimental OA in mice. | (5) |
| Fan et al., 2024 | Human knee cartilage; 8 OA donors and 3 non-OA donors (19 samples) | scRNA-seq; spatial transcriptomics; IHC/qPCR; multi-omics integration | 11 chondrocyte populations, including pre-inflammatory and inflammatory chondrocytes | OA-associated cell states and transcriptional changes were spatially enriched at the articular surface/superficial cartilage. | Inflammatory chondrocytes were associated with MIF-CD74 signaling. Spatial localization and orthogonal validation strengthen the anatomical relevance, although direct receptor perturbation was not the primary design. | (6) |
| Pu et al., 2024 | Human IFP; 5 OA and 5 healthy-control samples; 49,674 cells | scRNA-seq and snRNA-seq; trajectory and cell-cell communication analysis; multiplex IHC | 11 major cell types; adipose stem/progenitor cells and interstitial inflammatory fibroblasts (iiFBs) | iiFBs showed strong inflammatory/proliferative features and represented an OA-associated IFP fibroblast state. | iiFBs were predicted to modulate macrophage and T-cell activity and to influence cartilage-related responses through the midkine (MDK) pathway; key cellular states/pathways were supported by multiplex IHC. | (7) |
| Liu et al., 2024 | Public human OA synovial and cartilage single-cell datasets | Single-cell reanalysis; cell-interaction analysis | CILP+ and POSTN+ synovial fibroblast subpopulations; damaged and non-damaged chondrocytes | CILP+ fibroblasts showed a repair-associated profile, whereas POSTN+ fibroblasts were associated with inflammatory and matrix-destructive programs. | Predicted stronger CILP+ fibroblast interactions with non-damaged chondrocytes and POSTN+ fibroblast interactions with damaged chondrocytes. Evidence is computational and requires spatial/functional confirmation. | (8) |
| Raut et al., 2025 | Human knee cartilage, meniscus, synovium, and subchondral bone | Multi-tissue scRNA-seq atlas; cell-cell communication analysis | Chondrocytes, tissue stem/progenitor cells, fibroblasts, macrophages, and other immune/stromal populations across four joint compartments | OA was characterized by loss of regenerative tissue stem cells, expansion of osteogenic states, MMP13-producing detrimental chondrocytes, and inflammatory/pain-associated macrophages. | Cross-tissue communication analysis identified extensive OA-associated interactions among chondrocytes, macrophages, fibroblasts, and tissue stem/progenitor populations, supporting a whole-joint communication model. | (9) |
| Kulzhanova et al., 2026 | Human hip synovium; 6 femoroacetabular impingement and 6 hip OA patients | Integrated scRNA-seq and spatial transcriptomics; pseudotime and cell-cell interaction analysis | EREG+ lining fibroblasts and COL1A1+IGFBP5+ fibrotic macrophages | EREG+ inflammatory lining fibroblasts were expanded in hip OA and expressed CXCL1, IL8, and MMPs. | Spatially supported FGF2-SDC4 communication was identified between fibrotic macrophages and EREG+ fibroblasts, with downstream inflammatory and angiogenic programs. This represents immune-stromal relay signaling rather than direct macrophage-chondrocyte communication. | (10) |

Abbreviations: OA, osteoarthritis; scRNA-seq, single-cell RNA sequencing; snRNA-seq, single-nucleus RNA sequencing; IFP, infrapatellar fat pad; IHC, immunohistochemistry; LR, ligand–receptor; FLS, fibroblast-like synoviocyte; MDK, midkine.

Interpretive note: Computational cell–cell/LR inference identifies candidate communication pathways but does not by itself establish ligand secretion, receptor activation, or causal signaling. Studies with spatial localization, protein-level validation, ex vivo testing, or in vivo perturbation provide stronger support for functional communication.

**References**

1.	Ji Q, Zheng Y, Zhang G, Hu Y, Fan X, Hou Y, et al. Single-Cell Rna-Seq Analysis Reveals the Progression of Human Osteoarthritis. *Annals of the rheumatic diseases* (2019) 78(1):100-10. Epub 2018/07/22. doi: 10.1136/annrheumdis-2017-212863.

2.	Chou CH, Jain V, Gibson J, Attarian DE, Haraden CA, Yohn CB, et al. Synovial Cell Cross-Talk with Cartilage Plays a Major Role in the Pathogenesis of Osteoarthritis. *Scientific reports* (2020) 10(1):10868. Epub 2020/07/04. doi: 10.1038/s41598-020-67730-y.

3.	Huang ZY, Luo ZY, Cai YR, Chou CH, Yao ML, Pei FX, et al. Single Cell Transcriptomics in Human Osteoarthritis Synovium and in Silico Deconvoluted Bulk Rna Sequencing. *Osteoarthritis and cartilage* (2022) 30(3):475-80. Epub 2022/01/01. doi: 10.1016/j.joca.2021.12.007.

4.	Kang X, Zhang K, Wang Y, Zhao Y, Lu Y. Single-Cell Rna Sequencing Analysis of Human Chondrocytes Reveals Cell-Cell Communication Alterations Mediated by Interactive Signaling Pathways in Osteoarthritis. *Frontiers in cell and developmental biology* (2023) 11:1099287. Epub 2023/04/21. doi: 10.3389/fcell.2023.1099287.

5.	Tang S, Yao L, Ruan J, Kang J, Cao Y, Nie X, et al. Single-Cell Atlas of Human Infrapatellar Fat Pad and Synovium Implicates Apoe Signaling in Osteoarthritis Pathology. *Science translational medicine* (2024) 16(731):eadf4590. Epub 2024/01/24. doi: 10.1126/scitranslmed.adf4590.

6.	Fan Y, Bian X, Meng X, Li L, Fu L, Zhang Y, et al. Unveiling Inflammatory and Prehypertrophic Cell Populations as Key Contributors to Knee Cartilage Degeneration in Osteoarthritis Using Multi-Omics Data Integration. *Annals of the rheumatic diseases* (2024) 83(7):926-44. Epub 2024/02/08. doi: 10.1136/ard-2023-224420.

7.	Pu H, Gao C, Zou Y, Zhao L, Li G, Liu C, et al. Single Cell Transcriptome Profiling of Infrapatellar Fat Pad Highlights the Role of Interstitial Inflammatory Fibroblasts in Osteoarthritis. *International immunopharmacology* (2024) 131:111888. Epub 2024/03/25. doi: 10.1016/j.intimp.2024.111888.

8.	Liu Z, Sun Y, Pan J, Guo K, Tang Z, Wang X. Single-Cell Profiling Uncovers Synovial Fibroblast Subpopulations Associated with Chondrocyte Injury in Osteoarthritis. *Frontiers in endocrinology* (2024) 15:1479909. Epub 2024/12/25. doi: 10.3389/fendo.2024.1479909.

9.	Raut RD, Chakraborty AK, Neogi T, Albro M, Snyder B, Schaer TP, et al. A Multi-Tissue Human Knee Single-Cell Atlas Identifies That Osteoarthritis Reduces Regenerative Tissue Stem Cells While Increasing Inflammatory Pain Macrophages. *Communications biology* (2025) 8(1):1146. Epub 2025/08/03. doi: 10.1038/s42003-025-08586-8.

10.	Kulzhanova G, Klee A, Botros M, Hansen VL, Reuter J, Tashbib ET, et al. Integrative Single-Cell Rna Sequencing and Spatial Transcriptomics Uncover Distinct Fibroblast-Macrophage Crosstalk in Human Hip Synovium between Patients with Femoroacetabular Impingement and Osteoarthritis. *Arthritis & rheumatology (Hoboken, NJ)* (2026) 78(6):1219-30. Epub 2026/01/12. doi: 10.1002/art.70033.
